# Dynamic hierarchical ligand anisotropy for competing macrophage regulation *in vivo*

**DOI:** 10.1016/j.bioactmat.2025.01.009

**Published:** 2025-01-19

**Authors:** Kanghyeon Kim, Sunhong Min, Ramar Thangam, Kyong-Ryol Tag, Hyun-Jeong Lee, Jeongyun Heo, Hwapyung Jung, Thet Thet Swe, Iman Zare, Guosheng Song, Alireza Hassani Najafabadi, Junmin Lee, Hyun-Do Jung, Jong Seung Kim, Sunghoon Hur, Hyun-Cheol Song, Sung-Gyu Park, Kunyu Zhang, Pengchao Zhao, Liming Bian, Se Hoon Kim, Juyoung Yoon, Jae-Pyoung Ahn, Hong-Kyu Kim, Heemin Kang

**Affiliations:** a Department of Materials Science and Engineering, Korea University, Seoul, 02841, Republic of Korea; b Advanced Analysis Center, Korea Institute of Science and Technology (KIST), Seoul, 02792, Republic of Korea; c Center for Theragnosis, Korea Institute of Science and Technology (KIST), Seoul, 02792, Republic of Korea; d Research and Development Department, Sina Medical Biochemistry Technologies Co., Ltd., Shiraz, 7178795844, Iran; e State Key Laboratory of Chemo/Biosensing and Chemometrics, College of Chemistry and Chemical Engineering, Hunan University, Changsha, 410082, China; f Terasaki Institute for Biomedical Innovation, Los Angeles, CA, 90064, USA; g Department of Materials Science and Engineering, Pohang University of Science and Technology (POSTECH), Pohang, 790-784, Republic of Korea; h Division of Materials Science and Engineering, Hanyang University, Seoul, 04763, Republic of Korea; i Department of Chemistry, Korea University, Seoul, 02841, Republic of Korea; j Electronic Materials Research Center, Korea Institute of Science and Technology (KIST), Seoul, 02792, Republic of Korea; k KHU-KIST Department of Converging Science and Technology, Kyung Hee University, Yongin, 17104, Republic of Korea; l School of Advanced Materials Science and Engineering, Sungkyunkwan University (SKKU), Suwon, 16419, Republic of Korea; m KIST-SKKU Carbon-Neutral Research Center, Sungkyunkwan University (SKKU), Suwon, 16419, Republic of Korea; n Department of Nano-Bio Convergence, Korea Institute of Materials Science (KIMS), Changwon, Gyeongnam, 51508, Republic of Korea; o Department of Future Convergence Materials, Korea University, Seoul, 02841, Republic of Korea; p School of Biomedical Sciences and Engineering, Guangzhou International Campus, South China University of Technology, Guangzhou, 511442, China; q KU-KIST Graduate School of Converging Science and Technology, Korea University, Seoul, 02841, Republic of Korea; r Department of Chemistry and Nanoscience, Ewha Womans University, Seoul, 03760, Republic of Korea; s Graduate Program in Innovative Biomaterials Convergence, Ewha Womans University, Seoul, 03760, Republic of Korea; t College of Medicine, Korea University, Seoul, 02841, Republic of Korea

**Keywords:** Hierarchical ligand nanostructure, Multi-scale ligand anisotropy, Remote manipulation, Reversible macrophage regulation

## Abstract

Diverse connective tissues exhibit hierarchical anisotropic structures that intricately regulate homeostasis and tissue functions for dynamic immune response modulation. In this study, remotely manipulable hierarchical nanostructures are tailored to exhibit multi-scale ligand anisotropy. Hierarchical nanostructure construction involves coupling liganded nanoscale isotropic/anisotropic Au (comparable to few integrin molecules-scale) to the surface of microscale isotropic/anisotropic magnetic Fe_3_O_4_ (comparable to integrin cluster-scale) and then elastically tethering them to a substrate. Systematic independent tailoring of nanoscale or microscale ligand isotropy versus anisotropy in four different hierarchical nanostructures with constant liganded surface area demonstrates similar levels of integrin molecule bridging and macrophage adhesion on the nanoscale ligand isotropy versus anisotropy. Conversely, the levels of integrin cluster bridging across hierarchical nanostructures and macrophage adhesion are significantly promoted by microscale ligand anisotropy compared with microscale ligand isotropy. Furthermore, microscale ligand anisotropy dominantly activates the host macrophage adhesion and pro-regenerative M2 polarization *in vivo* over the nanoscale ligand anisotropy, which can be cyclically reversed by substrate-proximate versus substrate-distant magnetic manipulation. This unprecedented scale-specific regulation of cells can be diversified by unlimited tuning of the scale, anisotropy, dimension, shape, and magnetism of hierarchical structures to decipher scale-specific dynamic cell-material interactions to advance immunoengineering strategies.

## Highlights

• Multi-scale ligand anisotropy control via hierarchical anisotropic nanostructure.
• Microscale ligand anisotropy enhances M2 polarization and immune response.
• Scale-specific effect of ligand anisotropy on macrophages can be cyclically reversed.
• Dynamic cell-material interaction for macrophage regulation *in vivo*.

## Graphical abstract

**Figure:**
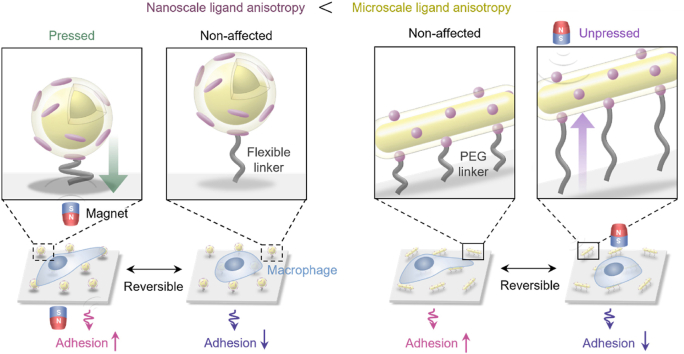

## 1 Introduction

Diverse natural connective tissues are organized in the hierarchical anisotropic arrangement of cells and the extracellular matrix (ECM), where such structure regulates homeostasis and immune functions in tissues [[1], [2], [3], [4]]. Depending on the structure and dynamicity of the tissues, cells dynamically interact with the proteins in the ECM [[5], [6], [7], [8]] that regulate various cellular functions, such as their adhesion [[9], [10], [11]], proliferation [12,13], migration [14,15], and polarization [[16], [17], [18]] to support specific tissue functions. For instance, collagen, a cell-adhesive protein in the ECM, is hierarchically assembled from molecules into fibrils at the nanoscale [19,20], and then into fibers and bundles at the microscale [21,22] that impart anisotropic functionality to tissues [[23], [24], [25]] such as bones [26], tendons [27,28], or muscles [29,30]. Various traumatic injuries or the physiological microenvironment can trigger scale-specific changes of such hierarchical anisotropic structures, which can develop various diseases [31,32]. Changes in their arrangement can trigger immune reactions *in vivo* by altering their interaction with immune cells such as macrophages [[33], [34], [35], [36]].

The decrease in the nanoscale ligand anisotropy of hierarchically structured bone with collagen fibrils [20] reduces the bone's mechanical strength [37,38] and activates inflammatory response for bone resorption, resulting in osteoporosis [39]. Microscale failure of collagen in the bone marrow leads to structural changes, which cause osteosclerosis [40] via fibrosis or aplastic anemia via dysregulation of hematopoiesis [33,41]. The nanoscale disorganization of anisotropic collagen fibrils in tendons leads to tendon degeneration, resulting in tendinopathy [42], whereas the breakdown of microscale collagen bundles can be found upon tendon rupture [43], both of which evoke an immune response in the body [[44], [45], [46]]. The dynamic interaction between a few integrin receptors at the nanoscale (10–100 nm in diameter) in macrophages and ligands in the native ECM can induce the clustering of integrin receptors at the microscale (0.1–1.5 μm in diameter) [2,47] for the regulation of the adhesion structure assembly [47] in macrophages [48,49], triggering their polarization into round-shaped pro-inflammatory M1 (inflammation) or elongated pro-regenerative M2 (tissue repair) phenotypes [50] with the involvement of ROCK signaling [51]. Synergistically, these prior studies have highlighted the necessity of developing dynamically manipulable materials exhibiting hierarchical ligand anisotropy to regulate and elucidate the intricate cell-ECM interactions *in vivo*.

To date, numerous materials with various ligand arrangements and structures have been developed to regulate cellular response. In these studies, the adhesion of cells was regulated by modulating the ligand inter-spacing/density [[52], [53], [54]], ligand ordering [55,56], and anisotropic nanostructure [57]. The immune cells were activated by modulating the protein ligand-covered 2D/3D arrays [58,59] or biomaterials [60,61]. Moreover, cell fates were regulated by statically modulating the anisotropic ligand shape [62], anisotropic ligand pattern [63,64], anisotropic geometry of tissue [65], and anisotropic material shape [66,67], all of which were restricted to a single scale or lacked dynamic regulation. Hierarchical nanostructures exhibiting dynamic multi-scale ligand anisotropy can precisely mimic the native environment and help to decipher cellular response to scale-specific ligand anisotropy, which has never been addressed before.

**Scheme 1:**
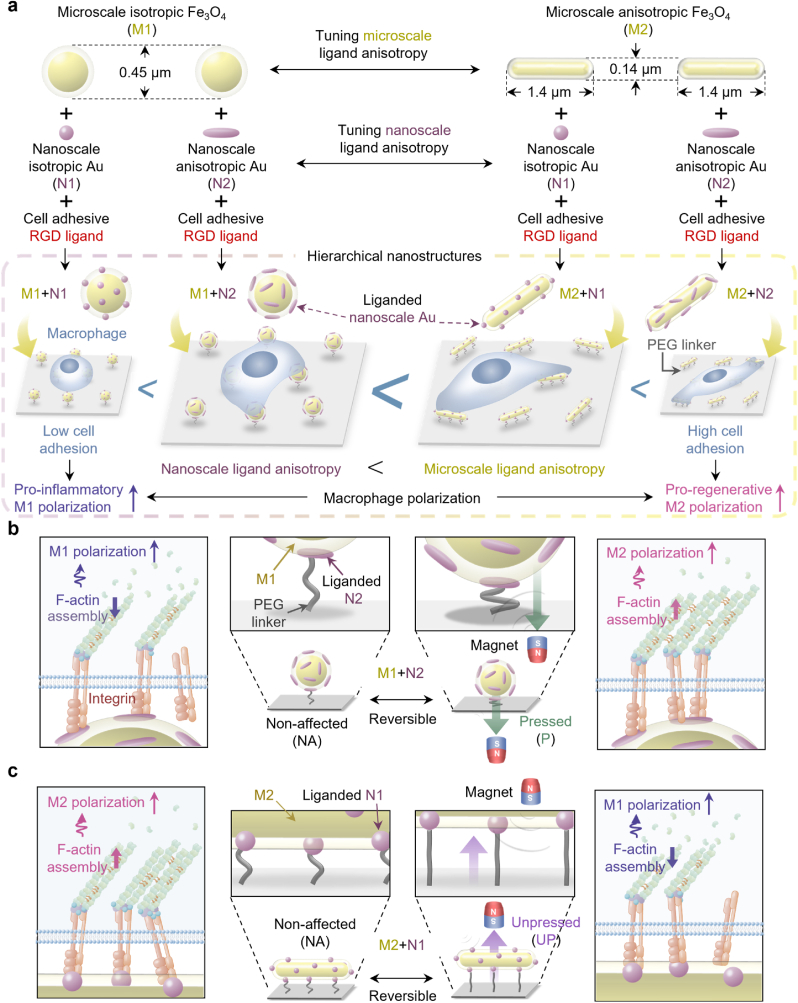
Independent tailoring of multi-scale ligand anisotropy in magnetically manipulable hierarchical nanostructures for dynamic macrophage regulation. (a) Nanoscale-anisotropy-tailored isotropic RGD liganded-Au (N1) spheres or anisotropic liganded-Au (N2) rods are independently coupled on surfaces of a magnetic microscale-anisotropy-tailored isotropic Fe_3_O_4_ (M1) sphere or an anisotropic Fe_3_O_4_ (M2) rod. They constitute magnetically manipulable multi-scale ligand-anisotropy-tailored hierarchical nanostructures (“M1+N1”, “M1+N2”, “M2+N1”, and “M2+N2” groups) that are elastically conjugated to the substrate surface via polymer linkers. The increase in the ligand anisotropy at the microscale (integrin cluster-scale) is significantly more effective than that at the nanoscale (a few integrin molecules-scale) in promoting the adhesion and pro-regenerative M2 polarization of macrophages. (b) Reversible downward (substrate-proximate) manipulation of the “M1+N2” group by placing a permanent magnet under the substrate [“Pressed (P)”] facilitates stable macrophage integrin binding on the nano-anisotropic liganded Au (N2) to increase their adhesion leading to pro-regenerative M2 polarization. (c) By contrast, reversible upward (substrate-distant) manipulation of the “M2+N1” group by placing the permanent magnet over the substrate [“Unpressed (UP)”] suppresses stable macrophage integrin binding on the nano-isotropic liganded Au (N1) to decrease their adhesion, leading to pro-inflammatory M1 polarization.

Herein, we develop remotely manipulable materials exhibiting hierarchical ligand anisotropy in various combinations of liganded nanoscale isotropic or anisotropic Au (N1 or N2, respectively) on magnetic microscale isotropic or anisotropic Fe_3_O_4_ (M1 or M2, respectively), both of which were tailored for their anisotropy from spherical to rod morphology at a fixed surface area in their respective scales (Scheme 1a). The anisotropy-tailored nanoscale Au (N-Au; N1 or N2) particles was coated onto microscale Fe_3_O_4_ (M-Fe_3_O_4_; M1 or M2) that constructed four different multi-scale ligand-anisotropy-tailored hierarchical nanostructures at the constant number and surface areas of Au and Fe_3_O_4_ across the groups (hereafter referred to as “M1+N1”, “M1+N2”, “M2+N1”, and “M2+N2” groups). Their respective nanostructures were coupled on the substrate surfaces at the fixed density among the groups using elastic polymer linkers. Without any further manipulation, we reveal that the effect of ligand multi-scale anisotropy at the microscale (integrin cluster-scale) is dominant over that at the nanoscale (a few integrin molecules-scale) in the adhesion-dependent phenotypic polarization of macrophage *in vivo*. Specifically, the increase of microscale anisotropy promotes adhesion-dependent pro-regenerative M2 polarization, while the increase of nanoscale isotropy promotes pro-inflammatory M1 polarization.

10.13039/100014337Furthermore, magnetic downward movement of the hierarchical nanostructure exhibiting microscale isotropy and nanoscale anisotropy (“M1+N2”) into the “Pressed” (P) state allowed macrophage integrin to stably recognize and hence bind more firmly to the liganded N2, thereby supporting the adhesion structure and following pro-regenerative M2 polarization, both of which were originally hindered in its “Non-affected” (10.13039/501100011632NA) state (Scheme 1b). By contrast, magnetic upward movement of the hierarchical nanostructure exhibiting microscale anisotropy and nanoscale isotropy (“M2+N1”) into the “Unpressed” (UP) state hindered the stable binding of macrophage integrin resulting in decreased adhesion, thereby yielding pro-inflammatory M1 polarization, both of which were originally promoted in its “NA” state (Scheme 1c). Such a regulatory mechanism of macrophage adhesion and subsequent phenotypic polarization observed *in vitro* was consistently translated into *in vivo* applications.

Among the various external stimuli [[68], [69], [70]] harnessed for the remote manipulation of materials *in vivo*, a magnetic field exhibiting highly tissue-penetrative characteristics serves as an adequate stimulus that ensures safe and spatiotemporal control over cytotoxic and tissue-absorptive light [[71], [72], [73], [74]]. Previous studies of our group have reported dynamic regulation of cells [70] via the magnetic modulation of the nano-coupling [75], nano-sequencing [76], and nano-arrangement [77] of ligands. However, none of these preceding studies focused on the effect of scale-specific ligand anisotropy on cellular response. In this study, we present the remote manipulation of hierarchical ligand anisotropy for the regulation of host macrophages for adhesion-mediated pro-regenerative tissue repair [78].

## 2 Materials and methods

### 2.1 Independent tailoring of nanoscale anisotropy of gold (Au)

For the tailoring of nanoscale ligand anisotropy exhibiting similar surface areas, the type and amount of surfactants as well as the amount of precursors were modulated to separately synthesize a nano-isotropic Au (N1) sphere and a nano-anisotropic Au (N2) rod that can readily present anisotropy-controlled ligands. Before their synthesis, the target nanoscale dimensions of the N1 and N2 were calculated so that their anisotropy could be independently tailored while presenting equivalent surface areas.

The anisotropies (aspect ratio: length along major axis/length along minor axis) of N1 and N2 were roughly calculated as 1 and 6.6 (approximately 7), respectively.

The surface area was mathematically calculated as follows.

(1) The surface area of the 45 nm diameter (D) N1 (sphere): 4π(D/2)^2^
(2) The surface area of N2 (rod) in 118 nm length (L) and 18 nm diameter (d): 4π(D/2)^2^ + [(L - D) × 2π(D/2)]

The nanoscale anisotropy of Au was precisely tailored while satisfying (1) = (2).

To synthesize N1 with a diameter of 45 nm, 30 mL of 1 mM hydrogen tetrachloroaurate(III) trihydrate (HAuCl_4_·3H_2_O) in deionized (DI) water was first stirred at 100 °C for 30 min. Afterward, 1.2 mL of 38.8 mM trisodium citrate (Na_3_C_6_H_5_O_7_) in DI water, which acts as a reducing agent and stabilizer, was added and stirred at 100 °C for another 15 min. Termination of the reaction followed by cooling of the mixture solution to 25 °C changed the color of the solution from yellow to burgundy red, resulting in the suspension of 45 nm nano-isotropic Au (N1).

To synthesize nano-anisotropic Au (N2) with a length of 118 nm and a diameter of 18 nm via seed-mediated growth, a gold seed solution was first prepared by mixing 2.5 mL of 0.5 mM HAuCl_4_·3H_2_O in DI water, 2.5 mL of 200 mM hexadecyltrimethylammonium bromide (CTAB) in DI water, and 1.0 mL of 6.0 mM sodium borohydride (NaBH_4_) in DI water under vigorous stirring for 2 min and then leaving the resulting solution undisturbed at 30 °C for 30 min. The acquired gold seed solution was subjected to seed-mediated growth into N2. To this end, a binary surfactant mixture of 25 mL DI water containing 0.8 g CTAB and 0.1234 g sodium oleate was utilized, to which 2.4 mL of 4.0 mM silver nitrate (AgNO_3_) was added and left undisturbed for 15 min. Subsequently, 25 mL of 1 mM HAuCl_4_·3H_2_O in DI water was added to this solution and stirred for 90 min, then added 360 μL of HCl and stirred for another 15 min. Finally, the reaction mixture was quickly injected with 125 μL of 64 mM ascorbic acid (C₆H₈O₆) in DI water and stirred for 30 s. The resultant mixture was added with 40 μL of the gold seed solution, stirred for 30 s, and then left undisturbed at 30 °C for 12 h which resulted in the seed-mediated growth of gold seeds into CTAB-capped nano-anisotropic Au (N2) with 118 nm length and 18 nm diameter.

For their various applications, the CTAB-capped N2 was subjected to serial ligand exchange in which the CTAB on N2 surfaces was first replaced with polystyrene sulfonate (PSS) and then finally with citrate. First, 45 mL of CTAB-capped N2 was centrifuged for the removal of supernatants, then dispersed in 45 mL of 0.2 mM PSS solution and left undisturbed for 1 h for their surfaces to be replaced with PSS that acts as the detergent for CTAB. Serially, 45 mL of PSS-stabilized N2 was centrifuged for the removal of supernatants, then dispersed in 45 mL of 5 mM Na_3_C_6_H_5_O_7_ for the surface of N2 to be covered, and hence be stabilized with citrate.

### 2.2 Independent tailoring of the microscale anisotropy of magnetic Fe_3_O_4_

For the tailoring of microscale ligand anisotropy at similar surface areas that are presented to cells, the nanocore type and shell dimension with phase transformation were modulated to separately synthesize micro-isotropic Fe_3_O_4_ (M1) spheres and micro-anisotropic Fe_3_O_4_ (M2) rods. They can be magnetically manipulated and serve as templates that are coupled with the anisotropy-tailored nanoscale Au. Before their synthesis, the target microscale dimensions of each M1 and M2 were calculated so that their anisotropy could be independently tailored at equivalent surface areas. Mathematical calculations within the desired scale revealed that the surface area of the 450 nm diameter (D) M1 sphere [4π(D/2)^2^] was equivalent to that of the 1400 nm length (L) and 140 nm diameter (D) M2 rod [4π(D/2)^2^ + {(L - D) × 2π(D/2)}].

To synthesize M1 with a diameter of 450 nm, magnetic Fe_3_O_4_ nanoparticles that can be assembled into M1 were first prepared. Initially, 20 mL of octadecene was added with 1.8 mL oleic acid and then heated to 100 °C while being stirred. Sequentially, the mixture solution was added with 0.4 mL of iron(0) pentacarbonyl [Fe(CO)_5_], heated at 180 °C for 1 h, cooled down to 70 °C for 30 min, and then re-heated at 295 °C for 2 h under a nitrogen atmosphere. The resultant Fe_3_O_4_ nanoparticles were cooled down to 25 °C, washed with acetone via centrifugation, and then dispersed in 15 mL of chloroform (approximately 20 mg/mL). To achieve the microscale size, the Fe_3_O_4_ nanoparticles were assembled. To this end, 1 mL of Fe_3_O_4_ nanoparticles in chloroform was mixed with 1 mL of 20 mM dodecyl trimethylammonium bromide (DTAB) in DI water via sonication for 5 min to result in the oil-in-water microemulsion. Followingly, the Fe_3_O_4_ nanoparticles were assembled through the evaporation of chloroform in solution via agitation at 25 °C for 16 h. For the stabilization of the assembled Fe_3_O_4_ nanoparticles, 4 mL of ethylene glycol containing 0.4 g of polyvinylpyrrolidone (PVP, Mw = 10,000 Da) was added to the solution, which was then gently shaken at 25 °C for 24 h and washed with ethanol via centrifugation to be dispersed in 5 mL of ethanol. To precisely adjust their size and preserve the assembled structure, PVP-stabilized Fe_3_O_4_ nanoparticle assemblies were enveloped in a silica (SiO_2_) layer to become M1 by the following procedures. First, 2.5 mL of the resultant Fe_3_O_4_ nanoparticle assembly was dispersed in 10 mL of ethanol, added with 0.5 mL of ammonia solution and 1.5 mL of DI water, then stirred for 15 min. While being stirred, the solution was supplemented with 10 μL of tetraethyl orthosilicate (TEOS) nine times with an in-between interval of 10 min and kept stirred at 25 °C for 2 h. Subsequently, the resultant M1 was washed with ethanol via centrifugation and then dispersed in 10 mL of ethanol. The M1 were consequently functionalized with amine groups for their versatile applications, in which 10 mL of M1 were added with 2 mL of (3-aminopropyl) triethoxysilane (APTES) and stirred at 25 °C for 16 h, washed with ethanol via centrifugation and then dispersed in 5 mL of DI water. The synthesized M1 exhibited a diameter of 450 nm.

To synthesize M2 with a length of 1400 nm and a diameter of 140 nm, the precursor micro-anisotropic β-FeOOH that becomes M2 upon the formation of a silica layer envelop followed by phase transformation was prepared. For their synthesis via a hydrolysis reaction, 7.5 g of iron(III) chloride hexahydrate (FeCl_3_·6H_2_O) and 70.8 μL of HCl were dissolved in 40 mL of DI water and heated at 85 °C for 16 h. The resultant precursor micro-anisotropic β-FeOOH was washed with ethanol via centrifugation and then dispersed in 10 mL of DI water. To stabilize them, the suspension was mixed with 10 mL of 2 wt% PVP in DI water for 16 h, washed with DI water via centrifugation, and then dispersed in 12 mL of DI water. To precisely adjust their size and preserve the anisotropic structure, PVP-stabilized precursor micro-anisotropic β-FeOOH were enveloped in a silica layer by the following procedures. First, 1 mL of the resultant precursor micro-anisotropic β-FeOOH was dispersed in 25 mL of ethanol, added with 1.25 mL of ammonia solution and 3.75 mL of DI water, then stirred for 15 min. While being stirred, the solution was supplemented with 10 μL of TEOS three times with an in-between interval of 15 min and kept stirred at 25 °C for 2 h. Subsequently, the resultant M2 was washed with ethanol via centrifugation and then dispersed in 3 mL of DI water. For their magnetic manipulability, the silica layer-covered precursor micro-anisotropic β-FeOOH was subjected to thermal phase transformation to become the magnetic silica layer-enveloped micro-anisotropic Fe_3_O_4_ (M2). To this end, 3 mL of the silica layer-enveloped precursor micro-anisotropic β-FeOOH was dispersed in 20 mL of triethylene glycol (TEG) and then heated at 340 °C for 2 h under a nitrogen atmosphere. Subsequently, the resultant M2 was washed with ethanol via centrifugation and then dispersed in 6 mL of ethanol. The M2 was consequently functionalized with amine groups for their versatile applications, in which 5 mL of M2 was first dispersed in 30 mL of ethanol, added with 0.5 mL of APTES, stirred at 25 °C for 16 h, washed with ethanol via centrifugation and then dispersed in 5 mL of DI water. The synthesized M2 exhibited a length of 1400 nm and a diameter of 140 nm.

### 2.3 Tailoring the multi-scale anisotropy of hierarchical nanostructure

For the precise tailoring of multi-scale ligand anisotropy presented to the cells at the constant densities, the concentrations of N1 vs. N2 and of M1 vs. M2 were modulated. This strategy was applied when coating the ligand-couplable nanoscale anisotropy-tailored Au (N1 or N2) on the magnetically manipulable microscale anisotropy-tailored Fe_3_O_4_ (M1 or M2) at the constant density among the groups. Specifically, the concentration of N2 was four times higher than that of N1 and the concentration of M2 was three times higher than that of M1 to yield similar numbers of N1 and N2 on the surface of each M1 or M2. To this end, 1 mL of amine-functionalized M1 or M2 was added dropwise under sonication to 4 mL of N1 or N2, subsequently followed by sonication for 1 min and then shaking at 25 °C for 1 h. The N1 or N2 was stably coupled on the surfaces of M1 or M2 via gold-amine bonds to form multi-scale anisotropy-tailored hierarchical nanostructures. Depending on their combinations, various composites of “M1+N1”, “M1+N2”, “M2+N1”, and “M2+N2” groups were created. The resultant 5 mL solution of hierarchical nanostructures was subjected to magnetic separation by using a permanent magnet (285 mT) for 1 h to remove the supernatants, which were then dispersed in 5 mL of 0.2 mM PVP (Mw = 55,000 Da) in DI water and shaken at 25 °C for 3 h. The PVP-stabilized hierarchical nanostructures were washed with DI water via centrifugation and finally dispersed in 5 mL of DI water.

## 3 Results and discussion

### 3.1 Independent tailoring of the hierarchical ligand anisotropy

The hierarchical ligand anisotropy was independently modulated when designing hierarchical nanostructures exhibiting ligand anisotropy at the multi-scale but at the same ligand densities. Typically, the development of hierarchical structures with two anisotropic materials has been limited as their surface curvature (inversely proportional to the diameter) [79] generally increases with increasing anisotropy at the fixed surface area, which results in an insufficient contact area between the two curved surfaces to stably support their weight and hence the coupled state. However, two different anisotropic materials with a considerable size difference (e.g., one in the smaller nanoscale and the other in the larger microscale) could provide a sufficient contact area and hence strong coupling leading to the hierarchical structure. Hence, we separately synthesized N-Au and M − Fe_3_O_4_ particles with tailored anisotropy and then coupled them to create remotely manipulable hierarchical ligand nanostructures (Scheme 1a).

Before their synthesis, the target dimensions of each nanoscale anisotropy-tailored (isotropic or anisotropic) Au (N1 or N2) and microscale anisotropy-tailored (isotropic or anisotropic) Fe_3_O_4_ (M1 or M2) were calculated for their independent anisotropy tailoring at equivalent surface areas. The morphologies of each isotropic or anisotropic material resemble those of the sphere or the rod, respectively, with both ends terminated by hemispheres. Therefore, their surface areas were calculated as 4π(D/2)^2^ for isotropic materials [diameter (D)] vs. 4π(D/2)^2^ + [(L - D) × 2π(D/2)] for anisotropic materials [length (L) and diameter (D)] (Figs. S1 and S2). The desired nanoscale dimensions were set as 45 nm (diameter) for N1 vs. 18 nm (diameter) and 118 nm (length) for N2, and the microscale dimensions were set as 450 nm (diameter) for M1 vs. 140 nm (diameter) and 1400 nm (length) for M2. Such a difference of one to two orders of magnitude in the degree of expected anisotropy (aspect ratio) between N2 and M2 was devised to ensure their stable coupling while achieving similar anisotropies at such different scales.

**Fig. 1:**
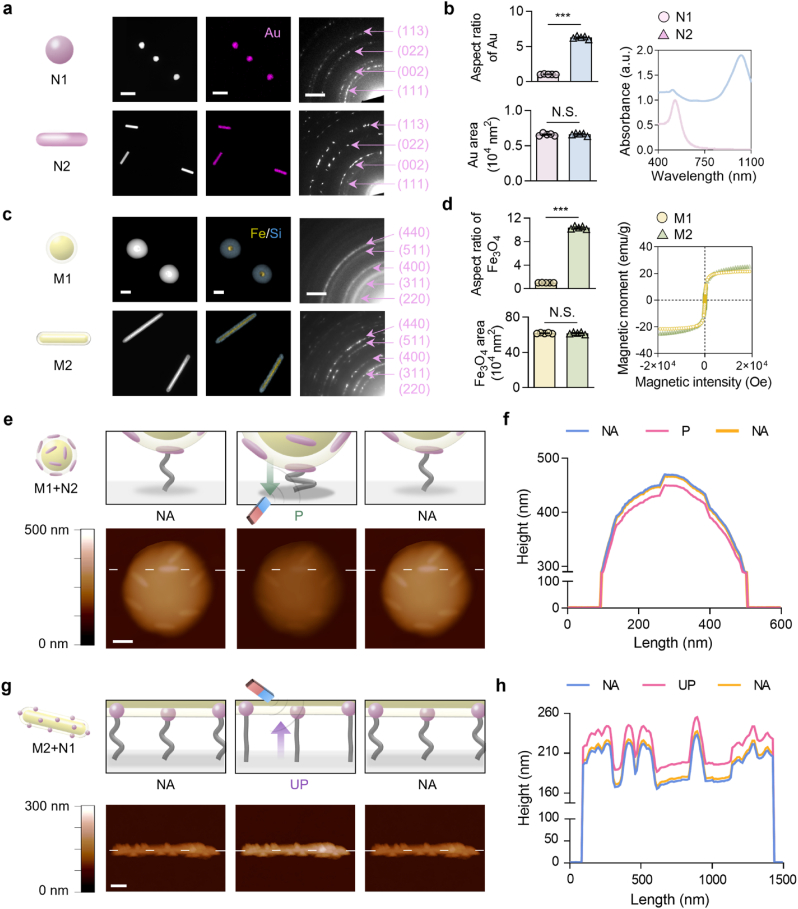
Hierarchical nanostructures exhibiting multi-scale ligand anisotropy are cyclically reversible via magnetic manipulation. (a) The structural analysis of nano-isotropic or anisotropic Au (N1 or N2) with high-angle annular dark-field scanning transmission electron microscopy (HAADF-STEM), energy-dispersive X-ray spectroscopy (EDS) mapping (Au from N1 or N2) images, and selected area diffraction (SAED) pattern [scale bars: 200 nm (HAADF-STEM) and 2 nm^−1^ (SAED)] with (b) the quantifications of their aspect ratios and surface areas along with the absorbance. (c) The structural analysis of micro-isotropic or anisotropic Fe_3_O_4_ (M1 or M2) with HAADF-STEM, EDS mapping (Fe from the Fe_3_O_4_ core and Si from the silica layer envelop) images, and an SAED pattern [scale bars: 200 nm (HAADF-STEM) and 2 nm^−1^ (SAED)] with (d) the quantifications of their aspect ratios and surface areas along with magnetic characteristics verified by vibrating sample magnetometry (VSM). (e) Atomic force microscopy (AFM) images and (f) following linear height profile of reversible downward manipulation of the “M1+N2” group by placing [“Pressed (P)”] or not placing [“Non-affected (NA)”] the magnet under the substrate. The gradient color bar indicates their height (scale bar in the image: 100 nm). (g) AFM images and (h) following linear height profile of reversible upward manipulation of the “M2+N1” group by placing [“Unpressed (UP)”] or not placing (“NA”) the magnet over the substrate. The gradient color bar indicates their height (scale bar in the image: 200 nm). Data are exhibited as the mean ± standard error (n = 4). Asterisks assigned to the range of p values (∗∗∗: p < 0.001) represent statistically significant differences. N.S. signifies that there is no statistically significant difference among the compared groups.

For the independent tailoring of nanoscale ligand anisotropy exhibiting similar surface areas, the N1 sphere and N2 rod that present anisotropy-controlled ligands were separately prepared by varying the surfactant type, surfactant amount, and precursor amount. For the anisotropy-controlled synthesis of N-Au, seed-mediated growth in the absence (isotropic sphere) or presence (anisotropic rod) of surfactants was employed. The analysis of N1 by using transmission electron microscopy (TEM), high-resolution TEM (HR-TEM), and fast Fourier transform (FFT) demonstrated their homogeneous isotropic shape with crystalline lattice planes specific to Au while the dynamic light scattering (DLS) measurement revealed their nanoscale hydrodynamic diameter as 44.5 ± 7.3 nm (Figs. S3a–c). Such Au specificity was also observed in the analysis of N2 that exhibited a homogeneous anisotropic shape with a nanoscale length of 121.0 ± 3.7 nm and a diameter of 22.6 ± 3.1 nm (Figs. S4a–c). Moreover, zeta potential measurements for the changes in the surface charge of N2 (serially capped with cetrimonium bromide, polystyrene sulfonate, and finally citrate) confirmed that ligand exchange was successful during its synthesis (Fig. S4d). High-angle annular dark-field scanning TEM (HAADF-STEM), energy-dispersive X-ray spectroscopy (EDS), and selective area electron diffraction (SAED) analysis of both N1 and N2 confirmed the elemental conformation of Au with respective crystalline planes (Fig. 1a). Furthermore, mathematical calculations of their aspect ratio and surface area confirmed the independent tailoring of nanoscale anisotropy at equivalent surface areas for their significantly different aspect ratios of 1 and 6.6 (approximately 7), which exhibited a red shift in the absorption peaks with increasing anisotropy in the UV–Vis spectrophotometer analysis (Fig. 1b and Fig. S1).

For the independent tailoring of microscale ligand anisotropy at equivalent surface areas, magnetically manipulable M1 sphere and M2 rod were separately prepared by varying the nanocore type and shell dimension with phase transformation. M2 was synthesized by assembling Fe_3_O_4_ nanoparticles, enveloping them with a silica layer, and finally phase transforming the precursor (micro-anisotropic β-FeOOH) enveloped by the silica layer. The examination of M1 with TEM, HR-TEM, FFT, X-ray diffraction (XRD), and DLS revealed their homogeneous isotropic shape, atomic planes specific to the crystalline Fe_3_O_4_, and a microscale hydrodynamic diameter of 456.0 ± 61.4 nm (Figs. S5a–d). Likewise, the Fe_3_O_4_ specificity was also observed in the analysis of M1, which exhibited a homogeneous anisotropic shape with a microscale length of 1415.8 ± 28.0 nm and a diameter of 140.4 ± 9.0 nm (Figs. S6a–d). The morphological characterization of the N-Au and M − Fe_3_O_4_ particles are summarized in Table S1. HAADF-STEM, EDS, and SAED analysis of both M1 and M2 confirmed the elemental conformation of Fe (in the Fe_3_O_4_ core) and Si (in the silica layer envelop) with respective crystalline planes for Fe_3_O_4_ (Fig. 1c). Furthermore, mathematical calculations of their aspect ratios and surface area confirmed the independent tailoring of microscale anisotropy at equivalent surface areas with their significantly different aspect ratios of 1 and 10, which both exhibited magnetic reversibility with negligible coercivity in vibrating sample magnetometry analysis (Fig. 1d and Fig. S2).

Followingly, independent tailoring of hierarchical nanostructures exhibiting anisotropy at the multi-scale at constant ligand densities was achieved by modulating the concentrations of both N1 vs. N2 and M1 vs. M2. This strategic process yielded N1 or N2 at constant density coated on the amine-functionalized surface of M1 or M2 at constant density through Au-amine bonds. The combinations of different nanoscale anisotropies (i.e., N1 and N2) with microscale anisotropies (i.e., M1 and M2) resulted in four different hierarchical nanostructures (M1+N1, M1+N2, M2+N1, and M2+N2) that retained the characteristic specificity of the elemental conformation, atomic planes, and absorbance of each N-Au and M − Fe_3_O_4_ (Figs. S7, S8, and S9). Moreover, all the hierarchical nanostructures displayed magnetic reversibility even after the coupling of non-magnetic N-Au on the surface of magnetic M − Fe_3_O_4_ (Fig. S10).

Subsequently, the magnetically manipulable hierarchical nanostructures were independently coupled on the substrates at an equivalent density among various groups by using elastic polymer (PEG) linkers. The Au surfaces on Fe_3_O_4_ were then decorated with cell-adhesive RGD ligands (Fig. S11). To this end, N-Au of the hierarchical nanostructures were first PEGylated via an Au-thiol bond, after which were coupled onto the amine-functionalized substrate surfaces. The linkers that did not react with the substrate surface were then coupled with RGD ligands and the remaining surface was passivated to avoid non-specific cell adhesion. The serial coupling of the hierarchical nanostructures with PEG linkers and ligands was confirmed by the changes in the characteristic chemical bonds after PVP-stabilization, PEG linker-coupling, and the ligand-coupling via Fourier transform infrared spectroscopy (Fig. S12). Scanning electron microscopy (SEM) images of the substrates with hierarchical ligand nanostructures revealed the homogeneous distribution of independently tailored hierarchical ligand anisotropy at multi-scale, which was evidenced by similar total Au and Fe_3_O_4_ areas, densities of substrate-coupled Fe_3_O_4_ per μm^2^, and the total liganded area per each hierarchical nanostructure (Fig. S13a,b and Fig. S14). Moreover, the number of N-Au particles per M − Fe_3_O_4_ as well as RGD ligand per N-Au were constant across all groups (Table S2).

### 3.2 Ligand anisotropy at the microscale is dominant over that at the nanoscale in activating macrophage adhesion

After the successful conjugation of hierarchical ligand nanostructures on substrates, we wondered how each nanoscale and microscale ligand anisotropy contributes to the recruitment and binding of cell integrins that regulate cell adhesion. To this end, macrophages were selected as the model cell type owing to their relatively small microscale size [80], thereby allowing efficient regulation by each hierarchical ligand nanostructure, whose response can be analyzed by examining the adhesion and phenotypic polarization [48]. For such assessment, macrophages were seeded (only at the initial time point) and cultured on the substrates presenting multi-scale ligand anisotropy (“M1+N1”, “M1+N2”, “M2+N1”, and “M2+N2” groups) for 24 h. Importantly, we decoupled the multi-scale (nanoscale vs. microscale) for isotropy vs. anisotropy in the ligand geometry.

The immunofluorescence images and crystal violet-stained images (cell attachment assay) of macrophages adhered to the group exhibiting ligands with both microscale and nanoscale “isotropy” (“M1+N1”) showed minimal expression of integrin β1 along with vinculin and F-actin in low levels of the planar density, size, and aspect ratio of the cells (Figs. S15a, S16a,b, S17a,b, and S18). As the ligands in nanoscale “isotropy” are changed to nanoscale “anisotropy” while maintaining microscale ligand isotropy, cellular adhesion was slightly promoted but without statistically significant differences. In stark contrast, when the ligands in microscale “isotropy” are changed to microscale “anisotropy”, cellular adhesion is highly stimulated in the groups of both nanoscale “isotropy” and “anisotropy” (“M2+N1” and “M2+N2” groups) compared with the groups of microscale isotropy (both “M1+N1” and “M1+N2” groups) (Figs. S15a, S16a,b, S17a,b, and S18). Notably, the expression of integrin β1, vinculin, and F-actin, as well as the planar density, size, and aspect ratio of cells were all markedly promoted in the group of microscale anisotropy but with nanoscale isotropy (“M2+N1”) compared to the group of nanoscale anisotropy but with microscale isotropy (“M1+N2”). Such trend was corroborated with not only the recruitment of integrins but also their activation (Figs. S19a and b). This proves that microscale ligand anisotropy (despite nanoscale ligand isotropy) dominates over nanoscale ligand anisotropy in the recruitment of integrins and adhesion of macrophages. Immunogold labeling and SEM imaging corroborated this trend that the “M1+N2” and “M2+N1” groups showed 3.7 ± 0.6 and 5.3 ± 0.6 integrin-labeling Au nanoparticles per hierarchical nanostructures on the adhered macrophages, respectively, in the “Non-affected” (NA) state (Figs. S15b–e).

In this configuration, the ligand anisotropy at each scale was independently tailored at constant surface areas and it was recently reported that integrin molecules can bridge ligands populated in a spacing of tens of nm [47,63,81]. Therefore, similar levels of cell adhesion in the groups of microscale isotropy (both “M1+N1” and “M1+N2” groups) could be attributed to integrin molecules readily bridging both adjacent nanoscale Au spheres (N1) and adjacent nanoscale Au rods (N2) to comparable degrees, each of which populated in a spacing of tens of nm. This interpretation could also explain the similar degrees of cell adhesion in the groups of microscale anisotropy (both “M2+N1” and “M2+N2” groups). By contrast, the significantly higher cell adhesion observed in the “M2+N1” group compared to the “M1+N2” group suggests that liganded integrin clusters with diameters in the range of 0.1–1.5 μm can bridge adjacent microscale Fe_3_O_4_ rods populated in smaller spacing, possibly by recruiting unliganded integrins, more efficiently than adjacent microscale Fe_3_O_4_ spheres populated in a larger spacing [63]. These outcomes collectively reveal that hierarchical ligand nanostructures exhibiting multi-scale ligand anisotropy can help to achieve and decipher multimodal scale-specific regulation of cells.

To ascertain the multi-scale ligand anisotropy-specific effect on macrophage regulation, sequential experiments were performed without RGD ligands. First, ligand anisotropy tailoring of M − Fe_3_O_4_ alone did not promote the adhesion of macrophages (Figs. S20a and b). Furthermore, additional tailoring of nanoscale anisotropy via the conjugation of N-Au on the surfaces of M − Fe_3_O_4_ was also ineffective, thereby verifying the necessity of RGD ligands for the regulation of macrophage (Figs. S21a and b).

### 3.3 Remote manipulation cyclically switches scale-specific-ligand-anisotropy-regulated macrophage adhesion

We then pondered whether the remote manipulation of hierarchical nanostructures could modulate their scale-specific ligand anisotropy-based regulation of macrophage integrin recruitment and adhesion. To this end, hierarchical ligand nanostructures exhibiting disparate ligand anisotropies at the multi-scale (both “M1+N2” and “M2+N1” groups) were chosen to examine the effect of the axial manipulation of scale-specific ligand anisotropy on macrophages. For such assessment, macrophages were cultured on substrates with or without [“Non-affected” (NA)] a permanent magnet placed over or under the substrates that direct the upward (substrate-distant) or downward (substrate-proximate) movement of the nanostructures to “Unpress” (UP) or “Press” (P) the substrates, respectively. The immunofluorescence and SEM images of macrophages adhered to the remotely manipulated “M1+N2” group revealed the stimulation of macrophage adhesion in their “P” state compared to the “NA” state with significantly higher planar cell density and integrin-labeling Au nanoparticles per hierarchical nanostructure on macrophages, while their “UP” state did not exhibit significant changes (Fig. S15b,c and Fig. S22a). By contrast, the “UP” manipulation of the “M2+N1” group exhibited significant suppression of macrophage adhesion with substantially reduced planar cell density and integrin-labeling Au nanoparticles, whereas their “P” state was similar to their “NA” state (Fig. S15d,e and Fig. S22b). Strikingly, the “P” state of the “M1+N2” group promoted macrophage adhesion similar to the “M2+N1” group in the “NA” state whereas the “UP” state of the “M2+N1” group suppressed it comparable to the “M1+N2” group in the “NA” state, thereby suggesting the switching of a scale-specific ligand anisotropy effect via remote axial manipulation (Figs. S23a and b).

Recent reports have highlighted the inhibitory effect on cell adhesion via the membrane bending energy resulting from highly curved surfaces [57,[82], [83], [84]]. Therefore, such regulation could be attributed to the bending of cells when adhering to the ligands on the hierarchical nanostructures, which increases or decreases by the “UP” or “P” manipulation of hierarchical nanostructures, respectively. Furthermore, the distance between the hierarchical nanostructures and the substrate during magnetic manipulation, which has been reported to influence the development of cellular traction force and mechanosensing during adhesion, could have also contributed to macrophage regulation [[85], [86], [87]]. Such an effect could have also contributed to the macrophage regulation of hierarchical nanostructures tailored with multi-scale anisotropy, where curvature differences arise due to height variations resulting from shape anisotropy. The insignificant difference between the “UP” and “NA” states of the “M1+N2” group and between the “P” and “NA” states of the “M2+N1” group suggests that inhibitory and stimulatory effects on each cell adhesion were already saturated, respectively, and hence could not be further regulated via their axial manipulation. Thus, this study demonstrates for the first time scale-specific regulation of ligand anisotropy for dynamic cell regulation, which has not been demonstrated with the static modulation of anisotropy in the geometry of tissues [65] or material shapes [66].

Consequently, we speculated whether such scale-switchable macrophage regulation via remote manipulation of hierarchical ligand nanostructures could be cyclically reversed. First, *in situ* atomic force microscopy (AFM) analysis of the “M1+N2” group revealed the serial measurements of their peak heights of 470.2 nm (“NA”), 450.2 nm (“P”), and 467.9 nm (“NA”) upon their reversible downward (substrate-proximate) manipulation, thereby proving high reversibility (Fig. 1e and f). To examine whether such switching leads to reversible regulation of cell adhesion, macrophages were cultured on the “M1+N2” group with downward magnet application either switched or maintained every 12 h (after 24 h of culturing) up to 48 h (the “NA-NA-NA”, “NA-P-NA”, “P-NA-P”, and “P-P-P” states). The resultant immunofluorescence images revealed reversibly regulatable macrophage adhesion with elevated integrin expression and increased planar density, size, and aspect ratio only right after being cultured on the “P” state, all of which decreased right after being cultured on the “NA” state (Fig. S24a,b and Fig. S25). For the reversible manipulation of the “M2+N1” group, upward (substrate-distant) magnetic application was either switched or maintained every 12 h (after 24 h of culturing) up to 48 h (the “NA-NA-NA”, “NA-UP-NA”, “UP-NA-UP”, and “UP-UP-UP” states). The *in situ* AFM analysis of the “M2+N1” group proved their high reversibility as evidenced by serially measured peak heights of 238.5 nm (“NA”), 256.3 nm (“UP”), and 242.0 ± 0.0 nm (“NA”) upon reversible upward manipulation (Fig. 1g and h). Likewise, the reversible manipulation of the “M2+N1” group led to the reversible regulation of macrophages, in which their adhesion was promoted only right after being cultured on the “NA” state while being hindered right after being cultured on the “UP” state (Fig. S26a,b and Fig. S27).

In contrast, such remote control of hierarchical ligand nanostructures exhibiting either dual nanoscale/microscale isotropy or dual nanoscale/microscale anisotropy only inflicted insignificant switchability of scale-specific effects on macrophage regulation. Both “UP” and “P” states of the “M1+N1” group, which originally inhibited macrophage adhesion in the “NA” state, showed comparable low levels of cell adhesion with only a slight promotion in the “P” state without statistically significant differences (Figs. S28a and b). Similarly, both axially manipulated states of the “M2+N2” group that originally promoted macrophage adhesion in the “NA” state exhibited a similar trend, with a trivial decrease in the “UP” state without statistically significant differences (Figs. S29a and b). This trend could be attributed to dual ligand isotropy or anisotropy effects that exerted extremely inhibitory or stimulatory effects, respectively, and were thus difficult to be modulated.

### 3.4 Remote manipulation of hierarchical ligand anisotropy switches adhesion-mediated macrophage polarization

The regulation of macrophage adhesion by the multi-scale ligand anisotropy-dependent recruitment and clustering of integrin molecules can further modulate the phenotypic polarization of the macrophages (pro-regenerative M2 vs. pro-inflammatory M1 polarization), which involves Rho A and Rho-associated kinase 2 (ROCK2) signaling pathways [51,88,89]. Typically, firmly adhered macrophages expressing a high cell aspect ratio (elongated shape) lead to their pro-regenerative M2 polarization while those with relatively suppressed adhesion expressing a low cell aspect ratio (round shape) lead to their pro-inflammatory M1 polarization [49,90]. Consequently, the pro-regenerative M2 or pro-inflammatory M1 phenotypic polarization of macrophages under remote switching of the scale-specific effect on macrophage adhesion was investigated after 36 h of culturing the substrates with hierarchical nanostructures (both “M1+N2” or “M2+N1” groups) under pro-regenerative M2 or pro-inflammatory M1 medium with (“P” or “UP” states, respectively) or without (“NA” state) axial remote control.

**Fig. 2:**
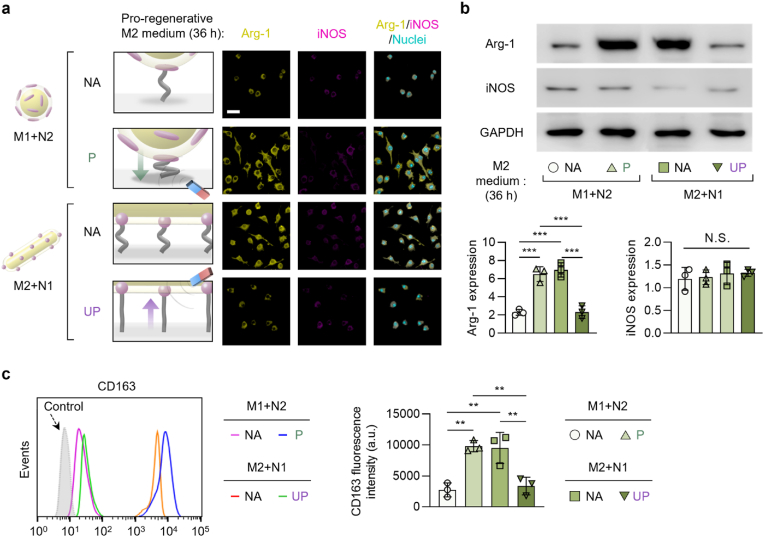
Axial manipulation of multi-scale ligand anisotropy switches the pro-regenerative macrophage polarization. (a) Fluorescently immuno-stained images of Arg-1 (pro-regenerative M2 marker) and iNOS (pro-inflammatory M1 marker) and their overlay with nuclei (scale bars: 20 μm) and (b) the western blotting images with quantitative analysis of Arg-1 and iNOS proteins of the adherent macrophages after 36 h of culturing either on the micro-isotropic + nano-anisotropic (“M1+N2”) group in the “non-affected (NA)” or “pressed (P)” states, or on the micro-anisotropic + nano-isotropic (“M2+N1”) group in the “NA” or “unpressed (UP)” states in pro-regenerative M2 medium. The protein expression levels in the western blotting images were presented after normalization to GAPDH expression. (c) Flow cytometry of the CD163 (pro-regenerative M2 marker) expression with computed characterization of their respective fluorescence intensities. The magnet was not placed in the “NA” state or placed either under or over the substrates to induce the “P” or “UP” state, respectively. Data are exhibited as the mean ± standard error (n = 10). Asterisks assigned to the range of p values (∗∗: p < 0.01; ∗∗∗: p < 0.001) represent statistically significant differences. N.S. signifies that there is no statistically significant difference among the compared groups.

**Fig. 3:**
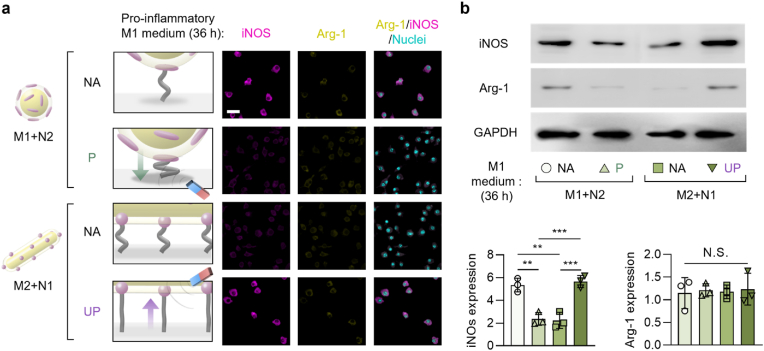
Axial manipulation of multi-scale ligand anisotropy switches the pro-inflammatory macrophage polarization. (a) Fluorescently immuno-stained images of iNOS (pro-inflammatory M1 marker) and Arg-1 (pro-regenerative M2 marker) and their overlay with nuclei (scale bars: 20 μm) and (b) the western blotting images with quantitative analysis of iNOS and Arg-1 proteins of the adherent macrophages after 36 h of culturing either on the micro-isotropic + nano-anisotropic (“M1+N2”) group in the “non-affected (NA)” or “pressed (P)” states, or on the micro-anisotropic + nano-isotropic (“M2+N1”) group in the “NA” or “unpressed (UP)” states in pro-inflammatory M1 medium. The protein expression levels in the western blotting images were presented after normalization to GAPDH expression. The magnet was not placed in the “NA” state or placed either under or over the substrates to induce the “P” or “UP” state, respectively. Data are exhibited as the mean ± standard error (n = 10). Asterisks assigned to the range of p values (∗∗: p < 0.01; ∗∗∗: p < 0.001) represent statistically significant differences. N.S. signifies that there is no statistically significant difference among the compared groups.

The immunofluorescence images, western blotting, and flow cytometry analysis of macrophages revealed that the scale-specific remote manipulation of hierarchical ligand nanostructures that regulated the adhesion of macrophages correspondingly modulated their phenotypic polarization (Fig. 2, Fig. 3a,b). After being cultured in a pro-regenerative M2 medium, macrophages adhered to the “M2+N1” group in the “NA” state, which promotes cell adhesion, exhibited the highest pro-regenerative Arg-1 expression that was drastically suppressed in their “UP” state. The lowest expression of Arg-1 in macrophages cultured in a pro-regenerative M2 medium on the “M1+N2” group in the “NA” state, which suppresses cell adhesion, was significantly elevated in their “P" state to a degree comparable to those examined in the “M2+N1” group in the “NA” state. Similar to Arg-1, pro-regenerative CD163 expression was high for the “M2+N1” group in “NA” state, which was suppressed in “UP” state, whereas the “M1+N2” group showed low CD163 expression in “NA” state, which was elevated in “P” state (Fig. 2a–c). Conversely, the highest pro-inflammatory iNOS expression of macrophages cultured in a pro-inflammatory M1 medium on the groups, that suppress cell adhesion (the “M1+N2” group in the “NA” state and the “M2+N1” group in the “UP” state), was reversed to promote cell adhesion on the groups upon axial manipulation (the “M1+N2” group in the “P” state and the “M2+N1” group in the “NA” state) (Fig. 3a and b). Collectively, these results prove that the axial manipulation of hierarchical nanostructures that can switch the scale-specific ligand anisotropy effect on macrophage adhesion can also translate into the regulation of their phenotypic polarization.

**Fig. 4:**
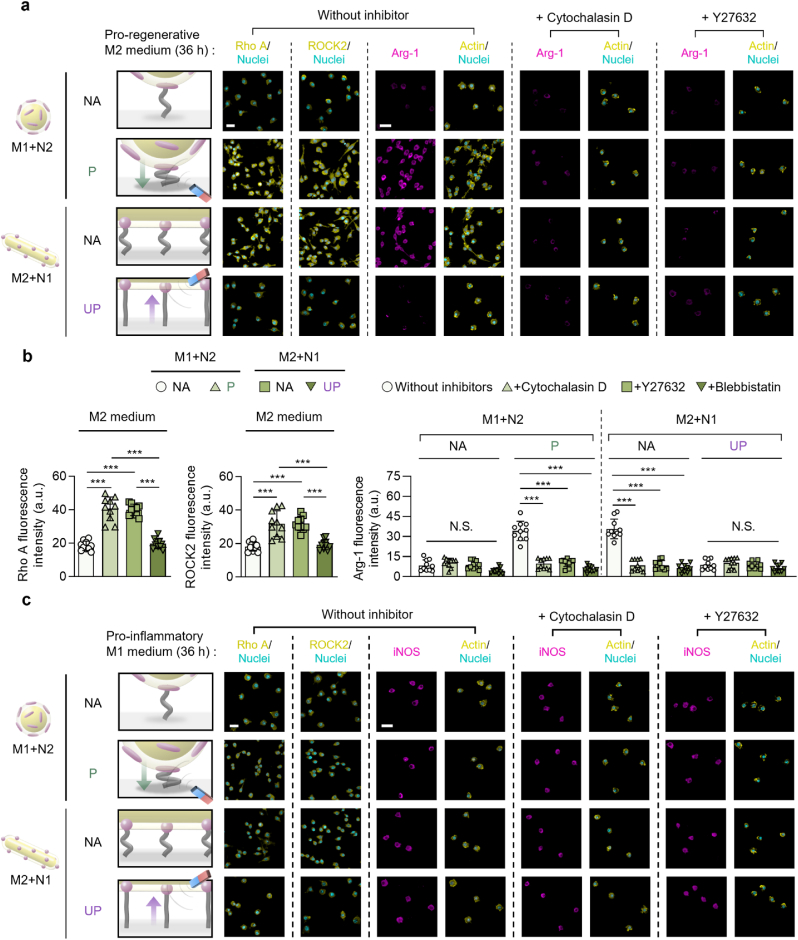
Dynamic adhesion-regulated macrophage polarization is switched by axial manipulation of multi-scale ligand anisotropy. (a) Fluorescently immuno-stained images of Rho A or ROCK2 with nuclei and Arg-1 with F-actin/nuclei of adherent macrophages after 36 h of culturing on the micro-isotropic + nano-anisotropic (“M1+N2”) group in the “non-affected (NA)” or “pressed (P)” state or on the micro-anisotropic + nano-isotropic (“M2+N1”) group in the “NA” or “unpressed (UP)” state in pro-regenerative M2 medium with or without the supplementation of specific inhibitors for each actin polymerization or Rho-associated protein kinase (cytochalasin D or Y27632, respectively) (scale bars: 20 μm). (b) Following calculations of Rho A, ROCK2, and Arg-1 fluorescence intensities including the fluorescently immuno-stained images in Fig. S30. (c) Fluorescently immuno-stained images of Rho A or ROCK2 with nuclei and iNOS with F-actin/nuclei of the adherent macrophages after 36 h of culturing on the “M1+N2” group in the “NA” or “P” state or on the “M2+N1” group in the “NA” or “UP” state in pro-inflammatory M1 medium with or without the supplementation of one of the specific inhibitors for actin polymerization or Rho-associated protein kinase (cytochalasin D or Y27632, respectively) (scale bar: 20 μm). The magnet was not placed in the “NA” state or placed either under or over the substrates to induce the “P” or “UP” state, respectively. Data are exhibited as the mean ± standard error (n = 10). Asterisks assigned to the range of p values (∗∗∗: p < 0.001) represent statistically significant differences. N.S. signifies that there is no statistically significant difference among the compared groups.

To further delve into the adhesion-mediated regulation of macrophage polarization via the axial manipulation of hierarchical ligand nanostructures, we investigated the molecular mechanisms of phenotypic polarization-regulated macrophages. The expression intensities of Rho A, ROCK2, and Arg-1, of which the former activates the latter, were shown to be upregulated in the groups that exhibited higher adhesion and pro-regenerative M2 polarization (the “M2+N1” group in the “NA” state and the “M1+N2” group in the “P” state) after culturing in a pro-regenerative M2 medium (Fig. 4a and b). The analyzation of macrophages after inhibiting specific adhesion-related molecules associated with actin polymerization, ROCK, or myosin II (via cytochalasin D, Y27632, or blebbistatin, respectively) in a pro-regenerativeM2 medium proved the macrophage polarization to be dependent on their adhesion regulation (Fig. 4a and Fig. S30). Notably, both Arg-1 expression and adhesion were suppressed in the groups that originally promoted macrophage adhesion and pro-regenerative M2 polarization (both “M1+N2” group in the “P” state and “M2+N1” group in the “NA” state) (Fig. 4b and Figs. S30 and S31). Upon macrophage culturing in a pro-inflammatory M1 medium, the fluorescence intensities of both Rho A and ROCK2 were not promoted as much in the pro-regenerative M2 polarization-promoting groups (Fig. 4c and Fig. S32). Moreover, the inhibition of adhesion-related molecules during macrophage culture in a pro-inflammatory M1 medium induced high expression of iNOS on the groups, which originally supported cell adhesion and suppressed pro-inflammatory M1 polarization, thereby leading to pro-inflammatory M1 polarization (both “M1+N2” group in the “NA” state and “M2+N1” group in the “UP” state) (Fig. 4c and Fig. S33). Conclusively, remote manipulation of hierarchical nanostructures can reverse the scale-specific ligand anisotropy effect on the regulation of macrophage polarization, which is governed by adhesion-related molecules.

### 3.5 Remote manipulation of hierarchical ligand anisotropy switches host macrophage regulation In vivo

Having confirmed the effect of scale-specific ligand anisotropy upon axial manipulation for the regulation of macrophages *in vitro*, we next pondered whether such a striking phenomenon can be translated *in vivo* to regulate host cells (e.g., host macrophages and host neutrophils). To this end, a robust silicon substrate was chosen to present different multi-scale ligand anisotropies (both “M1+N2” and “M2+N1” groups) that was subcutaneously implanted in the dorsal regions of mice (Fig. S34). To reduce the effect of the predominant inflammatory host response right after implantation, an anti-inflammatory mixture of interleukin (e.g., IL-4 and IL-13) was injected on the implanted substrate surface. The short-term host response at 24 h post-implantation was examined as this governs the long-term host response (e.g., inflammation and tissue regeneration). For the axial manipulation to induce the “UP” or “P” state of hierarchical nanostructures, the permanent magnet (285 mT) was attached either to the backs or abdomens of mice to direct upward or downward nanostructure movement, respectively.

Consistent with previous validations [91,92], high biocompatibility of hierarchical ligand nanostructures (composed of Au and Fe_3_O_4_ nanostructures) and magnetic field were confirmed via the histological analysis of a localized area of subcutaneous tissue and systemic areas of the heart, liver, and kidney of the mice at 0 and 7 d post-implantation through hematoxylin and eosin (H&E) staining (Fig. S35). The SEM examinations of the substrates at 0 and 7 d post-implantation revealed a consistent density of both nanoscale Au and microscale Fe_3_O_4_ without any signs of degradation, thereby proving the maintenance of their stable coupling in the host microenvironment (Figs. S36a and b).

**Fig. 5:**
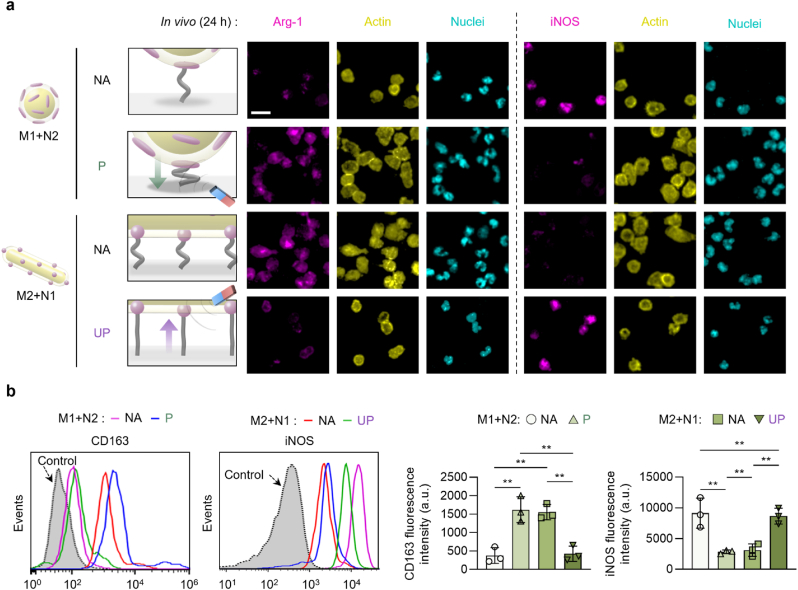
Axial manipulation of multi-scale ligand anisotropy dynamically switches host macrophage adhesion-dependent polarization *in vivo*. (a) Fluorescently immuno-stained images of Arg-1 or iNOS with F-actin and nuclei of adherent host macrophages after 24 h of implantation (scale bars: 20 μm). (b) Flow cytometry of the CD163 (pro-regenerative M2 marker) and iNOS (pro-inflammatory M1 marker) expression with computed characterization of their respective fluorescence intensities. The permanent magnet (285 mT) was not attached in the “NA” state or attached either to the abdomens or backs of the mice to induce the “P” or “UP” states, respectively. Data are exhibited as the mean ± standard error (n = 10). Asterisks assigned to the range of p values (∗∗: p < 0.01) represent statistically significant differences.

The immunofluorescence images and flow cytometry analysis of host macrophages adhered on the implanted substrates at 24 h post-implantation revealed the parallel regulation of host macrophages with those examined *in vitro* (Fig. 5a and b and Fig. S37). The dominant effect of ligand anisotropy at the microscale over the nanoscale in promoting the adhesion and pro-regenerative M2 polarization of host macrophages could be reversed via remote axial manipulation of hierarchical nanostructures in the mice. After implantation, similarly promoted adhesion of host macrophages with high expression of pro-regenerative Arg-1 and CD163 was examined on the “M1+N2” group in the “P” state and on the “M2+N1” group in the “NA” state while reversing trends with high expression of pro-inflammatory iNOS were observed on the “M1+N2” group in the “NA” state and on the “M2+N1” group in the “UP” state (Fig. 5a,b and Fig. S37). Furthermore, such regulation of host cell adhesion was further verified via the corroborative adhesive trend of host neutrophils that are positive for NIMP-R14 during the short-term host response (Figs. S38a and b). Conclusively, these results not only propose novel parameters of scale-specific ligand anisotropy for the regulation of host cells but also provide potential prospects for use in *in vivo* applications that govern long-term inflammation and tissue repair.

## 4 Conclusions

In conclusion, we synthesized remotely manipulable hierarchical ligand nanostructures exhibiting multi-scale ligand anisotropy by combining nanoscale Au (comparable to integrin molecule-scale) and microscale Fe_3_O_4_ (comparable to integrin cluster-scale), which could be remotely manipulated via a magnetic field at constant ligand density across the groups for host macrophage regulation *in vivo*. In their “Non-affected” (NA) state, the increase of ligand anisotropy in hierarchical nanostructures, which sequentially promotes macrophage integrin recruitment, adhesion, and pro-regenerative M2 polarization, involving Rho A and ROCK 2 signaling pathways, was dominant at the microscale over the nanoscale. Remote magnetic manipulation of the hierarchical nanostructures can reverse such scale-specific ligand anisotropy effect on host macrophage regulation. The substate-proximate manipulation of hierarchical nanostructures exhibiting microscale isotropy and nanoscale anisotropy (“M1+N2”) into the “Pressed” (P) state promoted the adhesion and pro-regenerative M2 polarization of macrophages compared to their “NA” state. Conversely, the substate-distant manipulation of hierarchical nanostructures exhibiting microscale anisotropy and nanoscale isotropy (“M2+N1”) into the “Unpressed” (UP) state hindered macrophage adhesion, thereby promoting pro-inflammatory M1 polarization compared to their “NA” state. Unlimited tuning of the scale, anisotropy, and shape of both liganded nanoscale materials and magnetic microscale materials as well as development into a 3D structure can help to mimic the native environment that regulates host macrophage polarization to advance immunoengineering strategy *in vivo*.

## CRediT authorship contribution statement

**Kanghyeon Kim:** Writing – original draft, Investigation, Formal analysis, Data curation. **Sunhong Min:** Writing – original draft, Investigation, Formal analysis, Data curation. **Ramar Thangam:** Writing – original draft, Resources, Methodology, Formal analysis. **Kyong-Ryol Tag:** Visualization, Investigation, Formal analysis, Data curation. **Hyun-Jeong Lee:** Validation, Methodology, Investigation. **Jeongyun Heo:** Resources, Methodology, Data curation. **Hwapyung Jung:** Software, Investigation. **Thet Thet Swe:** Resources, Investigation. **Iman Zare:** Visualization, Investigation. **Guosheng Song:** Methodology, Data curation. **Alireza Hassani Najafabadi:** Formal analysis, Data curation. **Junmin Lee:** Visualization, Investigation. **Hyun-Do Jung:** Validation, Data curation. **Jong Seung Kim:** Validation, Data curation. **Sunghoon Hur:** Validation, Resources. **Hyun-Cheol Song:** Validation, Resources. **Sung-Gyu Park:** Resources, Investigation. **Kunyu Zhang:** Resources, Methodology. **Pengchao Zhao:** Methodology, Data curation. **Liming Bian:** Software, Investigation. **Se Hoon Kim:** Software, Resources, Formal analysis. **Juyoung Yoon:** Validation, Data curation. **Jae-Pyoung Ahn:** Validation, Resources. **Hong-Kyu Kim:** Supervision, Resources, Project administration, Conceptualization. **Heemin Kang:** Writing – review & editing, Supervision, Project administration, Funding acquisition, Conceptualization.

## Ethics approval and consent to participate

The animal studies have been approved by the Animal Care and Use Committee of Korea Institute of Science and Technology (KIST), and all handling of mice was performed in accordance with the institutional regulations (KIST-2020-019).

## Declaration of competing interest

The authors declare the following personal relationships which may be considered as potential competing interests: Iman Zare is currently employed by Sina Medical Biochemistry Technologies Co., Ltd. Heemin Kang is an editorial board member for Bioactive Materials and was not involved in the editorial review or the decision to publish this article.
