# Supplementary material for: Dynamic hierarchical ligand anisotropy for competing macrophage regulation in vivo
Source: Bioact Mater. 2025 Jan 19;47:121–35. doi: 10.1016/j.bioactmat.2025.01.009 (PMC11787691; doi:10.1016/j.bioactmat.2025.01.009)
Supplement: Multimedia component 1 [file mmc1.docx]

**SUPPORTING INFORMATION**

**Dynamic Hierarchical Ligand Anisotropy for Competing Macrophage Regulation *In Vivo***

**Supplementary materials and methods**

*1. Anisotropy analysis via transmission electron microscopy (TEM)*

The precisely tailored different-scale anisotropy, shape, and dimensions (length and diameter) of the nanoscale anisotropy-tailored Au and microscale anisotropy-tailored Fe_3_O_4_ were verified via TEM imaging (Talos G2 and Titan^TM^ 80-300, Thermo Fisher, USA). The acquired TEM images were analyzed using ImageJ software to calculate their dimensions.

*2. Selective area electron diffraction (SAED) analysis*

The atomic planes of the crystalline Au in the nanoscale anisotropy-tailored Au and of the crystalline Fe_3_O_4_ in the microscale anisotropy-tailored Fe_3_O_4_ were verified via SAED analysis (Titan^TM^ 80-300) by using a camera length of 6 cm. The acquired SAED pattern images exhibiting multiple homocentric diffraction rings were each identified for their respective planes of Au [(111), (002), (022), and (113)] and the Fe_3_O_4_ [(220), (311), (400), (511), and (440) planes].

*3. High-resolution transmission electron microscopy (HR-TEM) and Fast Fourier transform (FFT) analysis*

The atomic planes of crystalline Au in the nanoscale anisotropy-tailored Au and of the crystalline Fe_3_O_4_ in the microscale anisotropy-tailored Fe_3_O_4_ were verified via HR-TEM imaging (Titan^TM^ 80-300) and FFT analysis. The HR-TEM carried out at an accelerating voltage of 300 kV revealed the average d-spacing between adjacent lattice fringes of each respective crystalline lattice plane in Au [2.38 Å for the (111) plane and 2.06 Å for the (200) plane] and Fe_3_O_4_ [3.01 Å for (220) plane] that were determined and labeled according to the available data as previously reported. The FFT analysis of HR-TEM images taken at low magnification (aligned along the zone axis) revealed periodic bright spots corresponding to the (111) and (200) planes of Au and the (220) plane of Fe_3_O_4_.

*4. Dynamic light scattering (DLS) and zeta potential measurements*

The homogeneous size (hydrodynamic diameter) of N1 and M1 before and after the formation of their silica layer envelop was verified via DLS measurement (Zetasizer Nano ZS90, Malvern Panalytical, UK). The change in the surface charge of N2 upon serial ligand exchange (serially capped with CTAB, PSS, and finally citrate) was also verified using the same equipment.

*5. UV-Vis spectrophotometer analysis*

The successful coupling of the nanoscale anisotropy-tailored Au (exhibiting a red shift in their absorption peaks with increasing anisotropy) on the surface of microscale anisotropy-tailored Fe_3_O_4_ (exhibiting consistent absorption peaks regardless of their shapes) that constitute hierarchical nanostructures was verified via UV-Vis spectrophotometer (Mega-800, Scinco) analysis before and after coupling. The multi-scale anisotropy-controlled hierarchical nanostructures (“M1+N1”, “M1+N2”, “M2+N1”, and “M2+N2” groups) were subjected to the analysis. Extinction spectra were measured using a 1 cm-path length quartz cuvette in the 400 to 1100 nm wavelength range.

*6.* *X-ray diffraction (XRD) analysis*

The atomic planes of crystalline Au in the nanoscale anisotropy-tailored Au and their co-presence with crystalline Fe_3_O_4_ in the microscale anisotropy-tailored Fe_3_O_4_ in the multi-scale anisotropy-tailored hierarchical nanostructures was verified via XRD (D/MAX-2500V/PC, Rigaku) analysis carried out using Cu Kα radiation. The diffraction peaks of Au and Fe_3_O_4_ were indexed according to their respective reference data.

*7. Hierarchical structure analysis via high-angle annular dark-field scanning transmission electron microscopy (HAADF-STEM)*

The hierarchical structures of nanoscale anisotropy-tailored Au coupled on the surfaces of microscale Fe_3_O_4_ in the multi-scale anisotropy-tailored hierarchical nanostructures (M1+N1, M1+N2, M2+N1, and M2+N2) were verified via HAADF-STEM imaging (Talos G2, Thermo Fisher, USA). The imaging conditions were set as an accelerating voltage of 200 kV, a collection semi-angle of 38-200 mrad, a convergence semi-angle of 11.8 mrad, a pixel dwell time of 3 μs, a pixel area of 1024 X 1024, an electron probe size of 0.2 nm, an emission current of 185 μA, and a probe current of 185 pA. The acquired HAADF-STEM images were analyzed using ImageJ software to calculate the nanoscale and microscale aspect ratio in the hierarchical nanostructures, the number and the total surface area of Au per Fe_3_O_4_, and the surface area of each Fe_3_O_4_.

*8. Energy-dispersive X-ray spectroscopy (EDS) mapping*

The elemental conformation of nanoscale anisotropy-tailored Au (Au) and microscale anisotropy-tailored Fe_3_O_4_ (Fe and Si) that constitute the multi-scale anisotropy-tailored hierarchical nanostructures (“M1+N1”, “M1+N2”, “M2+N1”, and “M2+N2” groups) were characterized via EDS mapping (SuperEDS, EDAX, Bruker). Each imaging was performed for 15 min with the beam conditions set as follows: an accelerating voltage of 200 kV, a pixel dwell time of 5 ms, and a probe current of 2.13 nA.

*9. Vibrating sample magnetometry (VSM)*

The reversibly manipulable magnetic properties of the nanoscale anisotropy-tailored Au-coated microscale anisotropy-tailored Fe_3_O_4_ that are retained even after becoming multi-scale anisotropy-tailored hierarchical nanostructures were verified via VSM measurements (EV9-380, MicroSense) at 27 ℃. The hysteresis loop of the magnetic moments in response to a magnetic field strength (Oe) ranging from -19,000 to 19,000 was presented after normalization to the respective dry weight of each sample (emu/g).

*10. Fourier transform infrared spectroscopy (FTIR) analysis*

The serial changes in the characteristic chemical bonds of the hierarchical nanostructures (“M1+N1”, “M1+N2”, “M2+N1”, and “M2+N2”) after PVP-stabilization, PEG linker-coupling, and finally the ligand-coupling were verified via FTIR analysis (Nicolet iS10, Thermo Fisher Scientific). All samples were dried and prepared in KBr pellets before their measurement, and the acquired absorption peaks were identified for each characteristic chemical bond associated with the serial changes.

*11. Synthesis of the substrate with magnetically manipulable multi-scale ligand anisotropy-tailored hierarchical nanostructures*

To fabricate the substrate displaying magnetically manipulable multi-scale ligand anisotropy, hierarchical nanostructures (“M1+N1”, “M1+N2”, “M2+N1”, and “M2+N2”) were individually coupled on the substrate at equivalent densities by using elastic polymer linkers. To this end, amine-functionalized substrate surfaces were first prepared using cell culture-grade glass coverslips (22 x 22 mm), which were pre-sterilized by the treatment in 1:1 HCl-MeOH solution for 45 min and then activated with the hydroxyl group (−OH) by the treatment in sulfuric acid (H_2_SO_4_) for 1.5 h. The hydroxyl group-activated substrate surfaces were amine-functionalized in 1:1 APTES-ethanol solution for 2 h under dark conditions, serially washed with DI water and ethanol, and then dried in an oven at 100 °C for 1 h.

Sequentially, the hierarchical nanostructures were coupled with elastic PEG linkers to allow their *in situ* magnetic manipulation. To this end, 2 mL of each multi-scale anisotropy-tailored hierarchical nanostructure was treated with 2 mL of 0.1 mM thiol-poly(ethylene glycol)-N-hydroxysuccinimide (SH-PEG-NHS, Mw: 10,000 Da) at 25 ℃ for 16 h under dark conditions for the thiol groups in PEG linkers to be coupled on Au via thiol-gold bonds. Followingly, the amine-functionalized substrates were treated with 2 mL of the PEG linker-coupled hierarchical nanostructure at 25 ℃ for 16 h under dark conditions for the NHS groups in PEG linkers to be coupled with the amine groups present on the substrate surface, then washed with DI water. The leftover NHS groups in N-Au of hierarchical nanostructures that had not reacted with substrate surfaces were then coupled with the amine group-presenting cell-adhesive RGD ligands by treating the resultant substrates with 2.5 mL of 1 mM Cyclo (-RGDyK) (AS-61183-5, AnaSpec, Inc] in DI water under shaking for 16 h in dark conditions.

Finally, to avoid any non-specific cell adhesion, amine-functionalized substrate surfaces not coupled with PEG linker-coupled hierarchical nanostructures were passivated by being treated with 2 mL of 0.1 mM methoxy-PEG-succinimidyl-carboxymethyl-ester (M-SCM-5000, Mw: 5,000 Da) at 25 ℃ for 2 h under dark conditions, then washed with DI water.

*12. Scanning electron microscopy (SEM) imaging*

The homogeneous distribution of the multi-scale ligand anisotropy-tailored hierarchical nanostructures (“M1+N1”, “M1+N2”, “M2+N1”, and “M2+N2” groups) that are elastically coupled over the substrates was verified via SEM imaging [Quanta 250 FEG SEM (FEI)]. Before subjection to SEM imaging, the samples were dried in a vacuum and coated with platinum. The acquired SEM images were analyzed using ImageJ software to calculate the density of the hierarchical nanostructures (particles per μm^2^). The total ligand area per area (μm^2^) of the substrate was calculated by multiplying the number of hierarchical nanostructures presented on substrates per area (μm^2^) by the surface area of each nanoscale anisotropy-tailored Au coupled on each microscale anisotropy-tailored Fe_3_O_4_.

*13. Nanodrop analysis*

Similar number of cell-adhesive RGD ligands coupled on the N-Au particles across the multi-scale ligand anisotropy-tailored hierarchical nanostructures (“M1+N1”, “M1+N2”, “M2+N1”, and “M2+N2” groups) was measured via A280 method [Nanodrop One, Thermofisher Scientific]. The measurement was conducted through the following procedure: 1 mL of 0.5 mM Cyclo (-RGDyK) (AS-61183-5, AnaSpec, Inc] in DI was conjugated to the 500 μL of PEG linker-coupled multi-scale ligand anisotropy-tailored hierarchical nanostructures in the solution via EDC/NHS reaction under shaking for 16 h in dark condition. The particles were sedimented by centrifugation, and the supernatants containing RGD unbound to gold nanoparticles were collected. A280 method was applied to supernatants, and the concentrations of RGD unbound to gold nanoparticles were calculated according to RGD calibration curve. The concentrations of RGD bound to gold nanoparticles were calculated by subtracting the measured RGD concentration in the supernatant from the initially added RGD concentration. The number of RGD molecules in the solution was divided by the number and surface area of the gold nanoparticles present in the solution to calculate the number of RGD molecules per gold nanoparticle and per unit area of gold nanoparticle surface, respectively.

*14. In situ atomic force microscopy (AFM)*

The *in situ* reversible axial manipulation of the hierarchical nanostructures was achieved through the reversible application of the permanent magnet (285 mT) over the substrates that directed their upward movement from the substrate surface [“Unpressed” (UP) group] or downward movement to the substrate surface [“Pressed” (P) group], respectively. Such reversible movement of the nanostructures was verified via AFM imaging (XE-100 System, Asylum Research) of an identical area. The “Non-affected” (NA) group was used as control under no magnetic application. The imaging was carried out in AC, air mode at 25 ℃ by using an AFM cantilever (SSS-SEIHR-20, Nanosensors) with a spring constant of 5-37 N/m and a resonance frequency of 96 - 175 kHz. Height changes of the “M1+N2” and “M2+N1” groups independently coupled on the substrates were analyzed using Igor Pro 6.12A and ImageJ software to confirm *in situ* reversible magnetic manipulation.

*15. Reversible manipulation of multi-scale ligand anisotropy for macrophage regulation in vitro*

The cellular regulatory effect of ligand anisotropy tailored in multi-scale as well as their reversible axial manipulation was verified with macrophages (RAW 264.7, passage 5, ATCC). To this end, macrophages were plated at 8.5 x 10^4^ cells/cm^2^ (only at the beginning of culturing) on the substrates presenting magnetically manipulable hierarchical nanostructures (pre-sterilized with UV light irradiation). The culturing conditions included a basal growth medium [high glucose Dulbecco's Modified Eagle Medium (DMEM) including 10 % fetal bovine serum (FBS), and 50 U/mL penicillin/streptomycin] at 37 ℃ under 5% CO_2_. The effect of ligand anisotropy tailored at multi-scale on macrophage adhesion was investigated after 24 h of culturing on substrates with hierarchical nanostructures (“M1+N1”, “M1+N2”, “M2+N1”, and “M2+N2” groups). Among various hierarchical nanostructures, the groups that exhibit disparate ligand isotropy and anisotropy at multi-scale (“M1+N2” and “M2+N1” groups) were chosen for the examination of the reversible axial-manipulation effect on macrophages. For such assessment, macrophages were cultured on substrates with or without [“Non-affected” (NA)] the magnet placed over or under the substrates that direct upward or downward movement of the nanostructures to induce the “Unpressed” (UP) or “Pressed” (P) state, respectively. For their reversible axial manipulation, upward or downward magnet application conditions were either switched or maintained every 12 h (after 24 h of culturing) up to 48 h (“NA-NA-NA”, “NA-P-NA”, “P-NA-P”, “P-P-P”, “NA-UP-NA”, “UP-NA-UP”, and “UP-UP-UP”).

The effect of the axial manipulation of ligand multi-scale anisotropy on macrophage phenotypic polarization (pro-regenerative M2 or pro-inflammatory M1 polarization) was investigated after 36 h of culturing on substrates with hierarchical nanostructures (“M1+N2” or “M2+N1” group) with or without the magnet placed over or under the substrates. The culturing conditions of 37 ℃ under 5% CO_2_ were retained but either in the pro-regenerative M2 medium [basal growth medium including the mixture of 20 ng/mL interleukin-4 and 20 ng/mL interleukin-13 or in the pro-inflammatory M1 medium [basal growth medium including the mixture of 10 ng/mL lipopolysaccharide and 10 ng/mL recombinant interferon-gamma]. The regulation of macrophage polarization via their adhesion was assessed in the pro-regenerative M2 or pro-inflammatory M1 medium supplemented with one of the following inhibitors specific for the adhesion-related molecular mechanism: 10 µM blebbistatin, 2 µg/mL cytochalasin D, or 50 µM Y27632 as specific inhibitors for myosin II, actin polymerization, or ROCK, respectively.

*16. Immunofluorescence imaging and western blotting analysis of macrophage regulation*

The regulation of macrophage adhesion and following polarization by reversible manipulation of hierarchical nanostructures exhibiting ligand anisotropy tailored at multi-scale was verified via confocal immunofluorescence imaging (LSM700 confocal microscope, Carl Zeiss), super-resolution immunofluorescence imaging (Lattice SIM 5, Carl Zeiss), flow cytometry [Fluorescence-activated Cell Sorting (FACS) Calibur, BD CellQuest Pro software, BD Biosciences], and western blotting (Linear Image Quant LAS 4000 mini chemiluminescent imaging system).

For confocal imaging and high resolution imaging, macrophages cultured on the substrate presenting hierarchical nanostructures were fluorescently immunostained through the following procedure: the macrophages were fixed and permeabilized in 4% paraformaldehyde (PFA), blocked in blocking buffer [phosphate-buffered saline (PBS) including 3% bovine serum albumin (BSA) and 0.1% Triton-X-100], and then treated in blocking buffer containing primary antibodies followed by blocking buffer containing fluorescent dye-conjugated secondary antibodies, phalloidin, and DAPI. The primary antibodies used in these experiments are as follows: integrin β1 (catalog no. sc-374429, Santa Cruz Biotechnology), vinculin (catalog no. V9131, Sigma-Aldrich), Arg-1 (catalog no. ab91279, Abcam), iNOS (catalog no. sc-7271, Santa Cruz Biotechnology), Rho A (catalog no. ab187027, Abcam), ROCK 2 (catalog no. sc-398519, Santa Cruz Biotechnology), and 9EG7 (catalog no. 553715, BD Biosciences).

Serially, the fluorescently immunostained macrophages were mounted on glass slides to be subjected to confocal imaging and super-resolution imaging, with the laser exposure and image acquisition conditions retained for all the images to ensure objective comparison among the compared groups. The acquired confocal images were analyzed using ImageJ software to calculate the adhered planar cell density (DAPI-positive cells/cm^2^), cell size (actin-positive area), and aspect ratio of cells (major axis/minor axis). The fluorescence intensities of each protein of concern were analyzed via the histogram function of ImageJ software, and the acquired super-resolution images were reconstructed by the theoretical optical transfer function provided by the manufacturer.

For the quantitative characterization of macrophage polarization, macrophages cultured in either pro-regenerative M2 or pro-inflammatory M1 medium on substrates with hierarchical nanostructures were subjected to western blotting analysis. To this end, the macrophage proteins [Arg-1 (pro-regenerative M2 marker), iNOS (pro-inflammatory M1 marker), and GAPDH (housekeeping gene)] were collected via centrifugation with a mixture solution containing 400 µL of PRO-PREP™ protein extraction buffer (iNtRON Biotechnology) and 10 of µL protease inhibitor cocktail. The total concentrations of the extracted proteins were quantitated using a BCA Protein Assay Kit (Thermo Scientific™ Pierce™), mixed with loading dyes, denatured via boiling, separated via 10% sodium dodecyl sulfate (SDS)-polyacrylamide gel electrophoresis (PAGE), and then transferred to polyvinylidene fluoride (PVDF) membranes via electroblotting. Sequentially, the transferred proteins were subjected to electrophoresis, blocked in blocking buffer [tris-buffered saline including 0.1% Tween 20 (TBST) and 5% skimmed milk], and then treated in blocking buffer containing primary antibodies of iNOS (135,000 Da), Arg-1 (37,000 Da), and GAPDH (36,000 Da) followed by blocking buffer containing anti-horseradish peroxidase (HRP)-conjugated secondary antibodies. Finally, the resultant membranes were placed in an ECL western blotting reagent (Immobilon Western Chemiluminescent HRP Substrate, MERCK-Millipore) for their western blotting analysis, in which the relative protein expression levels were exhibited after normalization to GAPDH.

*17. Cell attachment assay*

The regulation of macrophage adhesion by hierarchical nanostructures exhibiting ligand anisotropy tailored at multi-scale was examined via crystal violet staining and large-field optical microscope imaging (Eclipse Ts 2, Nikon). Macrophages were cultured for 24 h, and were fixed and permeabilized in 4% PFA, blocked in blocking buffer (PBS including 3% BSA and 0.1% Triton-X-100). After fixation, 0.05 % (w/v) crystal violet in PBS solution was applied to cells for 10 minutes at room temperature, followed by 4 times washing to eliminate the excess crystal violet. The large-field imaging in randomly selected fields was conducted by optical microscope. And the acquired images were analyzed using ImageJ software to calculate the adhered planar cell density (DAPI-positive cells/cm^2^).

*18. Immunolabelling and imaging analysis of recruited macrophage integrins*

The effect of the ligand anisotropy tailored at multi-scale on the recruitment of macrophage integrins was verified via their immunolabelling and analysis through SEM imaging [Quanta 250 FEG SEM (FEI)]. For their clear discrimination from 45 nm N1 nanoparticles on the surface of the hierarchical nanostructures, 20 nm Au was synthesized by stirring 30 mL of 1 mM HAuCl_4_·3H_2_O in DI water at 100 ℃ for 30 min, which was then added with 3 mL of 38.8 mM Na_3_C_6_H_5_O_7_ in DI water and stirred at 100 ℃ for another 15 min. Termination of the reaction and cooling down the mixture solution to 25 ℃ changed the color of the solution from yellow to burgundy red, resulting in a 20 nm Au nanoparticle suspension. The 20 nm Au were then serially treated with 0.1 M 1,4 piperazine bis (2-ethanosulfonic acid) (PIPES) buffer containing the (H+L) IgG secondary antibody (goat anti-mouse, Abcam), 1% BSA, and 0.1% Tween 20 at 37 ℃ for 16 h.

Before immunolabelling, the cultured macrophages were washed with PIPES buffer, fixed with 4% PFA, permeabilized with blocking buffer [DI water containing 0.5% Triton X-100, HEPES, sodium chloride (NaCl), magnesium chloride (MgCl_2_), and sucrose], treated in blocking buffer containing integrin β1 primary antibody, and then blocked with 5% goat serum. Followingly, the macrophages were immunolabelled for their integrins via incubation in PIPES buffer containing secondary antibody-coated 20 nm Au nanoparticles for 16 h, fixed with 2.5% glutaraldehyde, treated in PIPES buffer containing 1% osmium tetroxide (contrast enhancer), and then dried to be imaged via SEM.

Consequently, macrophages immunolabelled for their integrins were analyzed via SEM imaging to investigate the effect of reversibly manipulable ligand anisotropy tailored at multi-scale on integrin recruitment. For their clear examination, macrophages (light blue) and integrin β1-immunolabelling Au nanoparticles (pink) were colored in the acquired SEM images, which were analyzed for the calculation of the number of integrin β1-labeling Au nanoparticles on macrophages per hierarchical nanostructure.

*19. Host cell regulation via the reversible manipulation of ligand anisotropy tailored at multi-scale in vivo*

The host macrophage regulation by the reversible manipulation of hierarchical nanostructures exhibiting ligand anisotropy tailored at multi-scale was verified as a proof-of-concept. First, silicon substrates (for minimal breaking) with multi-scale ligand anisotropy-tailored hierarchical nanostructures were implanted into the subcutaneous pockets of 2-month-old nude mice (balb/c, male, total 48) with the approval of the Animal Care and Use Committee of Korea Institute of Science and Technology (KIST) (approval number: KIST-2020-019). The biocompatibility of the substrate with multi-scale ligand anisotropy-tailored hierarchical nanostructures in mice was examined both locally (subcutaneous tissue) and systemically (heart, liver, and kidney) through histological analysis at 0 and 7 d post-implantation. The collected tissues and organs of concern underwent the following procedure washing with PBS, fixing with 10% PFA, dehydrating with increasing ethanol concentration, and embedding in paraffin. Subsequently, 5 μm-thick sections were obtained using a microtome (HistoCore Multicut, Leica RM2125 RTS, Leica Biosystems) which were then mounted on glass slides. The sections were deparaffinized and rehydrated to be co-stained with hematoxylin and eosin (H&E), and the cellular organization of the tissues and organs was evaluated using optical microscopy (Nikon Eclipse Ts2). The stability examination of the substrate with multi-scale ligand anisotropy-tailored hierarchical nanostructures in the dynamic *in vivo* microenvironment was performed by SEM imaging.

Before implantation, the mice were anesthetized with an intraperitoneal injection (mixture of 30 µL alfaxan and 10 µL rompun), and their backs were incised (22 mm) for the implantation of substrates and immediately sutured after the implantation. To minimize the predominant inflammatory host response right after the substrate implantation, an anti-inflammatory mixture (40 ng of IL-4 and 40 ng of IL-13) was injected into the implanted substrate surface by using a pipette. For the axial manipulation of the hierarchical nanostructures *in vivo*, the permanent magnet (285 mT) was either attached or not attached [“Non-affected” (NA)] to the backs or abdomens of mice. In the attached groups, upward or downward movement of the nanostructures to induce the “Unpressed” (UP) or “Pressed” (P) state, respectively, were applied. At 24 h post-implantation, the substrates were retrieved for the analysis of host macrophage regulation via confocal immunofluorescence imaging (LSM700 confocal microscope, Carl Zeiss) and flow cytometry (FACS Calibur, BD CellQuest Pro software, BD Biosciences). The immunofluorescent imaging was performed for the iNOS or Arg-1 proteins with phalloidin and DAPI, while flow cytometry analysis was performed for iNOS or pro-regenerative CD 163 proteins and displayed in histograms using FlowJo software in which the mean fluorescence intensities were presented respectively to the isotype control.

*20. Statistical treatment*

All experiments reported in this study were independently repeated at least twice for obtaining consistent datasets that were quantitatively characterized via GraphPad Prism software (ver. 8.0.2) to determine statistical significance. The statistical analysis for comparing two groups was conducted through two-tailed Student’s *t*-tests, while for comparing multiple groups, one-way analysis of variance (ANOVA) and Tukey-Kramer post-hoc tests were applied. Different asterisk numbers were assigned to p values to indicate statistically significant differences (*p < 0.05; **p < 0.01; ***: p < 0.001), and n values indicate the number of repetitions of experiments.

**Supplementary Figures**


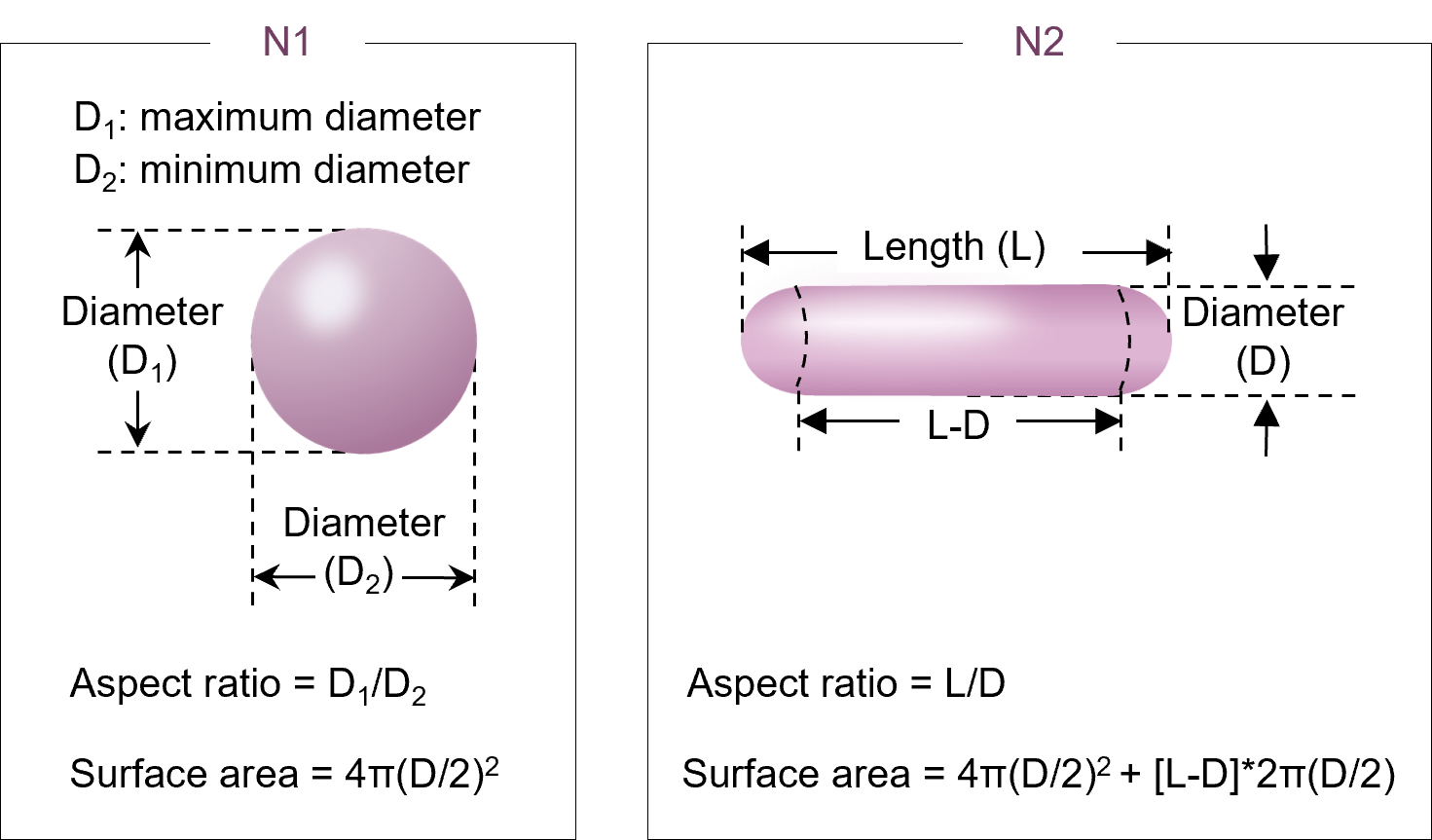


**Fig. S1.** Mathematical formulas for the surface area calculation of nanoscale anisotropy-tailored Au. A schematic description for the mathematical surface area calculation of nanoscale anisotropy-tailored Au (N1: isotropic Au sphere, N2: anisotropic Au rod). The surface area of the spherical N1 was calculated as the surface area of the spherical shape, while that of the N2 exhibiting the rod shape with both ends terminated by hemispheres was calculated by adding the lateral surface area of the rod and the surface area of a full sphere (from the terminally located hemispheres). The aspect ratios were calculated by dividing their major axis by the minor axis.


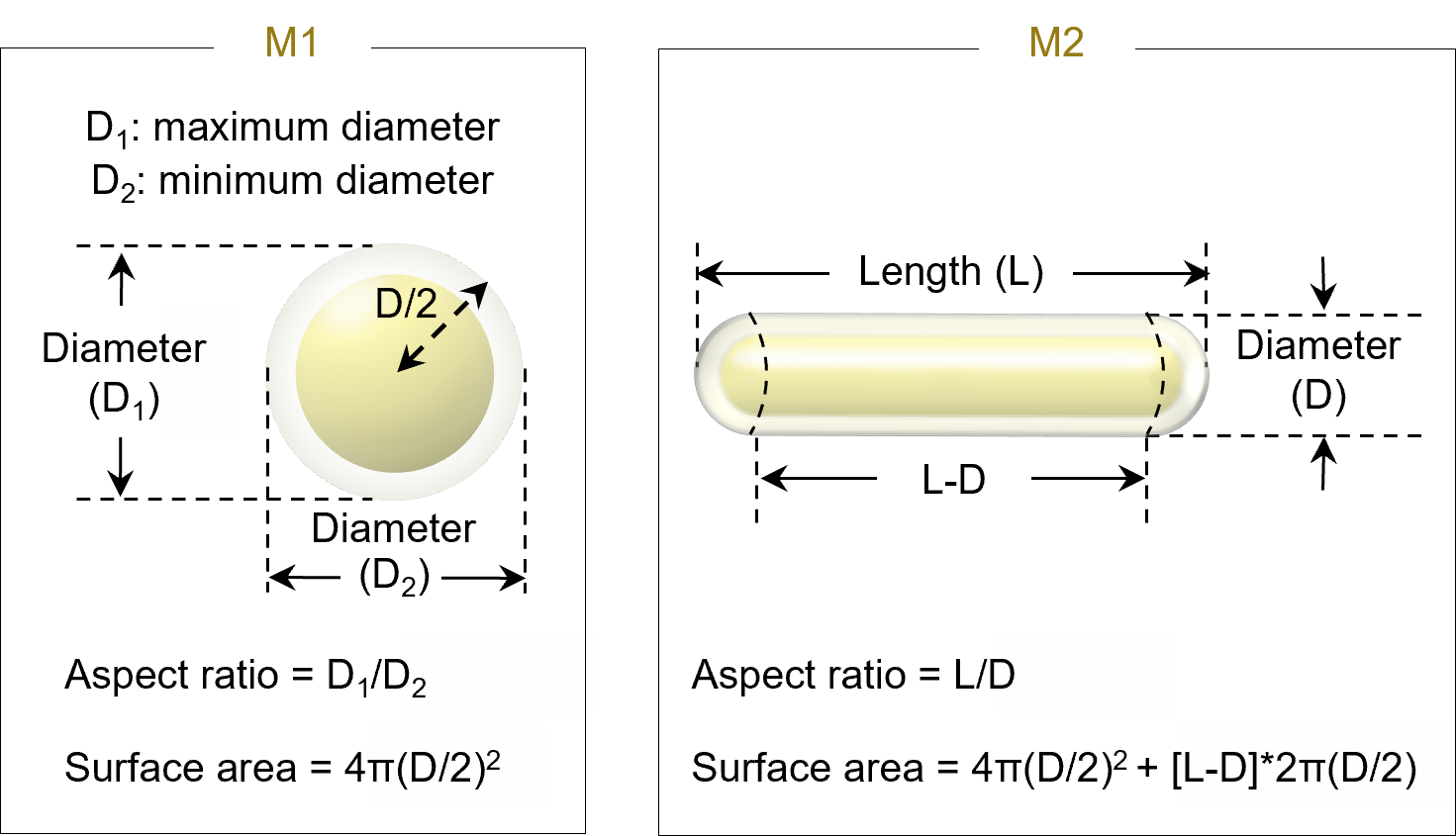


**Fig. S2.** Mathematical formulas for the surface area calculation of microscale anisotropy-tailored Fe_3_O_4_. A schematic description for the mathematical surface area calculation of microscale anisotropy-tailored Fe_3_O_4_ (M1: isotropic Fe_3_O_4_ sphere, M2: anisotropic Fe_3_O_4_ rod). The surface area of the spherical M1 was calculated as the surface area of the spherical shape, while that of the M2 exhibiting the rod shape with both ends terminated by hemispheres were calculated by adding the lateral surface area of the rod and the surface area of a full sphere (from the terminally located hemispheres). The aspect ratios were calculated by dividing their major axis by the minor axis.


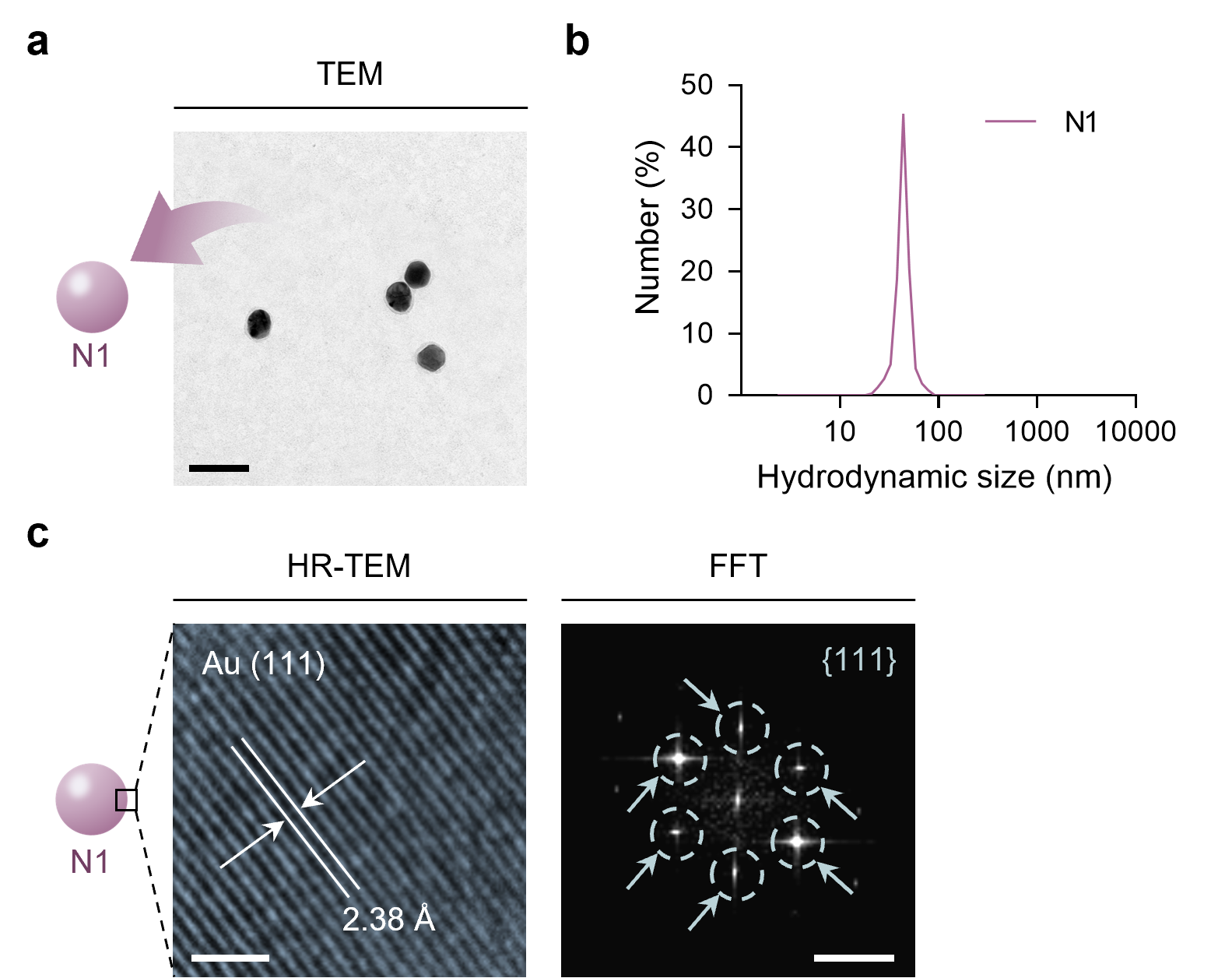


**Fig. S3.** Crystalline structure analysis of nano-isotropic Au sphere. (a) The structural analysis of nano-isotropic Au (N1) with transmission electron microscopy (TEM) image in low magnification (scale bar: 100 nm). (b) The size analysis with dynamic light scattering (DLS). (c) The atomic structure analysis with high-resolution TEM (HR-TEM) and Fast Fourier transform (FFT) images [scale bars: 1 nm (HR-TEM) and 5 nm^-1^ (FFT)]. For N1, TEM image reveals the isotropic shape; DLS reveals the highly homogeneous nanoscale diameter (45 nm); HR-TEM and FFT images reveal the average lattice spacing and crystalline lattice plane, respectively.


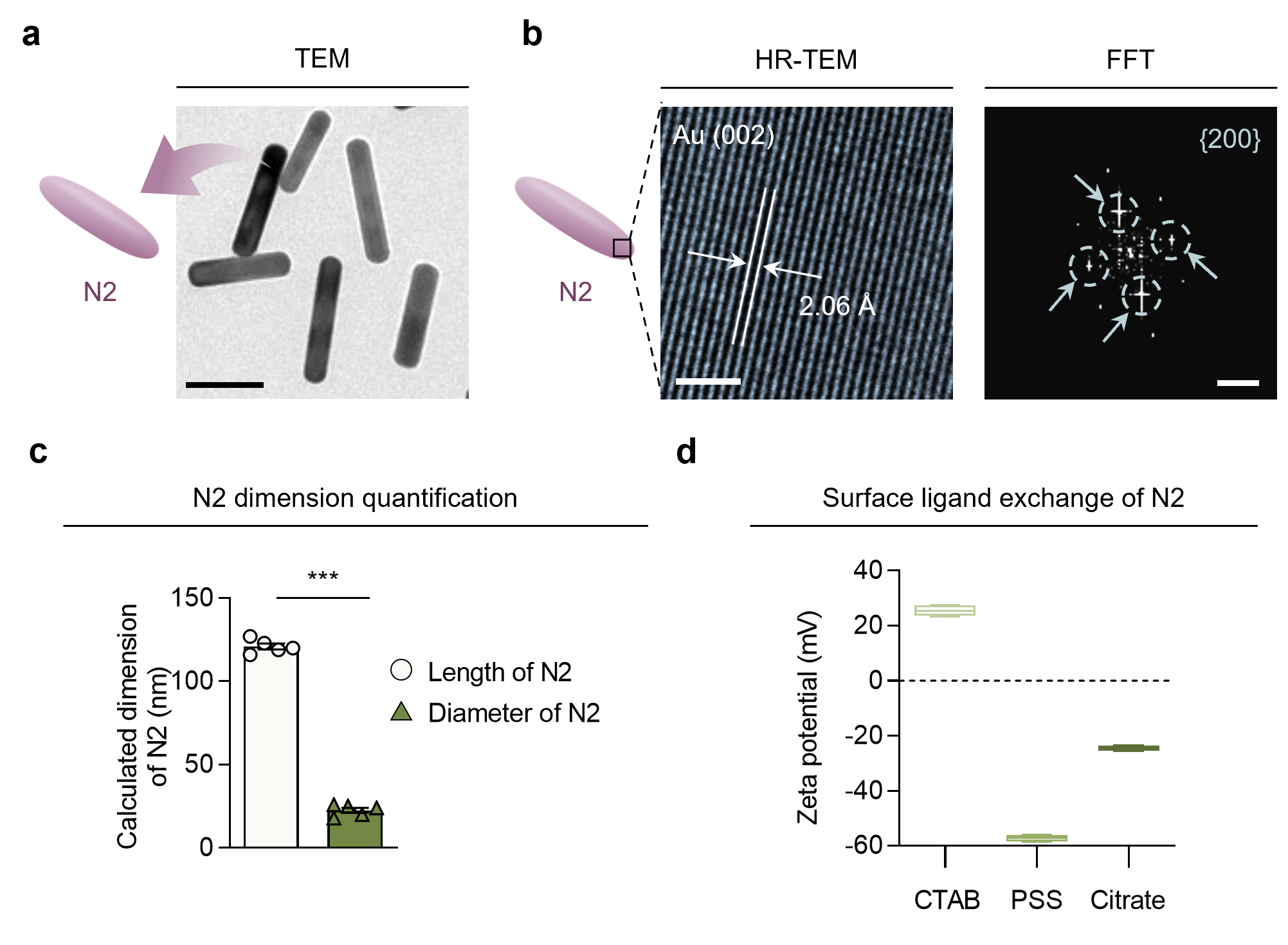


**Fig. S4.** Structural analysis of the nano-anisotropic Au rod. (a) The structural analysis of nano-anisotropic Au (N2) with transmission electron microscopy (TEM) image in low magnification (scale bar: 100 nm). (b) The atomic structure analysis with high-resolution TEM (HR-TEM) and Fast Fourier transform (FFT) images [scale bars: 1 nm (HR-TEM) and 5 nm^-1^ (FFT)]. (c) The calculated dimensions (length and diameter) and (d) zeta potential measurements of the surface charge that changes upon serial ligand exchange (serially capped with CTAB, PSS, and finally citrate). The TEM image reveals the anisotropic shape, and the HR-TEM and FFT images reveal the average lattice spacing and crystalline lattice plane, calculated dimensions reveal the nanoscale length (118 nm) and diameter (18 nm), and zeta potential measurements reveal the surface ligand exchanges of N2. Data are exhibited as the mean ± standard error (n=5). Asterisks assigned to the range of p values (***: p < 0.001) represent statistically significant differences.


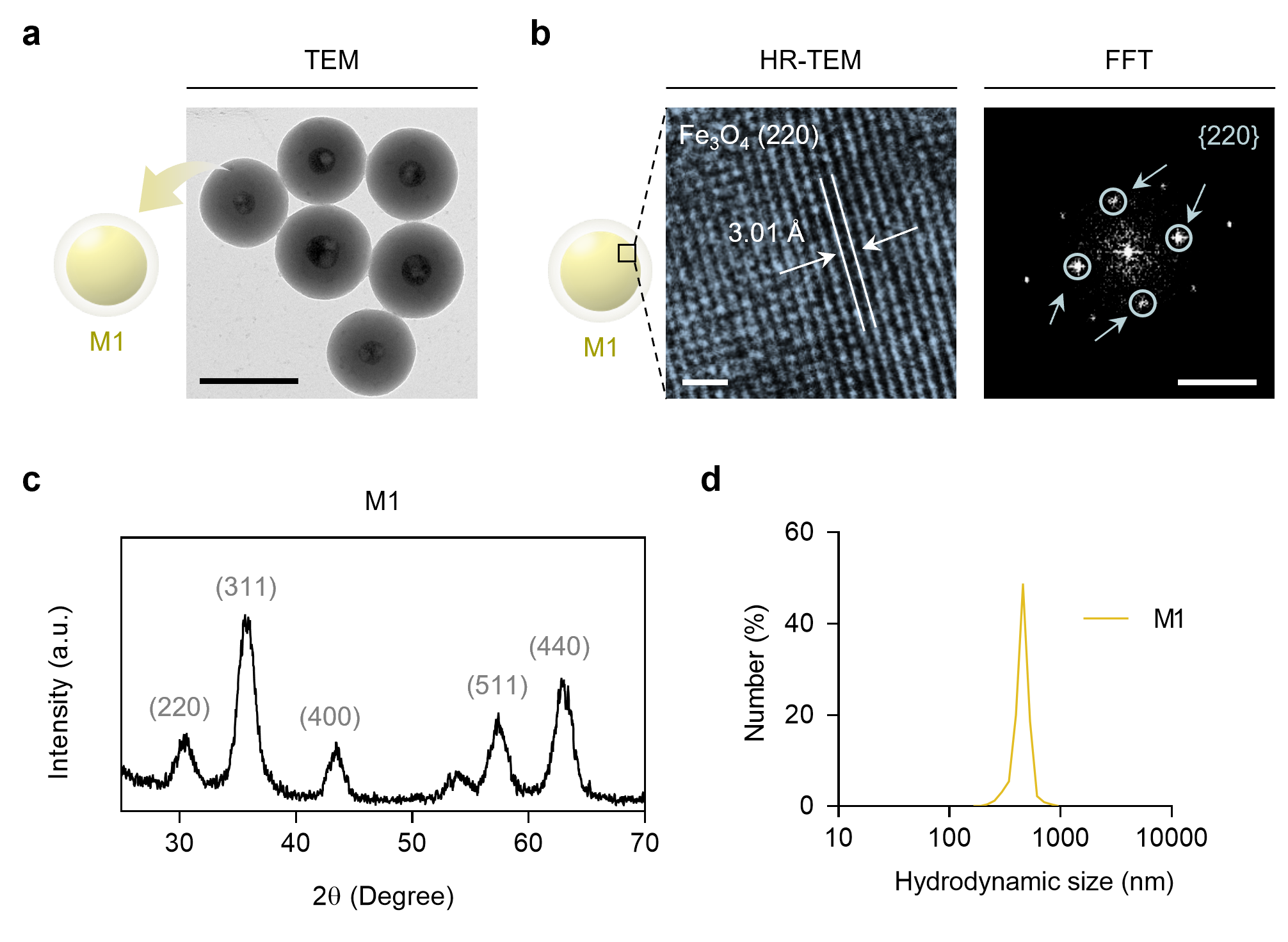


**Fig. S5.** Structural analysis of the magnetic micro-isotropic Fe_3_O_4_ sphere. (a) The structural analysis of micro-isotropic Fe_3_O_4_ (M1) with transmission electron microscopy (TEM) image in low magnification (scale bar: 500 nm). (b) The atomic structure analysis with high-resolution TEM (HR-TEM) and Fast Fourier transform (FFT) images [scale bars: 1 nm (HR-TEM) and 5 nm^-1^ (FFT)]. (c) The crystalline structure analysis with X-ray diffraction (XRD) and (d) size analysis with dynamic light scattering (DLS). The TEM image reveals the isotropic shape, the HR-TEM and FFT images reveal the average lattice spacing and crystalline lattice plane, the XRD reveals the crystalline planes, and the DLS reveals the highly homogeneous microscale diameter (450 nm) of M1.


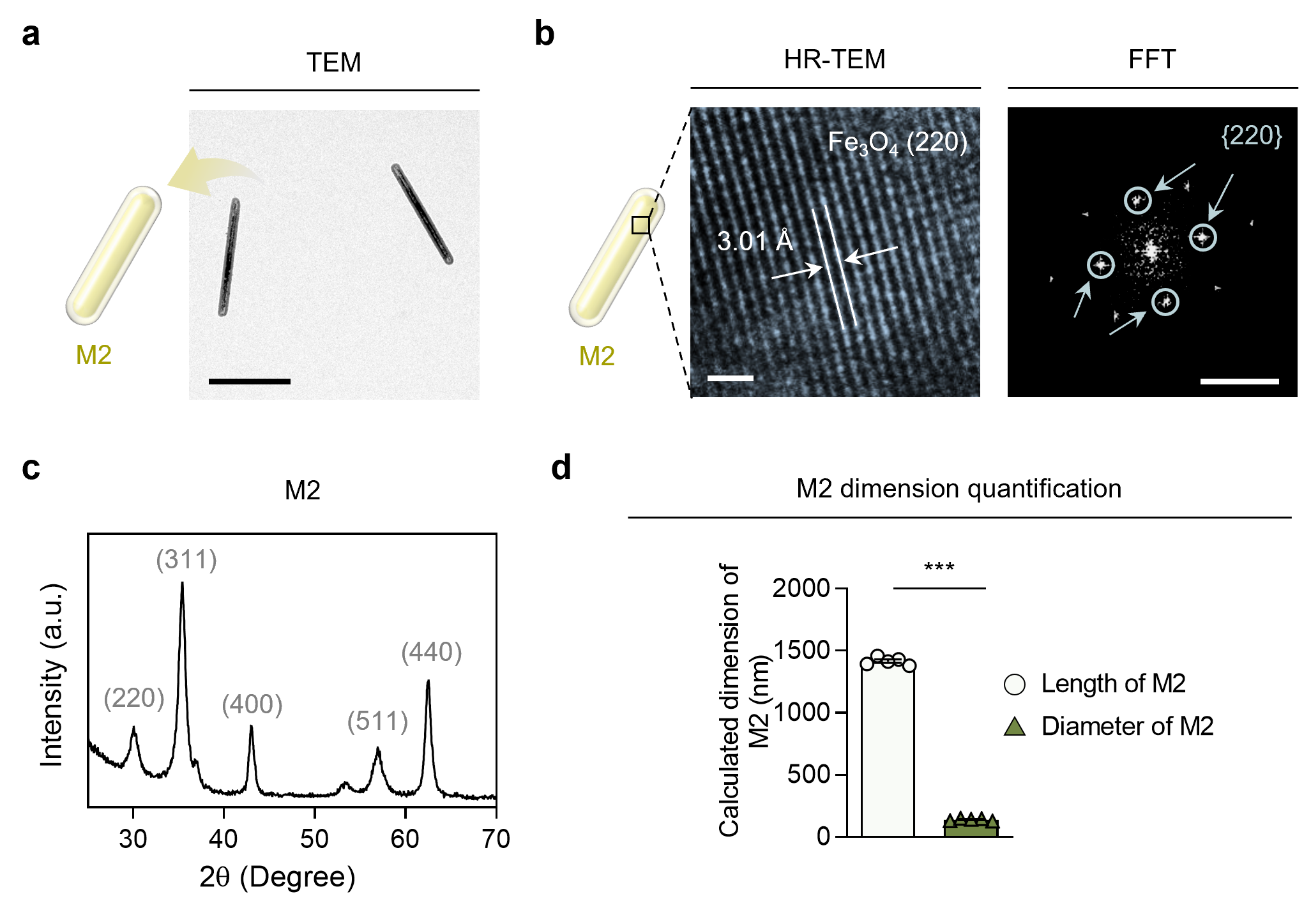


**Fig. S6.** Structural analysis of the magnetic micro-anisotropic Fe_3_O_4_ rod. (a) The structural analysis of micro-anisotropic Fe_3_O_4_ (M2) with transmission electron microscopy (TEM) image in low magnification (scale bar: 500 nm). (b) The atomic structure analysis with high-resolution TEM (HR-TEM) with Fast Fourier transform (FFT) images [scale bars: 1 nm (HR-TEM) and 5 nm^-1^ (FFT)]. (c) The crystalline structure analysis with X-ray diffraction (XRD) and (d) the calculated dimensions (length and diameter). The TEM image reveals the anisotropic shape, the HR-TEM and FFT images reveal the average lattice spacing and crystalline lattice plane, the XRD reveals the crystalline planes, and the calculated dimensions reveal the microscale length (1400 nm) and diameter (140 nm) of M2. Data are exhibited as the mean ± standard error (n=5). Asterisks assigned to the range of p values (***: p < 0.001) represent statistically significant differences.


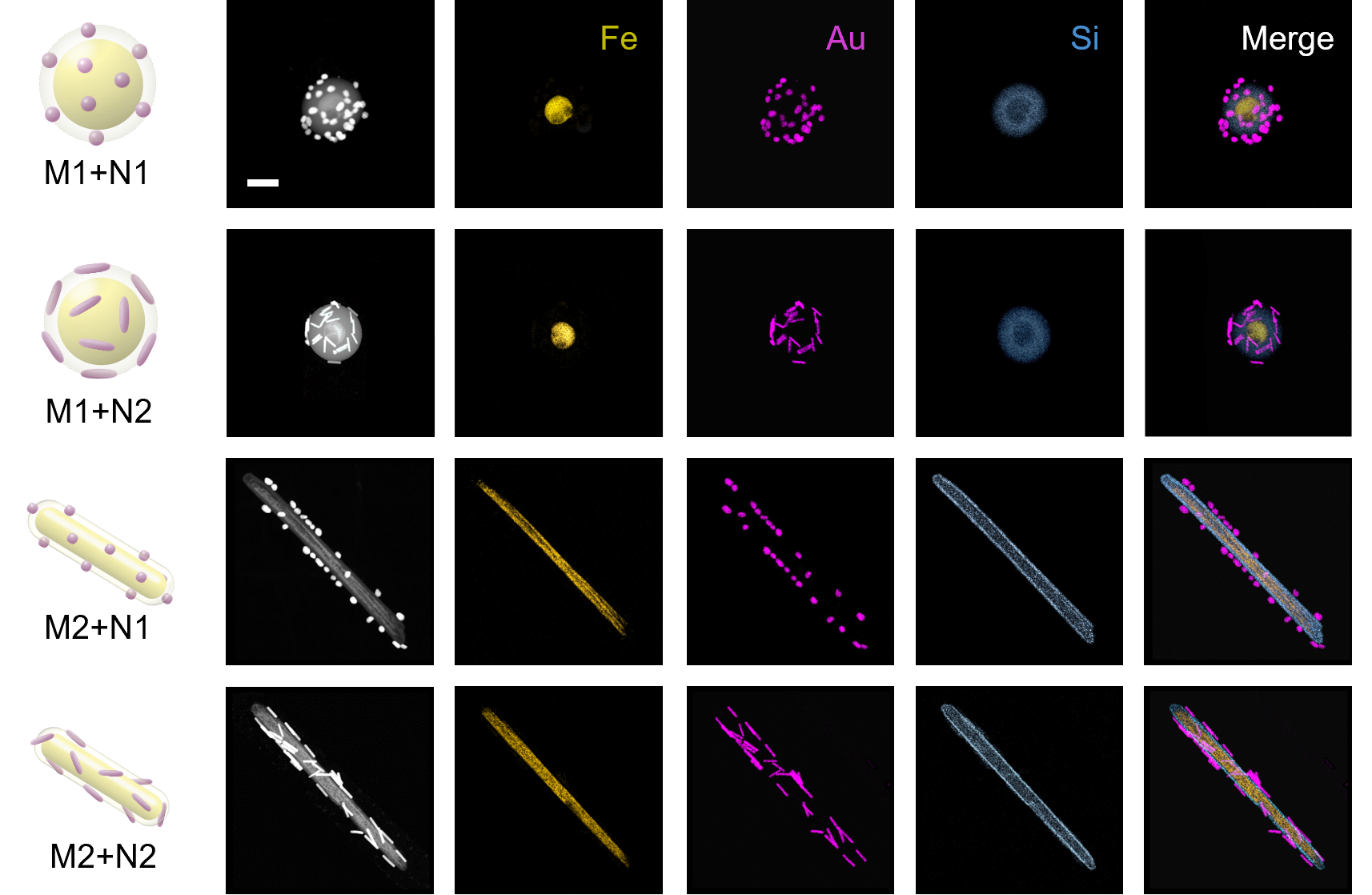


**Fig. S7.** N-Au particles are evenly distributed on the surfaces of M-Fe_3_O_4_ particles in each multi-scale ligand anisotropy-tailored hierarchical nanostructures. HAADF-STEM, EDS mapping (Fe from the Fe_3_O_4_ core and Si from the silica layer envelop of M1 or M2 as well as Au from N1 or N2) images of multi-scale ligand anisotropy-tailored hierarchical nanostructures (“M1+N1”, “M1+N2”, “M2+N1”, and “M2+N2” groups) (scale bar: 200 nm).


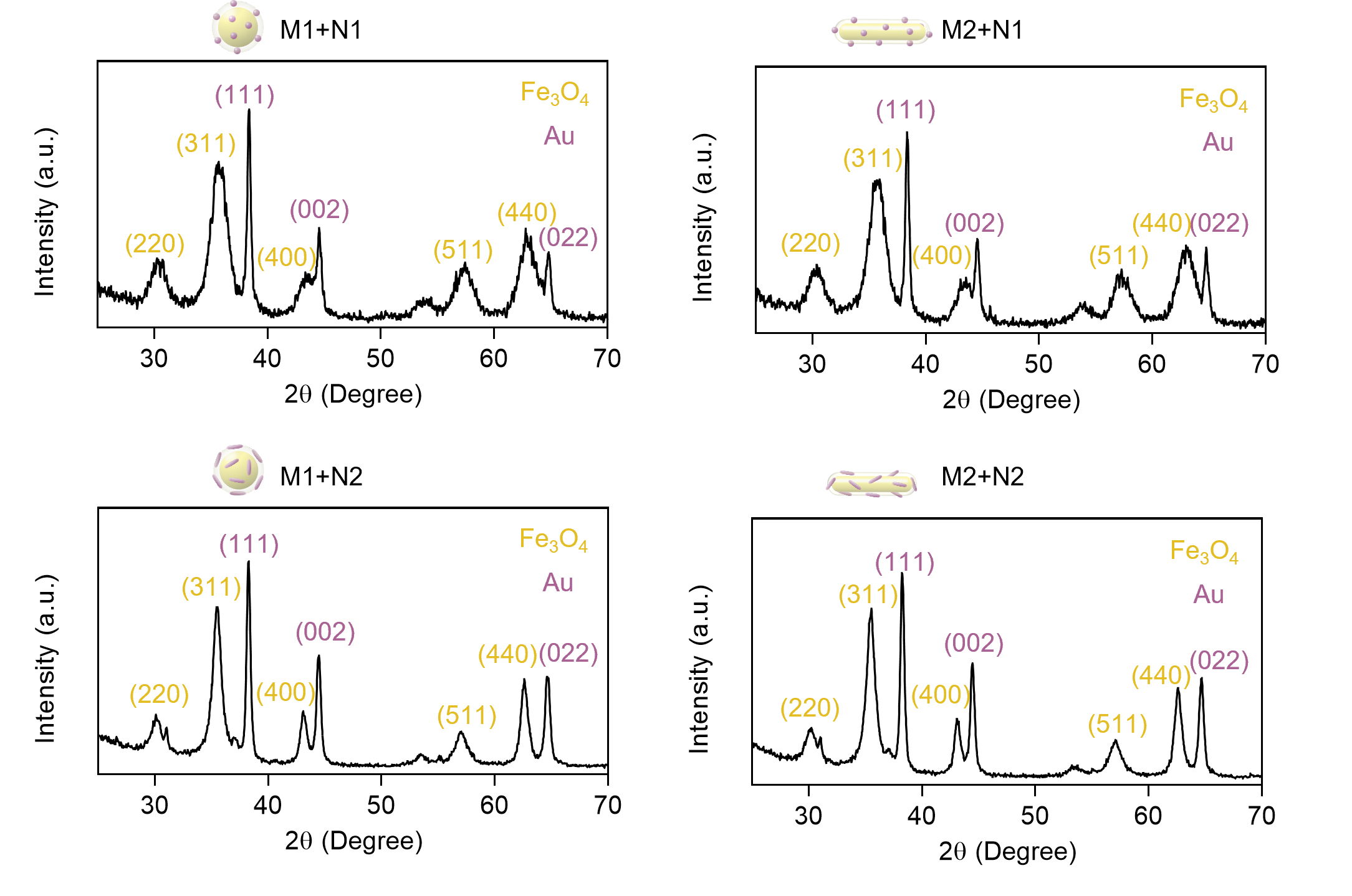


**Fig. S8.** Crystalline phases of Au and Fe_3_O_4_ are preserved in the multi-scale anisotropy-tailored hierarchical nanostructures. X-ray diffraction analysis for the typical diffraction peaks of crystalline Au and Fe_3_O_4_ in nanoscale anisotropy-tailored (isotropic or anisotropic) Au (N1 or N2, respectively) and microscale anisotropy-tailored (isotropic or anisotropic) Fe_3_O_4_ (M1 or M2, respectively) that compose the multi-scale anisotropy-tailored hierarchical nanostructures (M1+N1, M1+N2, M2+N1, and M2+N2). Each crystalline plane of Au [(111), (002), and (022)] and Fe_3_O_4_ [(220), (311), (400), (511), and (440)] are indexed according to their respective reference data.


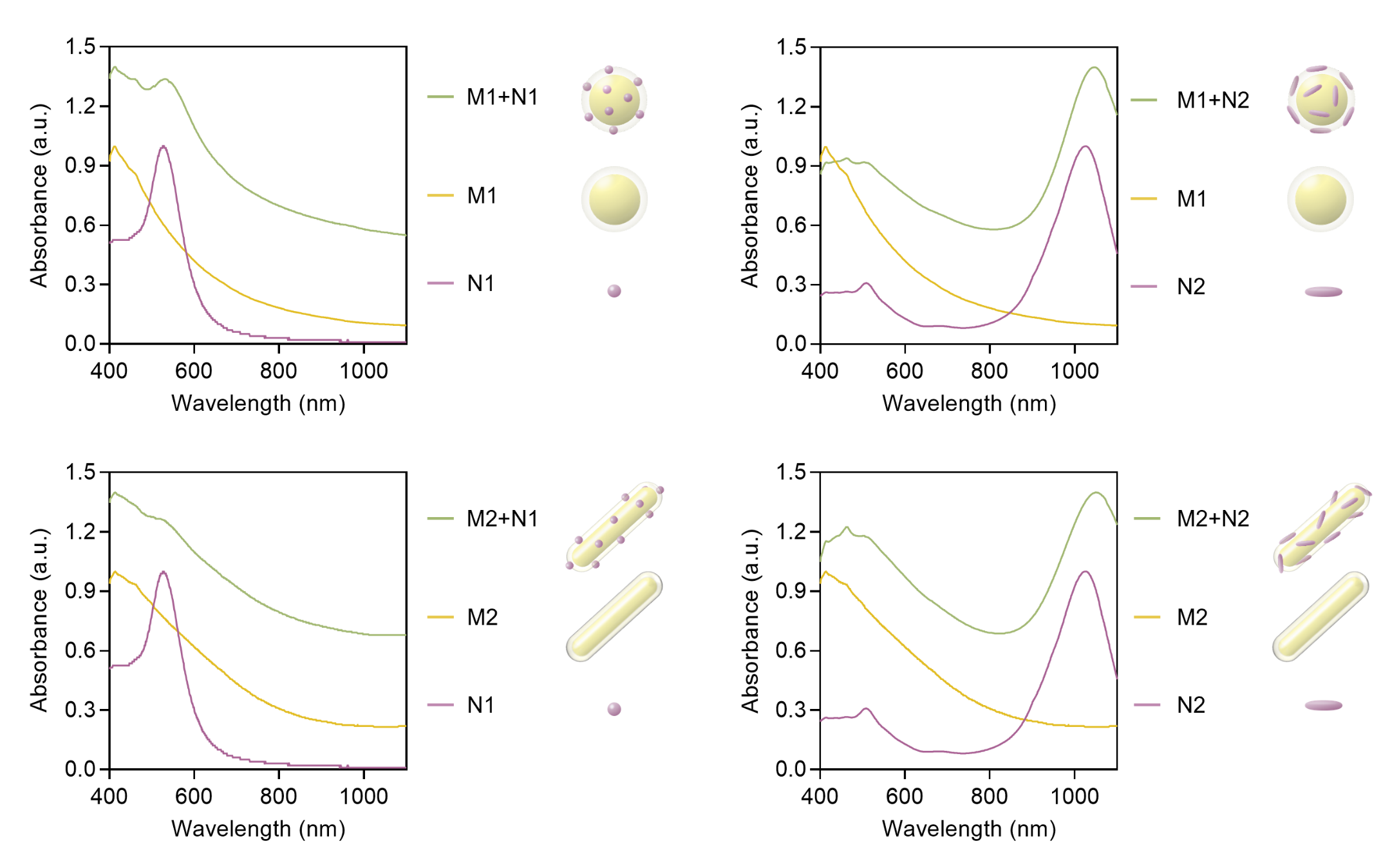


**Fig. S9.** Characteristic absorbance of hierarchical nanostructures containing anisotropy-tailored Au and Fe_3_O_4_. The UV-Vis absorbance spectra analyses of the multi-scale anisotropy-tailored hierarchical nanostructures [micro-isotropic + nano-isotropic (“M1+N1”), micro-isotropic + nano-anisotropic (“M1+N2”), micro-anisotropic + nano-isotropic (“M2+N1”), and micro-anisotropic + nano-anisotropic (“M2+N2”)] as well as each nanoscale anisotropy-tailored Au (N1 or N2) and microscale anisotropy-tailored Fe_3_O_4_ (M1 or M2) that comprise them.


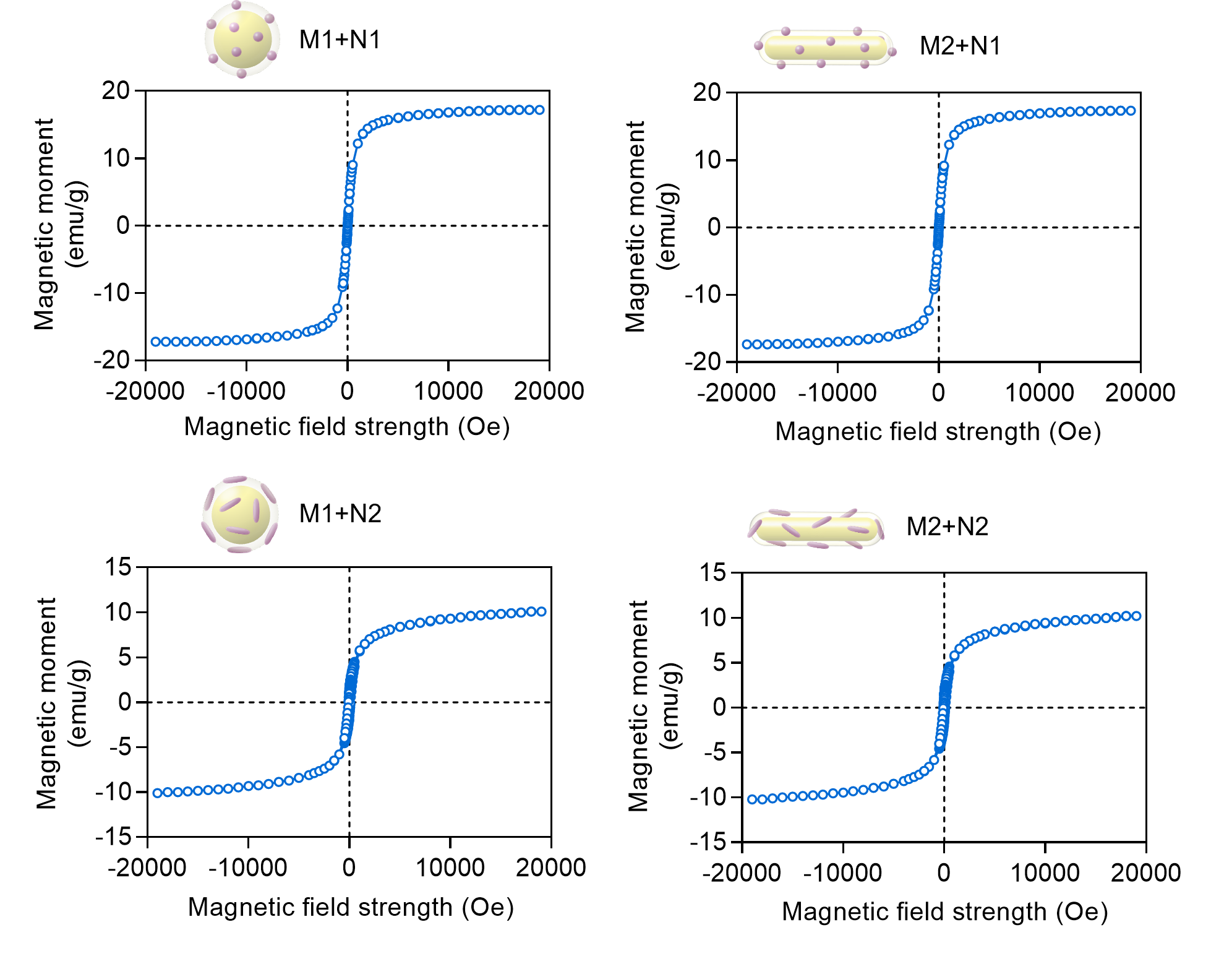


**Fig. S10.** Magnetic reversibility of multi-scale anisotropy-tailored hierarchical nanostructures is functional. The vibrating sample magnetometry (VSM) analysis of each of the multi-scale anisotropy-tailored hierarchical nanostructures [micro-isotropic + nano-isotropic (“M1+N1”), micro-isotropic + nano-anisotropic (“M1+N2”), micro-anisotropic + nano-isotropic (“M2+N1”), and micro-anisotropic + nano-anisotropic (“M2+N2”)]. The hysteresis loops of the magnetic moments were presented after normalization to the dry weights of the respective samples.


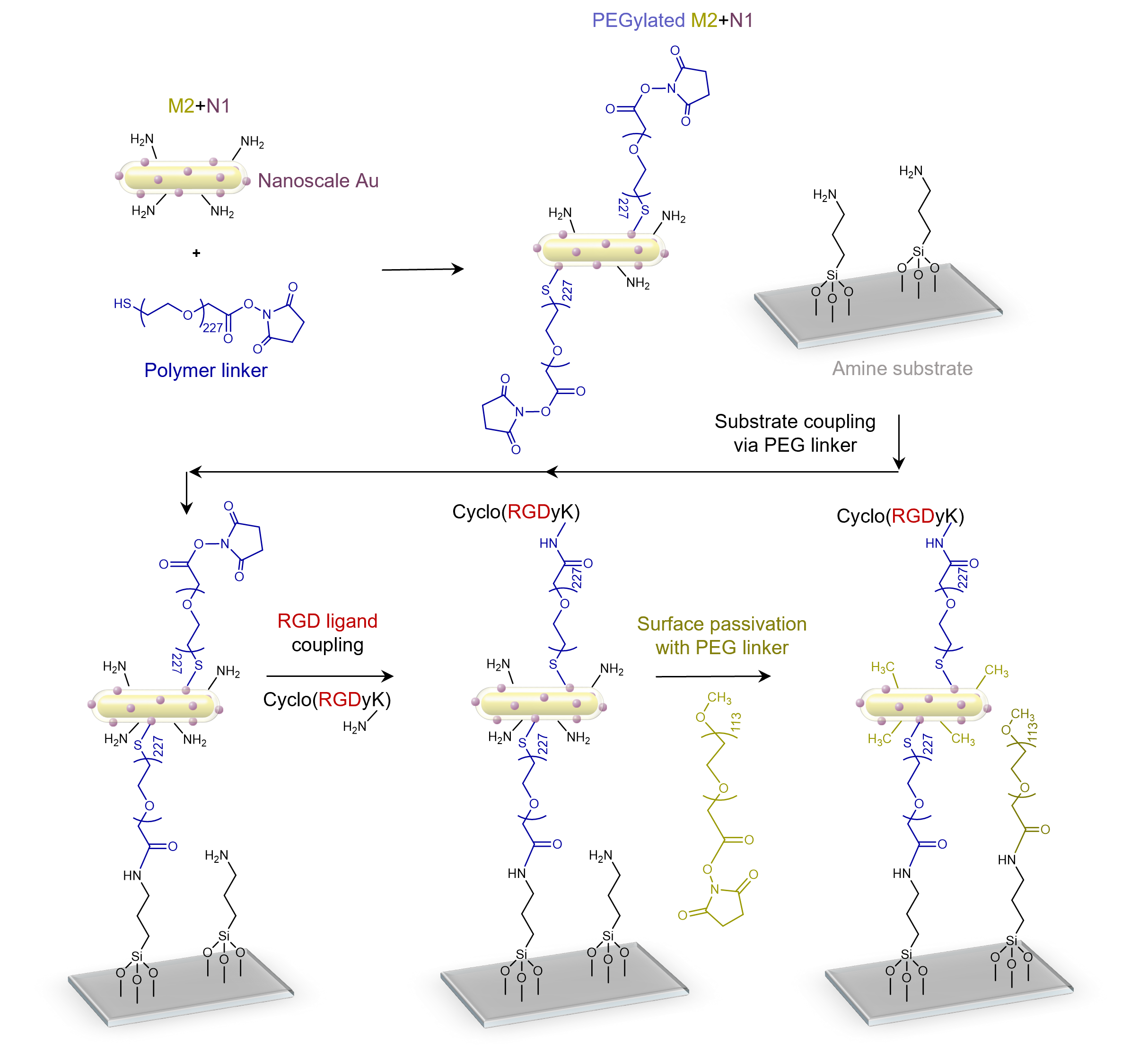


**Fig. S11.** Fabrication of a substrate with multi-scale ligand anisotropy-tailored hierarchical nanostructures. A schematic of the serial strategies involved in the fabrication of a substrate with multi-scale ligand anisotropy-tailored hierarchical nanostructures using polymer linkers for their axial magnetic manipulation and ligand coupling. The hierarchical nanostructure exhibiting micro-anisotropy and nano-isotropy (M2+N1) is illustrated as a representative model, which was first coupled with polymer linkers (PEGylated), coupled on the substrate surface, and then coupled with RGD ligand. Residual substrate surfaces not coupled with PEGylated hierarchical nanostructures were blocked via passivation.


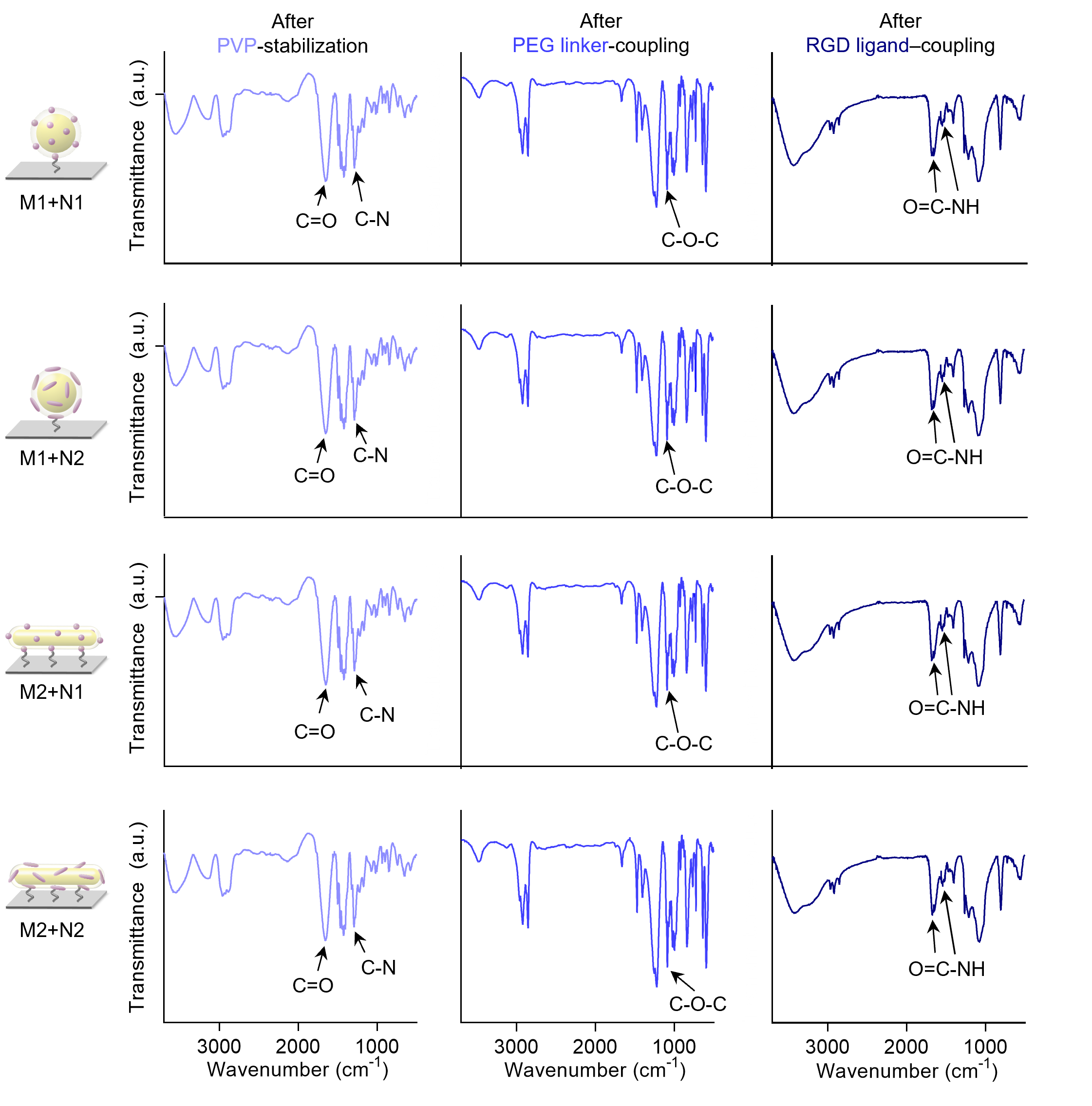


**Fig. S12.** Characterization of serial chemical bond changes during the development of the PEGylated ligand anisotropy-tailored hierarchical nanostructures. The serial Fourier transform infrared (FTIR) spectra analysis of the multi-scale anisotropy-tailored hierarchical nanostructures [micro-isotropic + nano-isotropic (“M1+N1”), micro-isotropic + nano-anisotropic (“M1+N2”), micro-anisotropic + nano-isotropic (“M2+N1”), and micro-anisotropic + nano-anisotropic (“M2+N2”)] after PVP stabilization, PEGylation, and ligand coating. Each characteristic absorption peak was labeled to their respective chemical bonds after serial chemical changes on their surfaces.


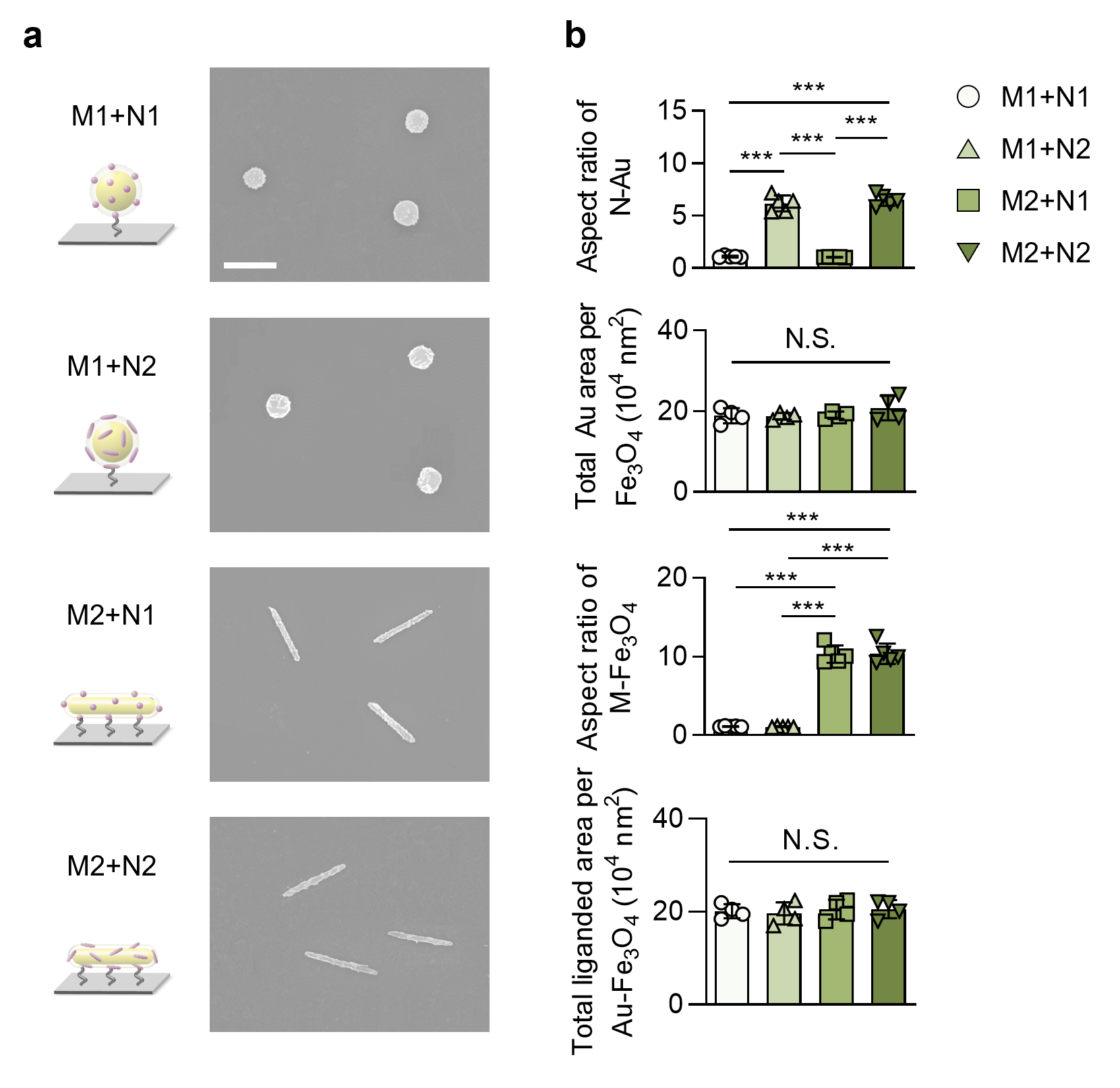


**Fig. S13.** Multi-scale ligand anisotropy-tailored hierarchical nanostructures exhibit equivalent surface areas on substrates. (a) Scanning electron microscopy (SEM) images of the substrates with hierarchical ligand nanostructures. (b) Corresponding calculation of the aspect ratio of N-Au, the total Au area per hierarchical nanostructure, the aspect ratio of M-Fe_3_O_4_, and the total liganded area per hierarchical nanostructure. Data are exhibited as the mean ± standard error (n=4). Asterisks assigned to the range of p values (***: p < 0.001) represent statistically significant differences. N.S. signifies that there is no statistically significant difference among the compared groups.


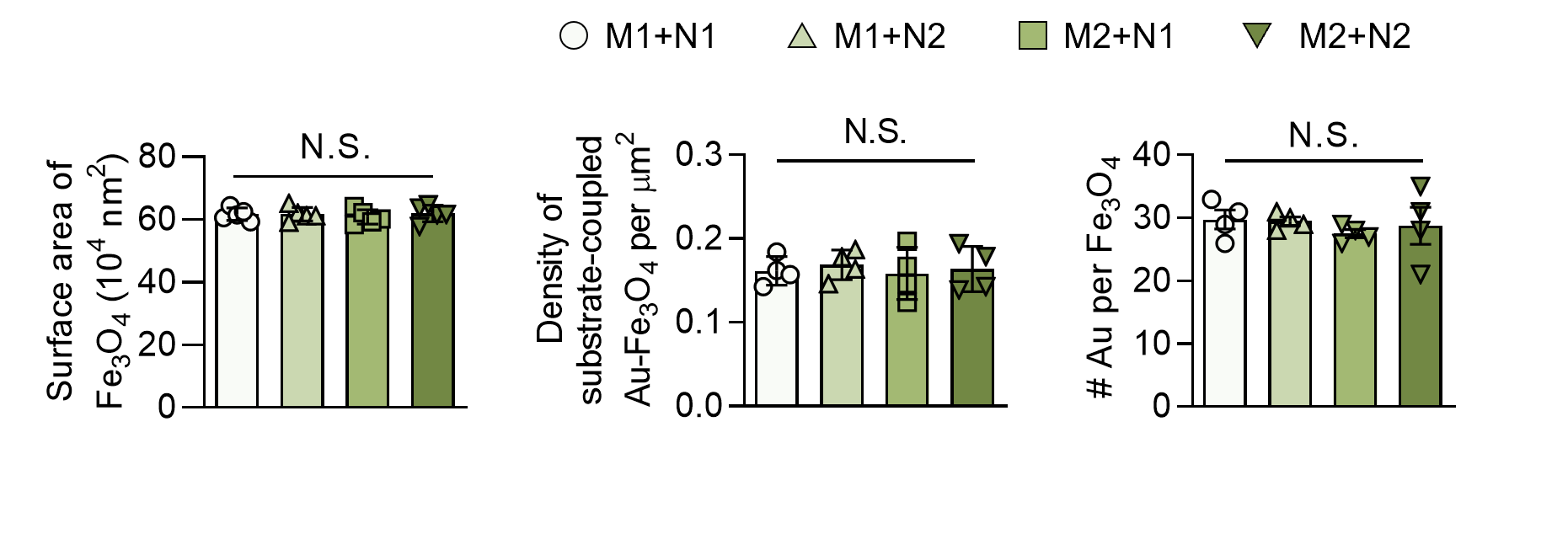


**Fig. S14.** Independently tailored hierarchical ligand nanostructures of equivalent surface areas exhibit constant substrate-coupled density. Calculations of the surface area of microscale anisotropy-tailored (isotropic or anisotropic) Fe_3_O_4_ (M1 or M2, respectively), the density, and the number of the nanoscale anisotropy-tailored (isotropic or anisotropic) Au (N1 or N2, respectively) in each of the multi-scale anisotropy-tailored hierarchical nanostructures (“M1+N1”, “M1+N2”, “M2+N1”, and “M2+N2” groups) shown in the scanning electron microscopy (SEM) images in Fig. S13a. Data are exhibited as the mean ± standard error (n=4). N.S. signifies that there is no statistically significant difference among the compared groups.


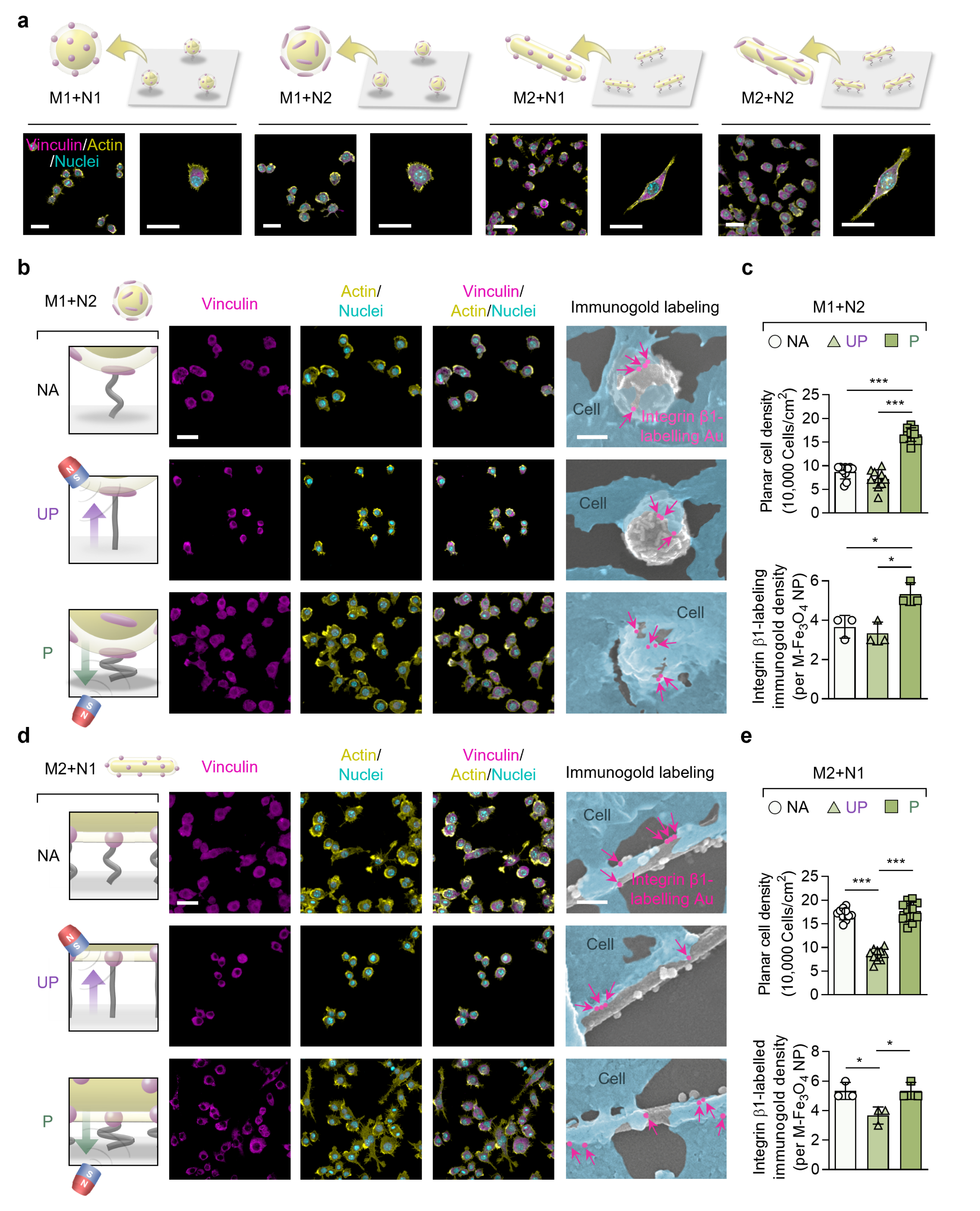


**Fig. S15.** Microscale ligand anisotropy dominates over nanoscale ligand anisotropy in activating dynamic macrophage adhesion. (a) Fluorescently immuno-stained images of vinculin with F-actin/nuclei and their overlay of adherent macrophages after 24 h of culturing on the micro-isotropic+nano-isotropic (“M1+N1”), micro-isotropic+nano-anisotropic (“M1+N2”), micro-anisotropic+nano-isotropic (“M2+N1”), or micro-anisotropic+nano-anisotropic (“M2+N2”) groups in a basal growth medium (scale bars: 20 µm). (b) Fluorescently immuno-stained images of vinculin with F-actin/nuclei and their overlay of adherent macrophages after 24 h of culturing on the “M1+N2” group in the “non-affected (NA)”, “unpressed (UP)”, or “pressed (P)” states with the scanning electron microscopy (SEM) images of integrin β1-labeling Au nanoparticles (pink) indicating macrophage (light blue) integrins recruited to each “M1+N2” group depending on their magnetic manipulation state [scale bars: 20 µm (confocal) and 200 nm (SEM)]. (c) Following calculations of the planar cell density and the density of integrin β1-labeling Au nanoparticles per each hierarchical nanostructure. (d) Fluorescently immuno-stained images of vinculin with F-actin/nuclei and their overlay of adherent macrophages after 24 h of culturing on the “M2+N1” group in the “NA”, “UP”, or “P” states with the SEM images of integrin β1-labeling Au nanoparticles (pink) indicating macrophage (light blue) integrins recruited to each “M2+N1” group depending on their magnetic manipulation state [scale bars: 20 µm (confocal) and 200 nm (SEM)]. (e) Following calculations of the planar cell density and the density of integrin β1-labeling Au nanoparticles per each hierarchical nanostructure. A permanent magnet (285 mT) was not placed in the “NA” state or placed either under or over the substrates to induce the “P” or “UP” state, respectively. Data are exhibited as the mean ± standard error (n=10). Asterisks assigned to the range of p values (*: p < 0.1; ***: p < 0.001) represent statistically significant differences.


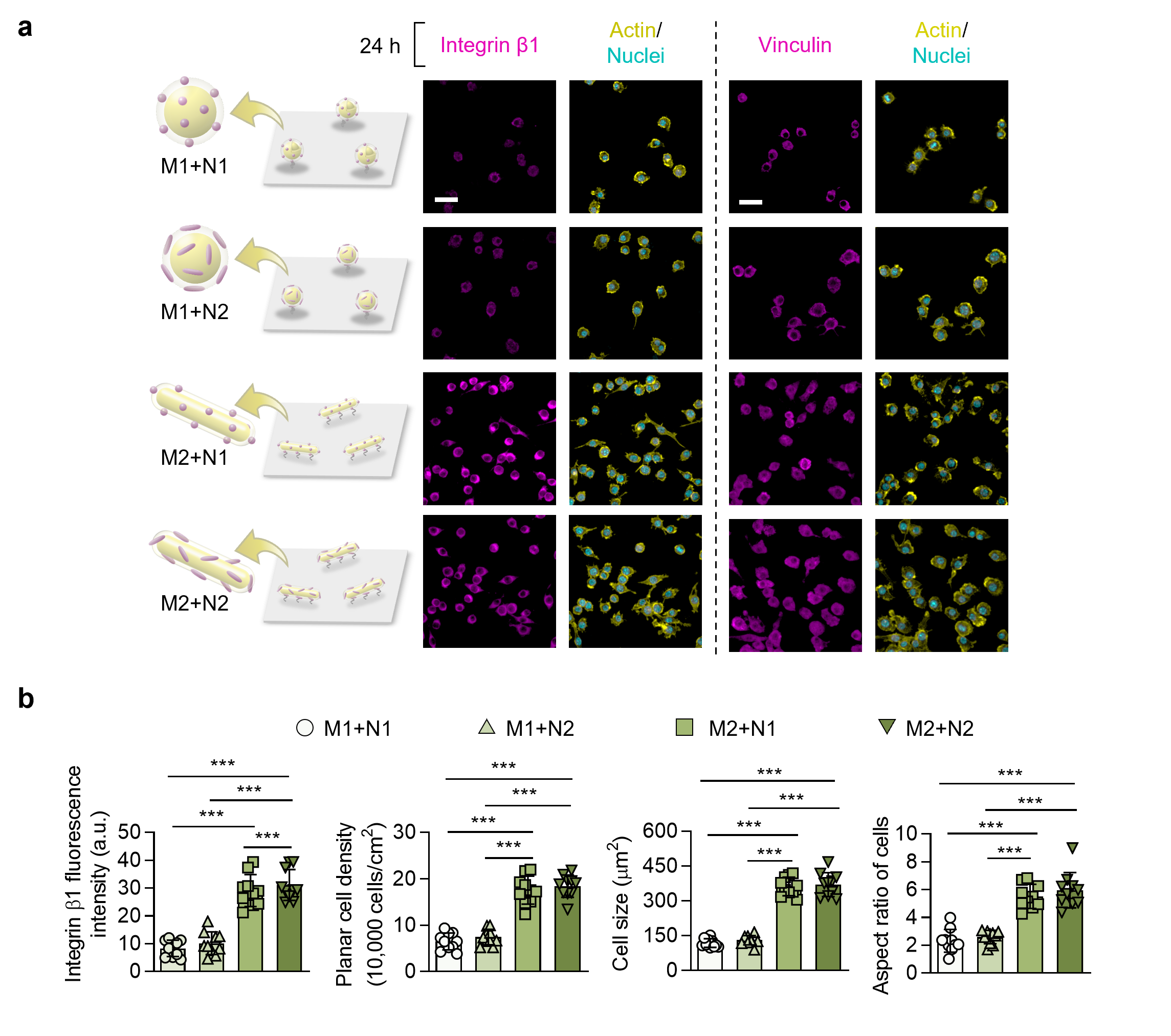


**Fig. S16.** Microscale ligand anisotropy dominates over the nanoscale in the activation of integrin recruitment for cell adhesion. (a) Fluorescently immuno-stained images of integrin β1 or vinculin with F-actin/nuclei and their overlay of adherent macrophages after 24 h of culturing on the micro-isotropic + nano-isotropic (“M1+N1”), micro-isotropic + nano-anisotropic (“M1+N2”), micro-anisotropic + nano-isotropic (“M2+N1”), or micro-anisotropic + nano-anisotropic (“M2+N2”) groups in a basal growth medium (scale bars: 20 µm). (b) Following calculations of the integrin β1 fluorescence intensity, planar cell density, cell size, and aspect ratio of adherent macrophages using the images shown in Fig. S15a and S16a. Data are exhibited as the mean ± standard error (n=10). Asterisks assigned to the range of p values (***: p < 0.001) represent statistically significant differences.


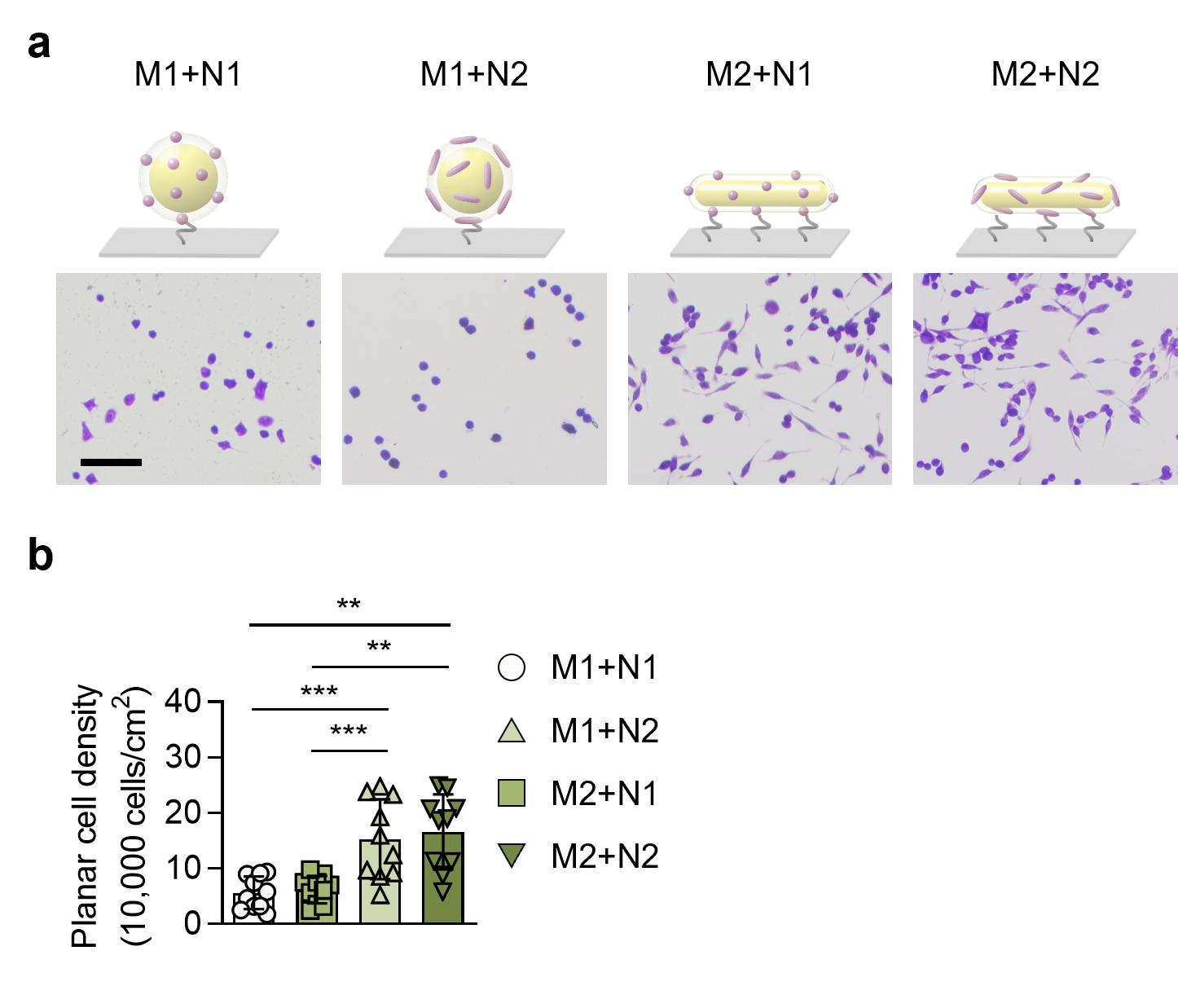


**Fig. S17.** Macrophage adhesion is dominantly regulated by microscale ligand anisotropy over nanoscale ligand anisotropy. (a) Crystal violet-stained images of adherent macrophages after 24 h of culturing on the micro-isotropic + nano-isotropic (“M1+N1”), micro-isotropic + nano-anisotropic (“M1+N2”), micro-anisotropic + nano-isotropic (“M2+N1”), or micro-anisotropic + nano-anisotropic (“M2+N2”) groups in a basal growth medium (scale bars: 100 µm). (b) Following calculations of the planar cell density of adherent macrophages. Data are exhibited as the mean ± standard error (n=10). Asterisks assigned to the range of p values (**: p < 0.01; ***: p < 0.001) represent statistically significant differences.


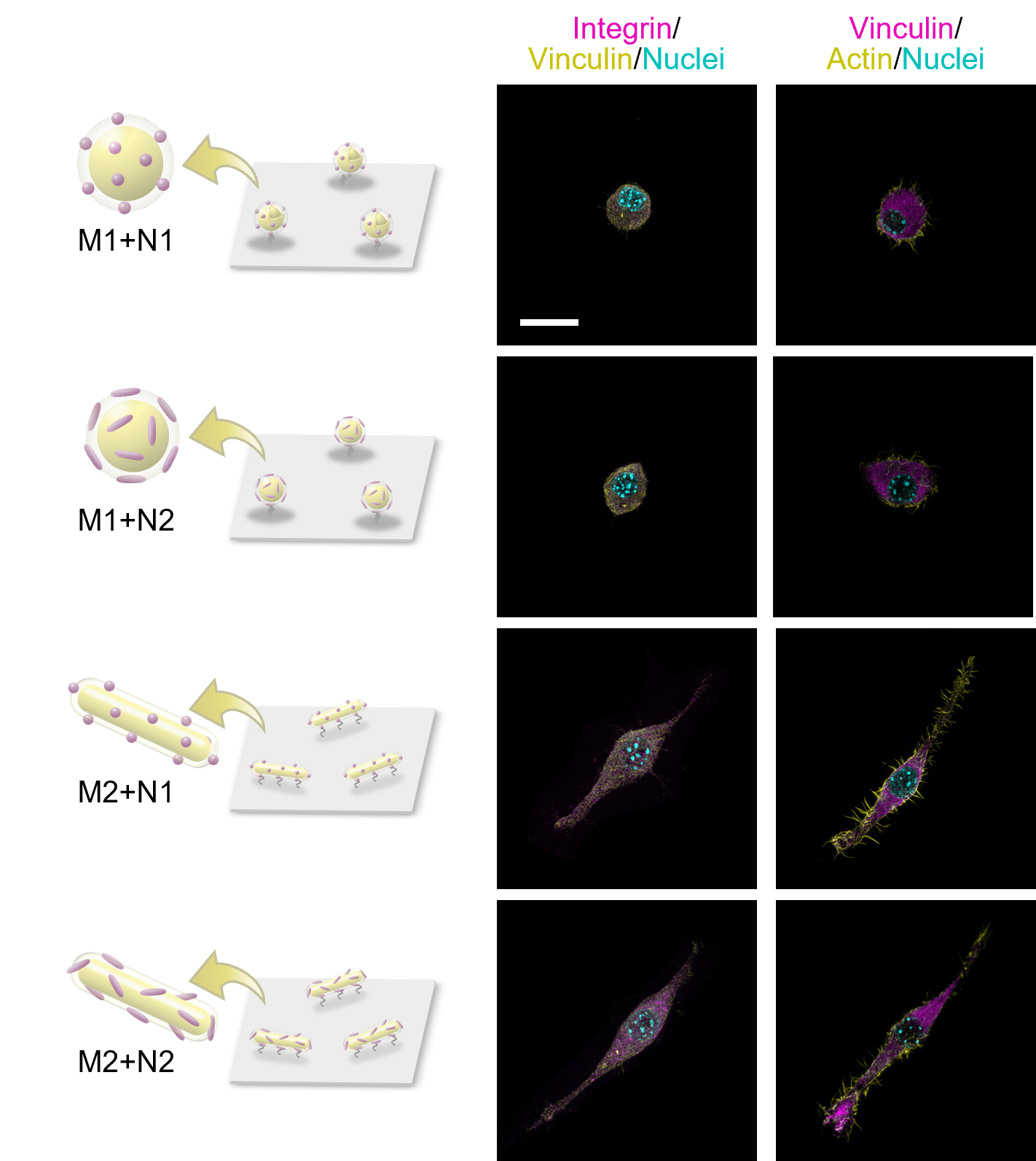


**Fig. S18.** Microscale anisotropy-mediated integrin recruitment is associated with macrophage adhesion. Fluorescently immuno-stained overlay images of integrin/vinculin/nuclei or vinculin/F-actin/nuclei of adherent macrophages after 24 h of culturing on the micro-isotropic + nano-isotropic (“M1+N1”), micro-isotropic + nano-anisotropic (“M1+N2”), micro-anisotropic + nano-isotropic (“M2+N1”), or micro-anisotropic + nano-anisotropic (“M2+N2”) groups in a basal growth medium (scale bars: 20 µm).


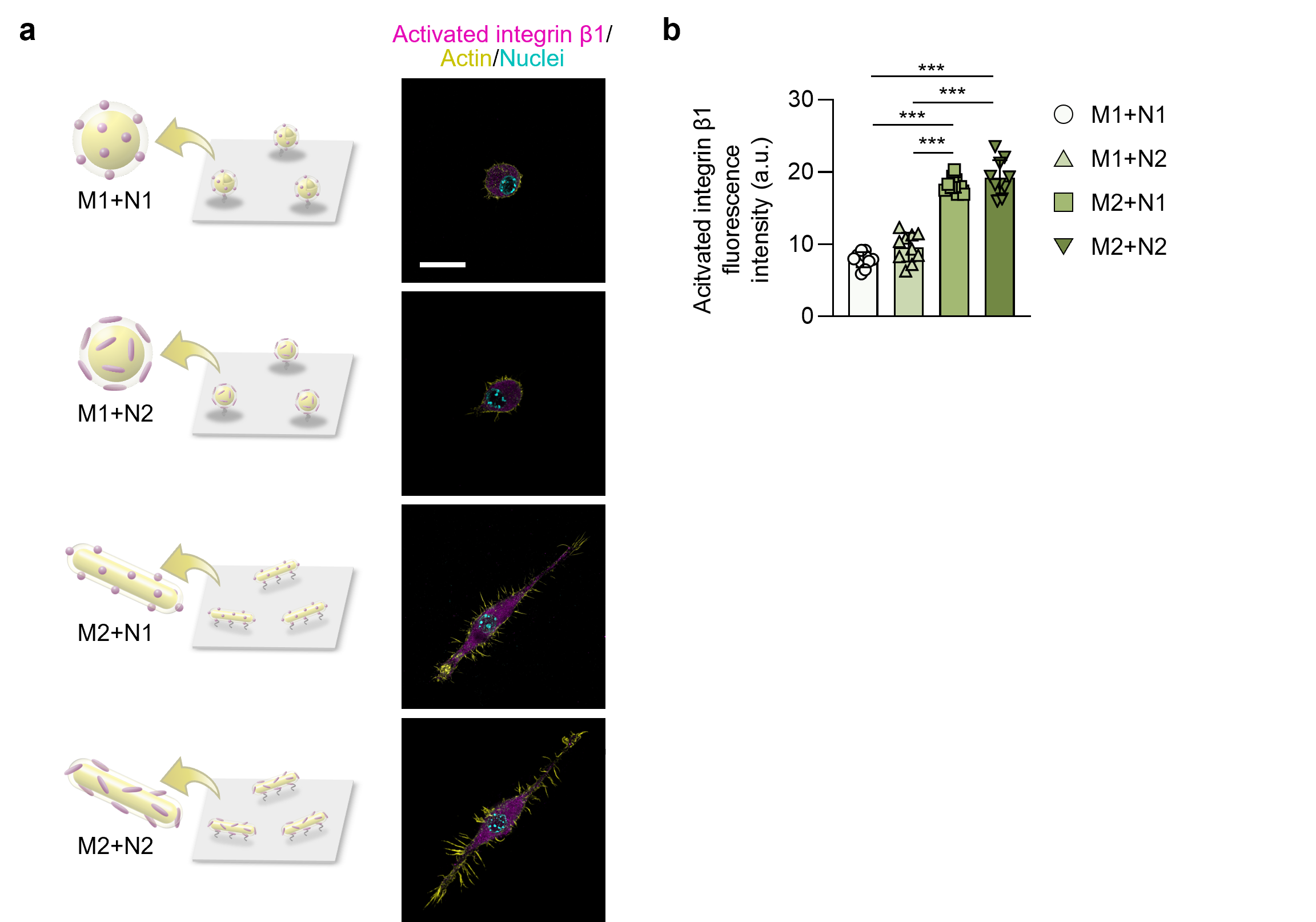


**Fig. S19.** Microscale ligand anisotropy dominates over nanoscale ligand anisotropy in activating integrin β1. (a) Fluorescently immuno-stained overlay images of activated integrin β1/F-actin/nuclei of adherent macrophages after 24 h of culturing on the micro-isotropic + nano-isotropic (“M1+N1”), micro-isotropic + nano-anisotropic (“M1+N2”), micro-anisotropic + nano-isotropic (“M2+N1”), or micro-anisotropic + nano-anisotropic (“M2+N2”) groups in a basal growth medium (scale bars: 20 µm). (b) Following fluorescence intensity of the activated integrin β1 of adherent macrophages. Data are exhibited as the mean ± standard error (n=10). Asterisks assigned to the range of p values (***: p < 0.001) represent statistically significant differences.


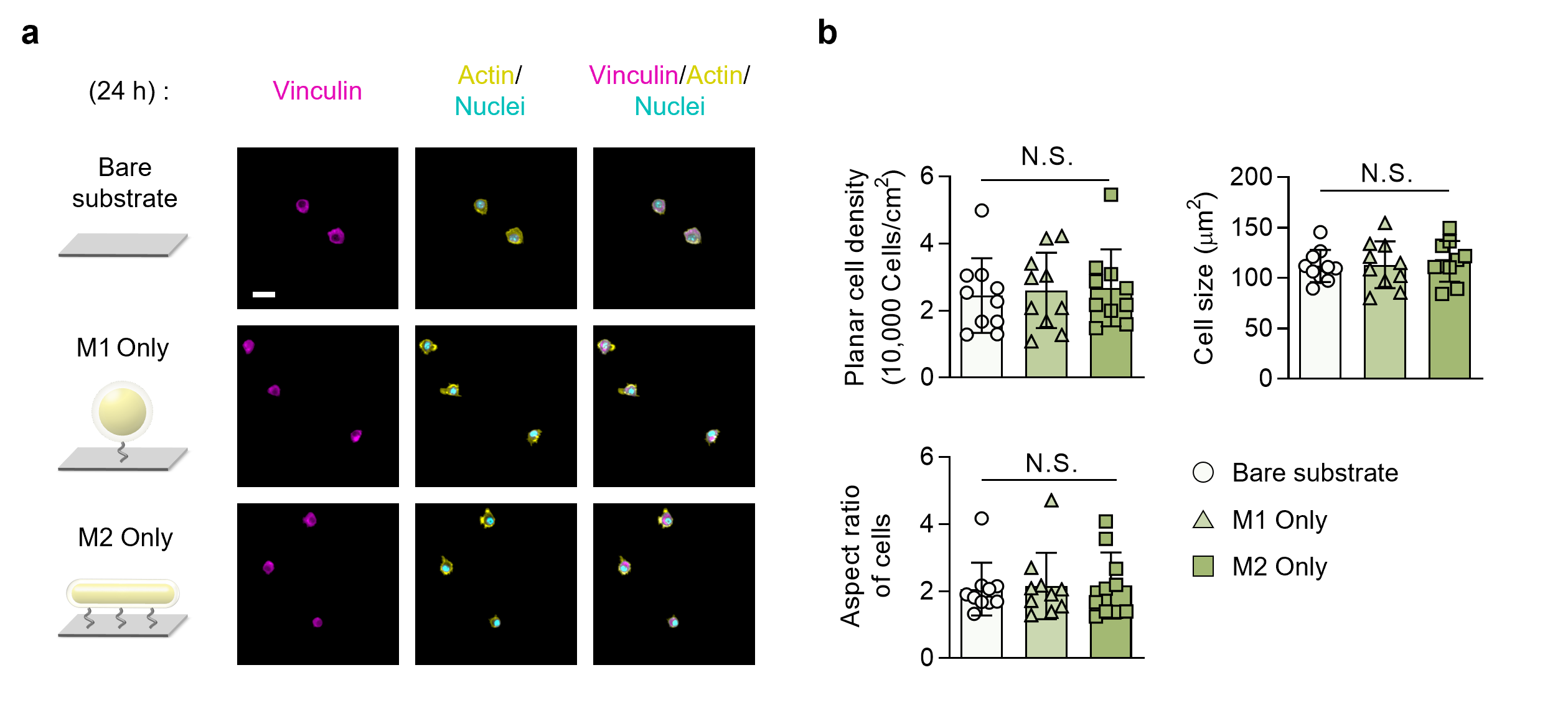


**Fig. S20.** Tailoring the microscale anisotropy alone without Au nanoparticles does not regulate macrophage adhesion. (a) Fluorescently immuno-stained images of vinculin with F-actin/nuclei and their overlay of adherent macrophages after 24 h of culturing on the “bare”, “micro-isotropic Fe_3_O_4_ (M1) only”, and “micro-anisotropic Fe_3_O_4_ (M2) only” groups in a basal growth medium (scale bar: 20 µm). (b) Following calculations of the planar cell density, cell size, and aspect ratio of adherent macrophages. Data are exhibited as the mean ± standard error (n=10). N.S. signifies that there is no statistically significant difference among the compared groups.


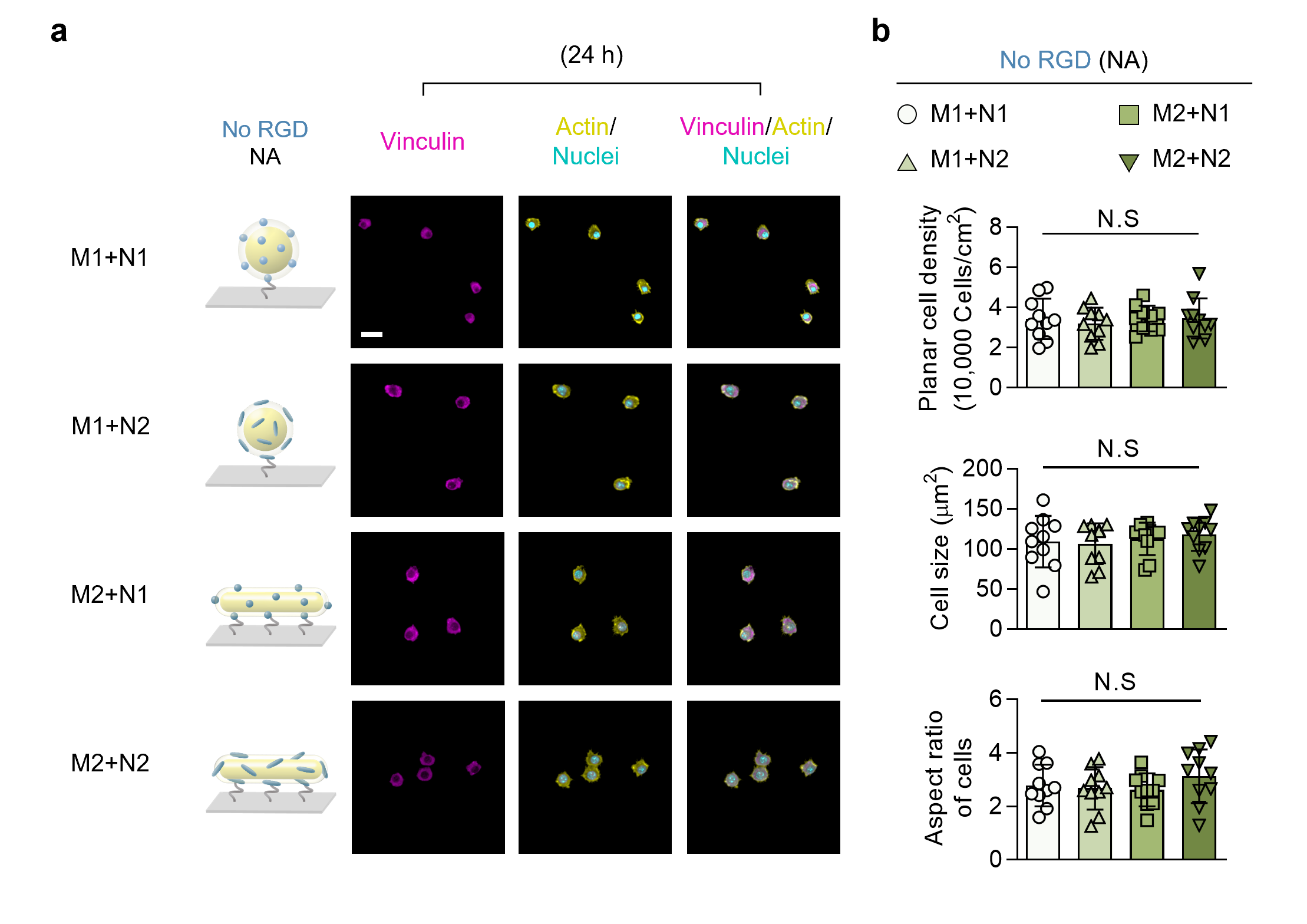


**Fig. S21.** Tailoring multi-scale anisotropy without ligand coating on Au nanoparticles does not efficiently regulate macrophage adhesion. (a) Fluorescently immuno-stained images of vinculin with F-actin/nuclei and their overlay of adherent macrophages after 24 h of culturing on the micro-isotropic + nano-isotropic (“M1+N1”), micro-isotropic + nano-anisotropic (“M1+N2”), micro-anisotropic + nano-isotropic (“M2+N1”), and micro-anisotropic + nano-anisotropic (“M2+N2”) groups without any ligand coating in a basal growth medium (scale bar: 20 µm). (b) Following calculations of the planar cell density, cell size, and aspect ratio of adherent macrophages. Data are exhibited as the mean ± standard error (n=10). N.S. signifies that there is no statistically significant difference among the compared groups.


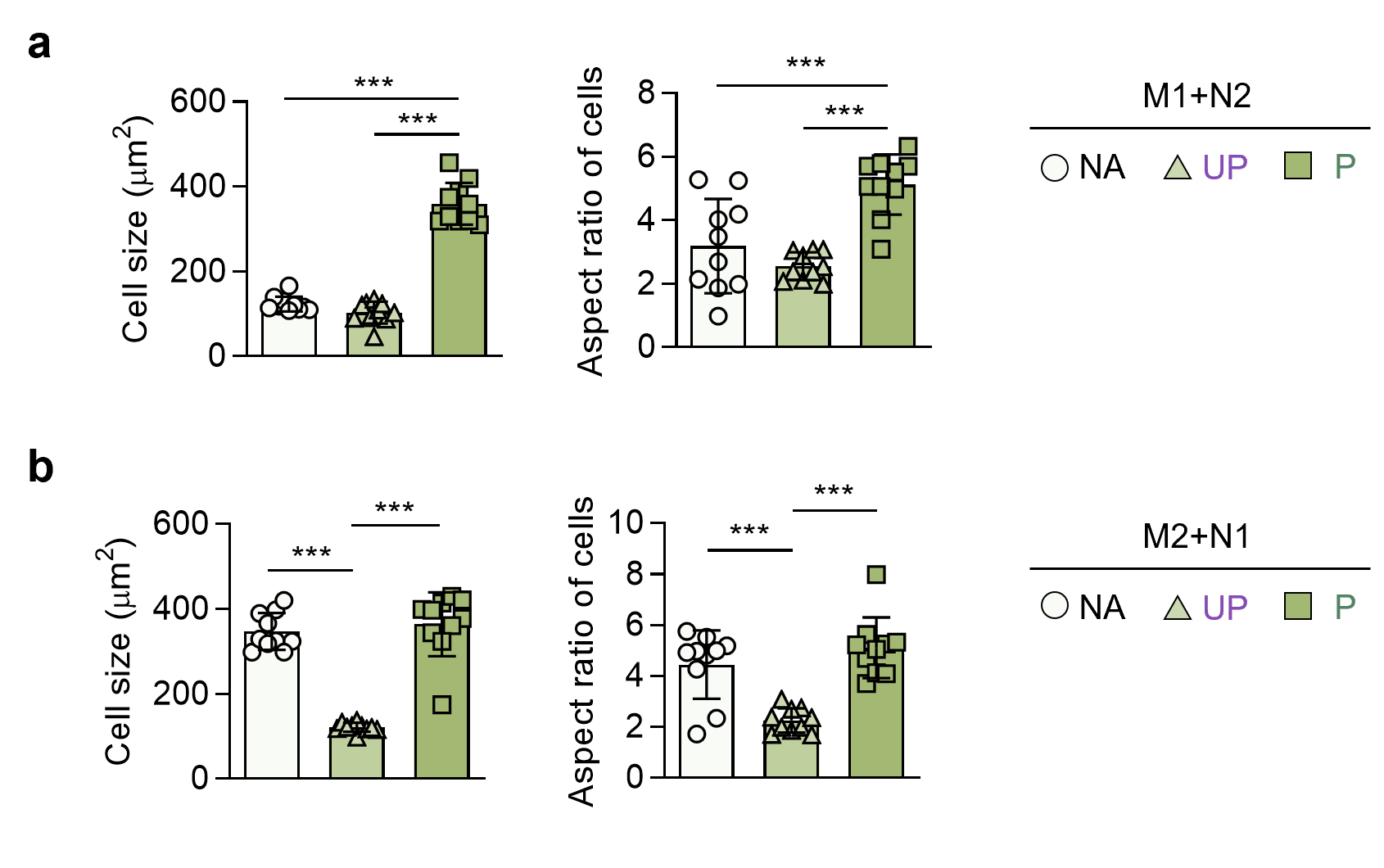


**Fig. S22.** Axial manipulation of hierarchical ligand nanostructures dynamically regulates the size and aspect ratio of adherent macrophages. Calculations of the cell size and aspect ratio of adherent macrophages shown in the fluorescently immuno-stained images in (a) Fig. S15b and (b) Fig. S15d. The macrophages were cultured for 24 h either on the micro-isotropic + nano-anisotropic (“M1+N2”) group or on the micro-anisotropic + nano-isotropic (“M2+N1”) group in the “non-affected (NA)”, “unpressed (UP)”, or “pressed (P)” state. The magnet was not placed in the “NA” state or placed either under or over the substrates to induce the “P” or “UP” state, respectively. Data are exhibited as the mean ± standard error (n=10). Asterisks assigned to the range of p values (***: p < 0.001) represent statistically significant differences.


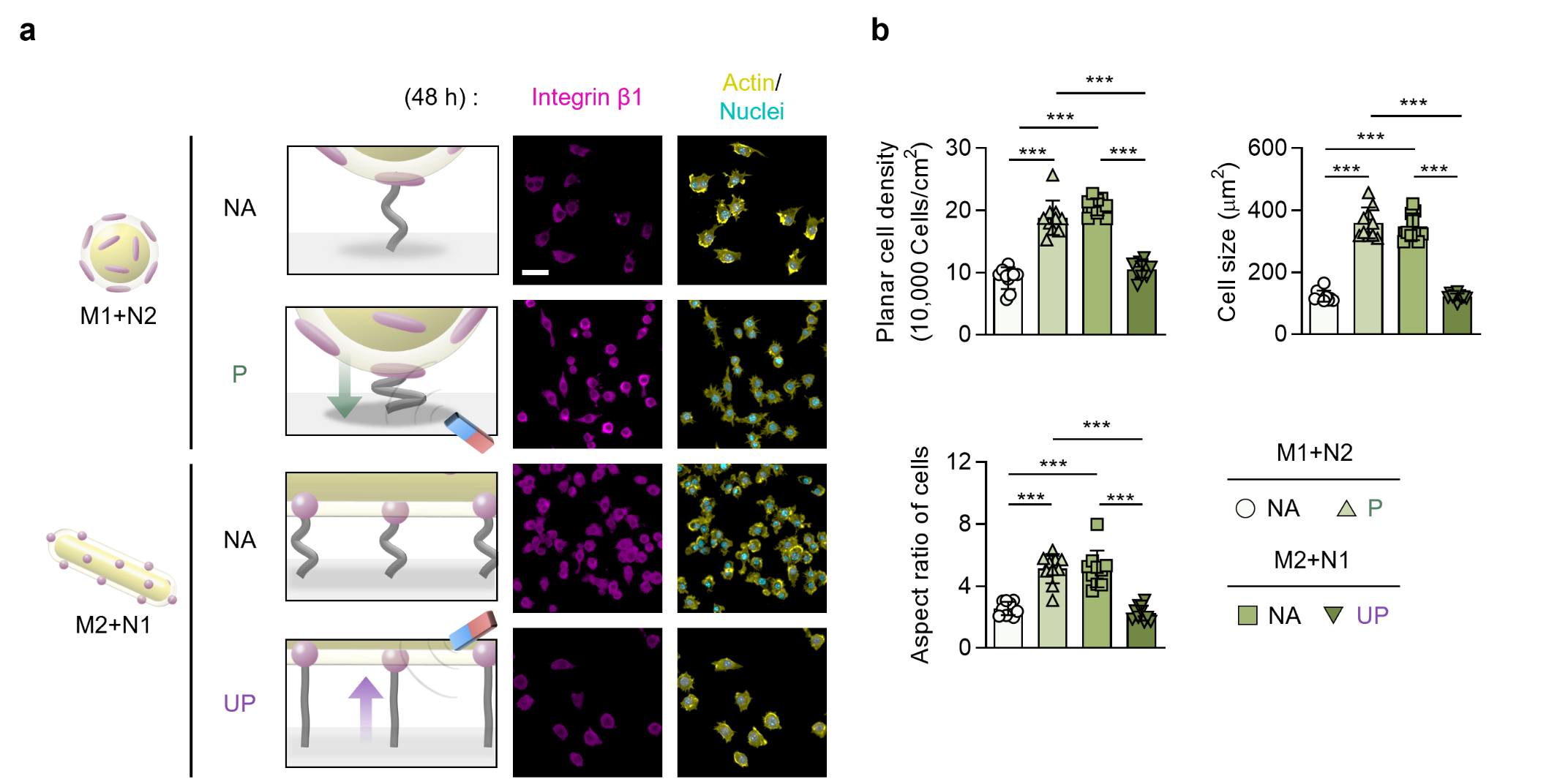


**Fig. S23.** Axial manipulation of hierarchical ligand nanostructures effectively switches multi-scale-ligand-anisotropy-regulated macrophage integrin recruitment and adhesion. (a) Fluorescently immuno-stained images of integrin ꞵ1 with F-actin/nuclei and their overlay of adherent macrophages after 48 h of culturing either on the micro-isotropic + nano-anisotropic (“M1+N2”) group in the “non-affected (NA)” or “pressed (P)” state, or on the micro-anisotropic + nano-isotropic (“M2+N1”) group in the “NA” or “unpressed (UP)” states in a basal growth medium (scale bar: 20 µm). (b) Following calculations of the planar cell density, cell size, and aspect ratio of adherent macrophages. The magnet was not placed in the “NA” state or placed either under or over the substrates to induce the “P” or “UP” state, respectively. Data are exhibited as the mean ± standard error (n=10). Asterisks assigned to the range of p values (***: p < 0.001) represent statistically significant differences.


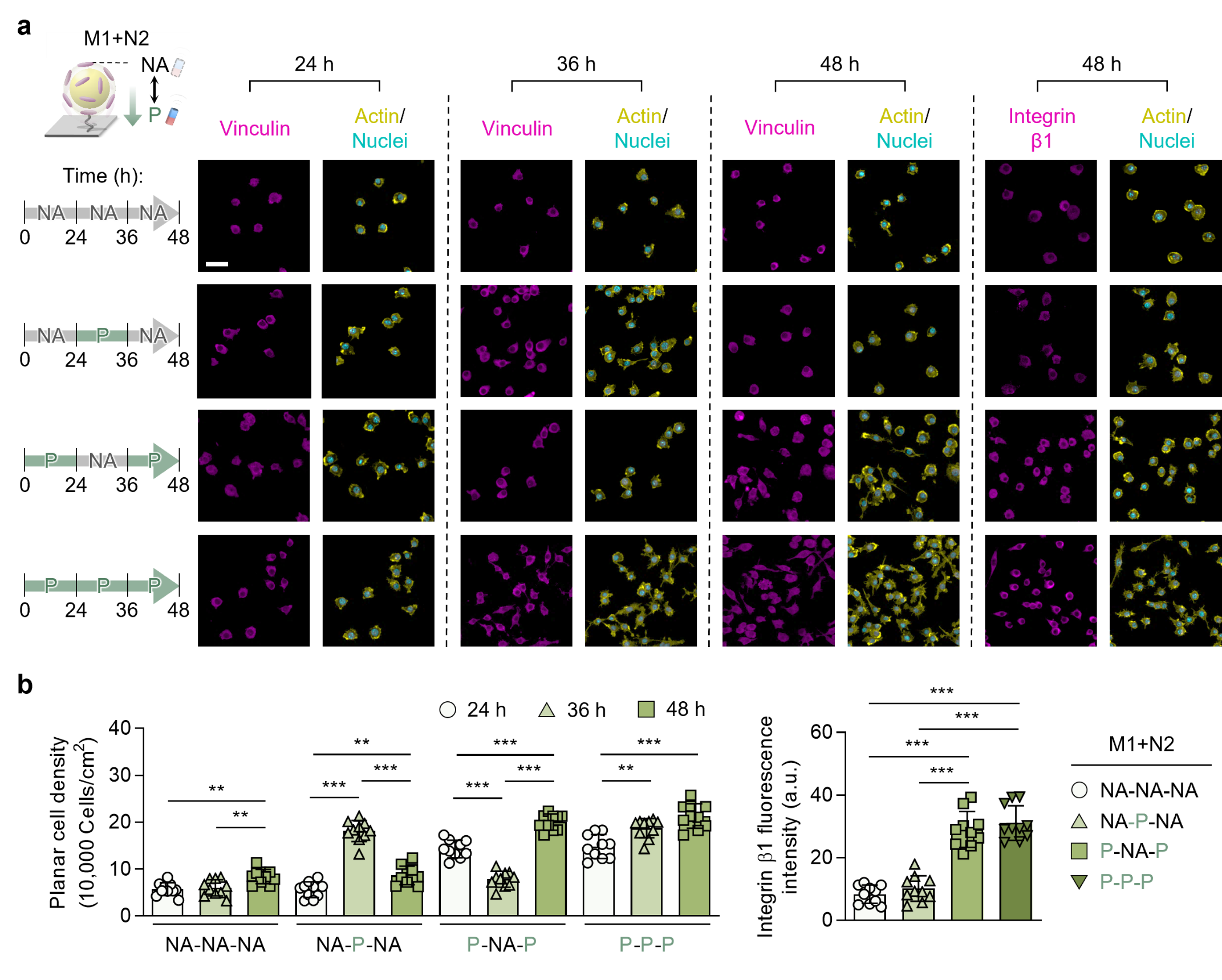


**Fig. S24.** Substrate-proximate manipulation cyclically activates microscale ligand isotropy-suppressed macrophage adhesion. (a) Fluorescently immuno-stained images of vinculin or integrin β1 with F-actin/nuclei of adherent macrophages after 24, 36, or 48 h of culturing on the micro-isotropic + nano-anisotropic (“M1+N2”) group subjected to reversible downward manipulation (scale bar: 20 µm). (b) Corresponding calculations of the planar cell density and integrin β1 fluorescence intensity of adherent macrophages. For cyclic substrate-proximate downward manipulation, the placement and displacement of the permanent magnet under the substrate were either switched or maintained every 12 h (after 24 h of culturing) up to 48 h (“NA-NA-NA”, “NA-P-NA”, “P-NA-P”, and “P-P-P”). Data are exhibited as the mean ± standard error (n=10). Asterisks assigned to the range of p values (**: p < 0.01; ***: p < 0.001) represent statistically significant differences.


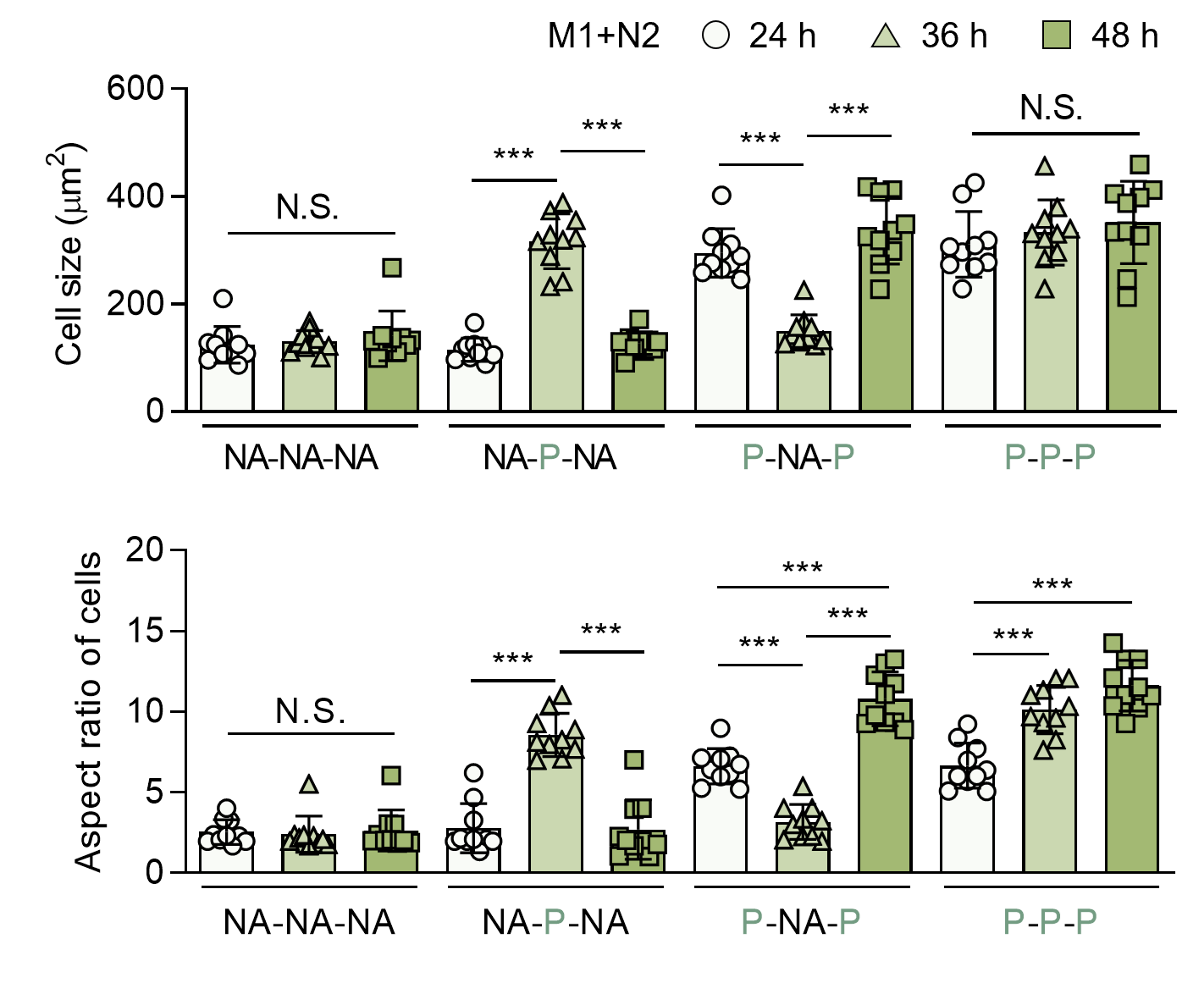


**Fig. S25.** Substrate-proximate manipulation cyclically supports macrophage adhesion on the micro-isotropic + nano-anisotropic ligand nanostructure. Calculations of the cell size and aspect ratio of the adherent macrophages shown in the fluorescently immuno-stained images in Fig. S24a. The macrophages were cultured for 24, 36, or 48 h on the “M1+N2” group subjected to reversible downward manipulation, in which the placement [“Pressed (P)”] and displacement [“Non-affected (NA)”] of the magnet under the substrate were either switched or maintained every 12 h (after 24 h of culturing) up to 48 h (“NA-NA-NA”, “NA-P-NA”, “P-NA-P”, and “P-P-P”). Data are exhibited as the mean ± standard error (n=10). Asterisks assigned to the range of p values (***: p < 0.001) represent statistically significant differences. N.S. signifies that there is no statistically significant difference among the compared groups.


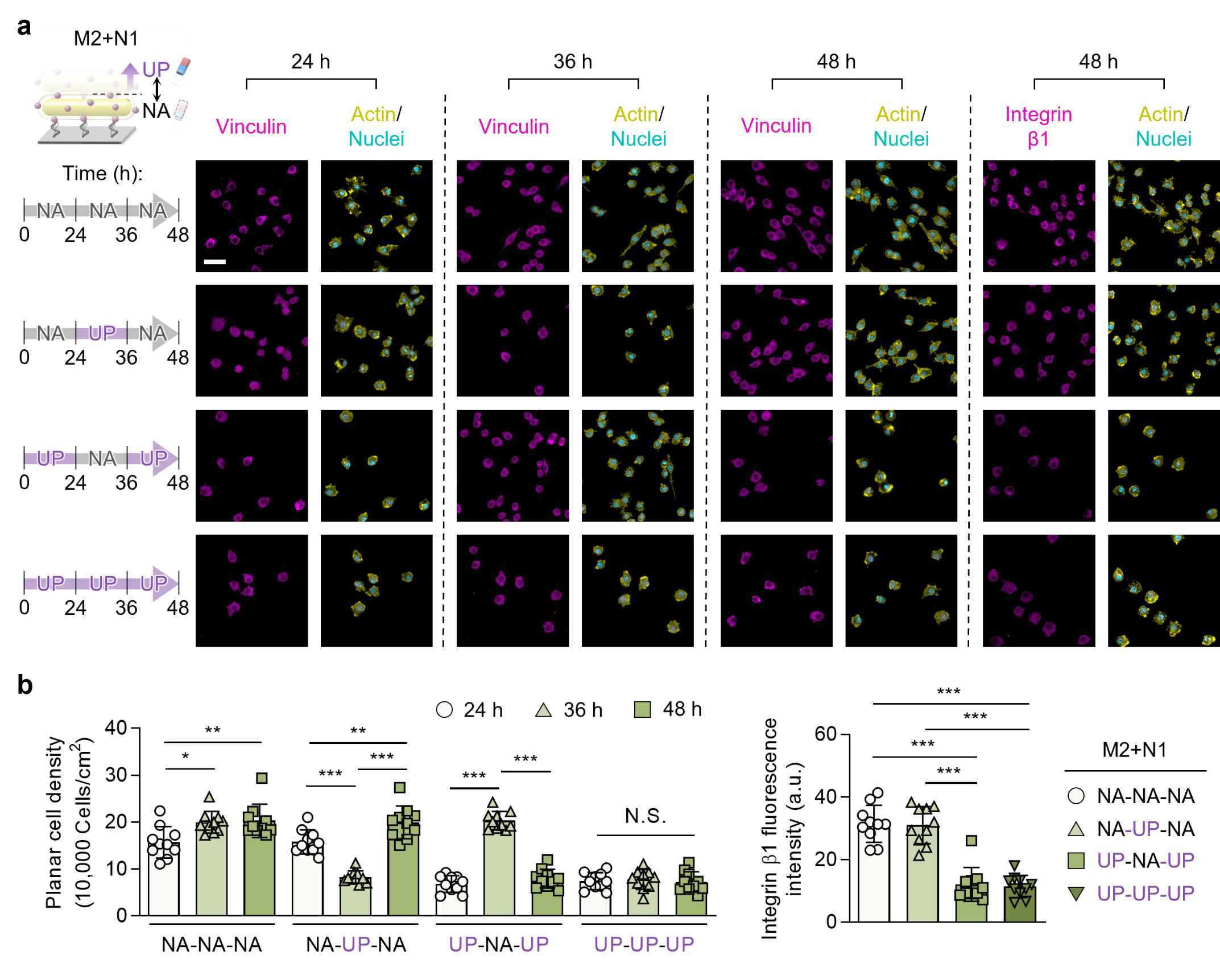


**Fig. S26.** Substrate-distant manipulation cyclically deactivates microscale ligand anisotropy-mediated macrophage adhesion. (a) Fluorescently immuno-stained images of vinculin or integrin β1 with F-actin/nuclei of adherent macrophages after 24, 36, or 48 h of culturing on the micro-anisotropic + nano-isotropic “M2+N1” group subjected to reversible upward manipulation (scale bar: 20 µm). (b) Corresponding calculations of the planar cell density and integrin β1 fluorescence intensity of the adherent macrophages. For the reversible substrate-distant upward manipulation, the placement and displacement of the magnet over the substrate were either switched or maintained every 12 h (after 24 h of culturing) up to 48 h (“NA-NA-NA”, “NA-UP-NA”, “UP-NA-UP”, and “UP-UP-UP”). Data are exhibited as the mean ± standard error (n=10). Asterisks assigned to the range of p values (*: p < 0.1; **: p < 0.01; ***: p < 0.001) represent statistically significant differences. N.S. signifies that there is no statistically significant difference among the compared groups.


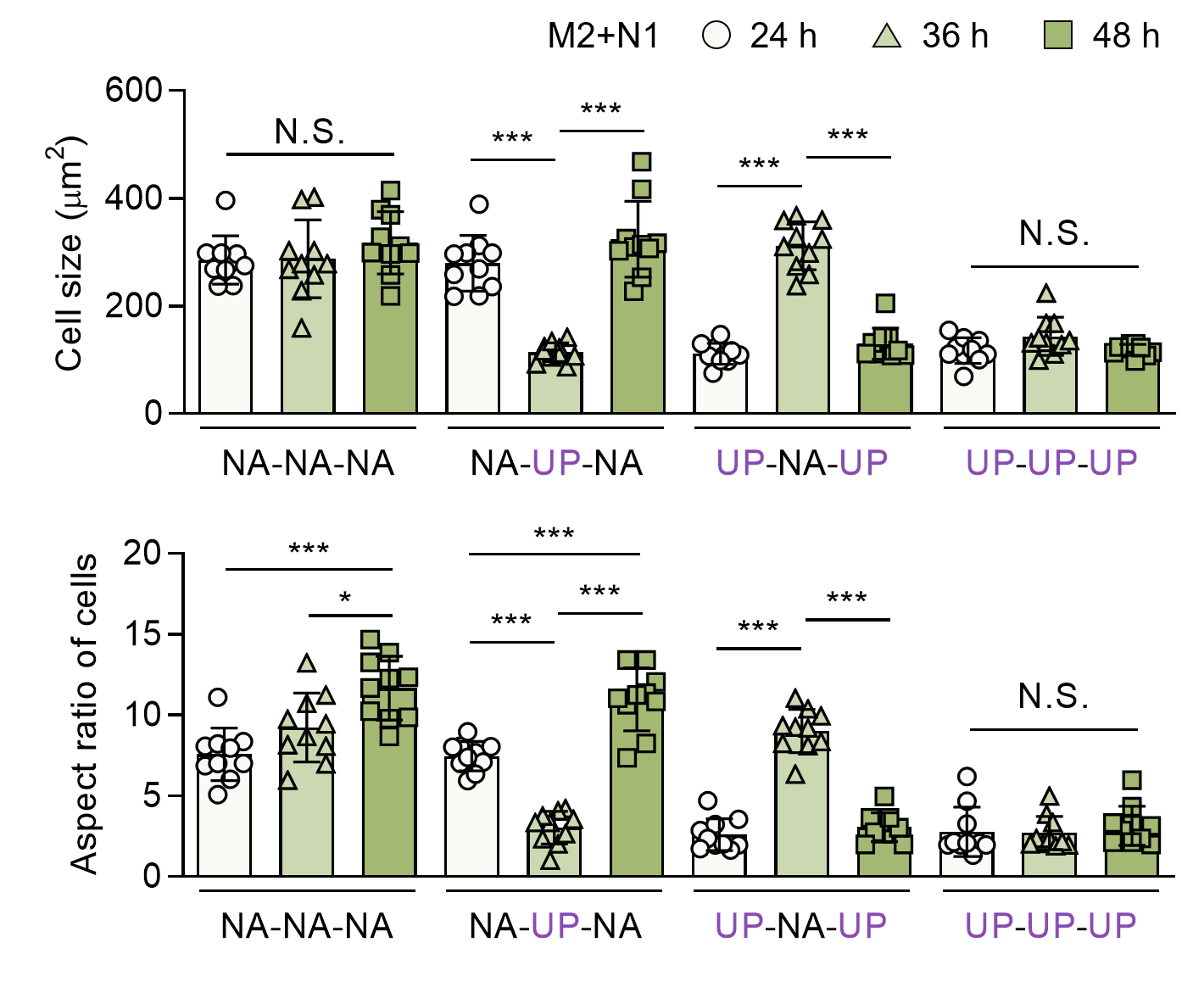


**Fig. S27.** Substrate-distant manipulation cyclically suppresses macrophage adhesion on the micro-anisotropic + nano-isotropic ligand nanostructure. Calculations of the cell size and aspect ratio of the adherent macrophages shown in the fluorescently immuno-stained images in Fig. S26a. The macrophages were cultured for 24, 36, or 48 h on the “M2+N1” group subjected to reversible upward manipulation, in which the placement [“Unpressed (UP)”] and displacement [“Non-affected (NA)”] of the magnet over the substrate were either switched or maintained every 12 h (after 24 h of culturing) up to 48 h (“NA-NA-NA”, “NA-UP-NA”, “UP-NA-UP”, and “UP-UP-UP”). Data are exhibited as the mean ± standard error (n=10). Asterisks assigned to the range of p values (*: p < 0.1; ***: p < 0.001) represent statistically significant differences. N.S. signifies that there is no statistically significant difference among the compared groups.


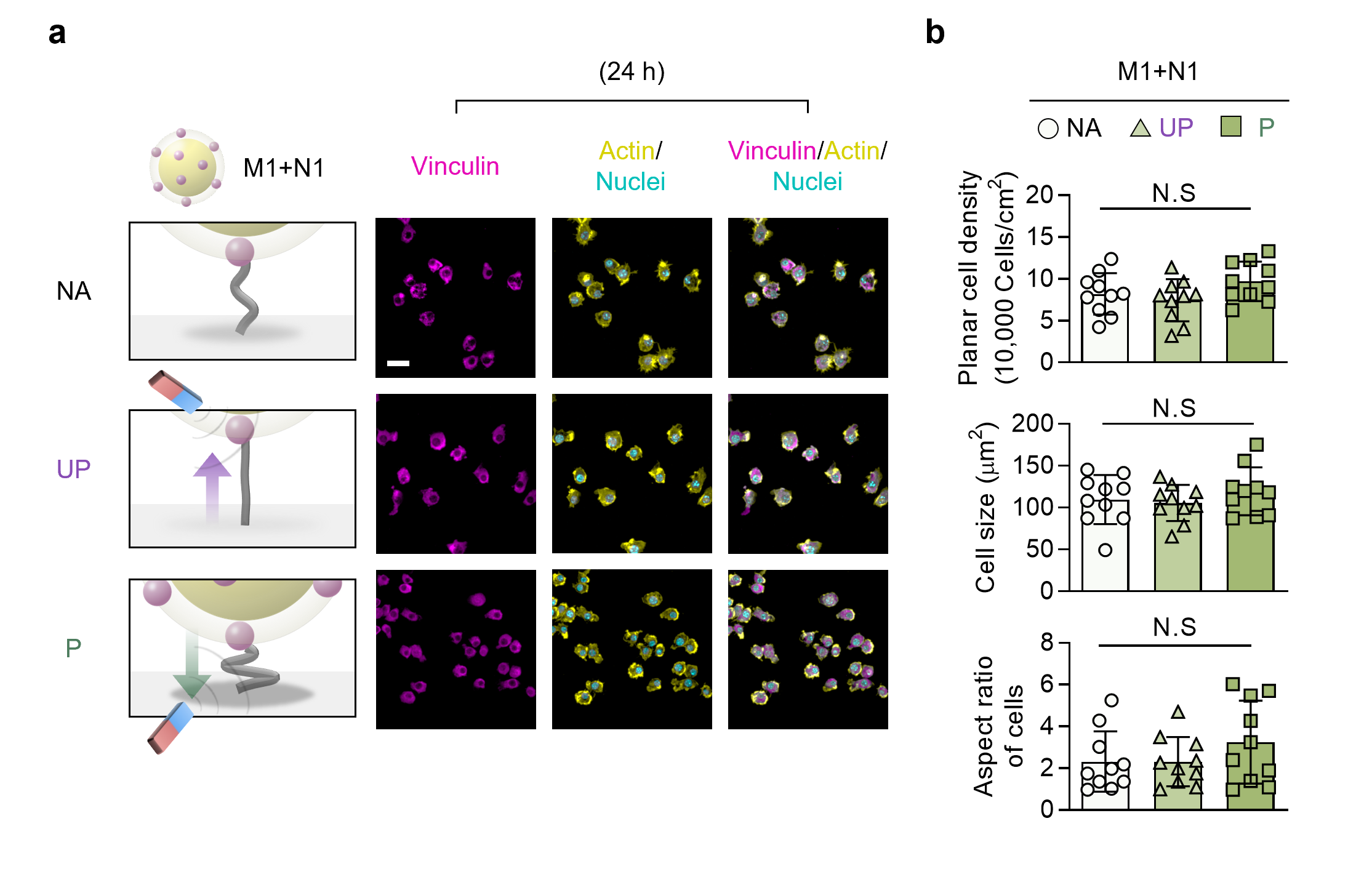


**Fig. S28.** Axial manipulation of dual (both nanoscale and microscale) isotropy of ligands is inefficient in modulating macrophage adhesion. (a) Fluorescently immuno-stained images of vinculin with F-actin/nuclei and their overlay of adherent macrophages after 24 h of culturing on the micro-isotropic + nano-isotropic (“M1+N1”) group in the “non-affected (NA)”, “unpressed (UP)”, and “pressed (P)” states in a basal growth medium (scale bar: 20 µm). (b) Following calculations of the planar cell density, cell size, and aspect ratio of adherent macrophages. The magnet was not placed in the “NA” state or placed either under or over the substrates to induce the “P” or “UP” state, respectively. Data are exhibited as the mean ± standard error (n=10). N.S. signifies that there is no statistically significant difference among the compared groups.


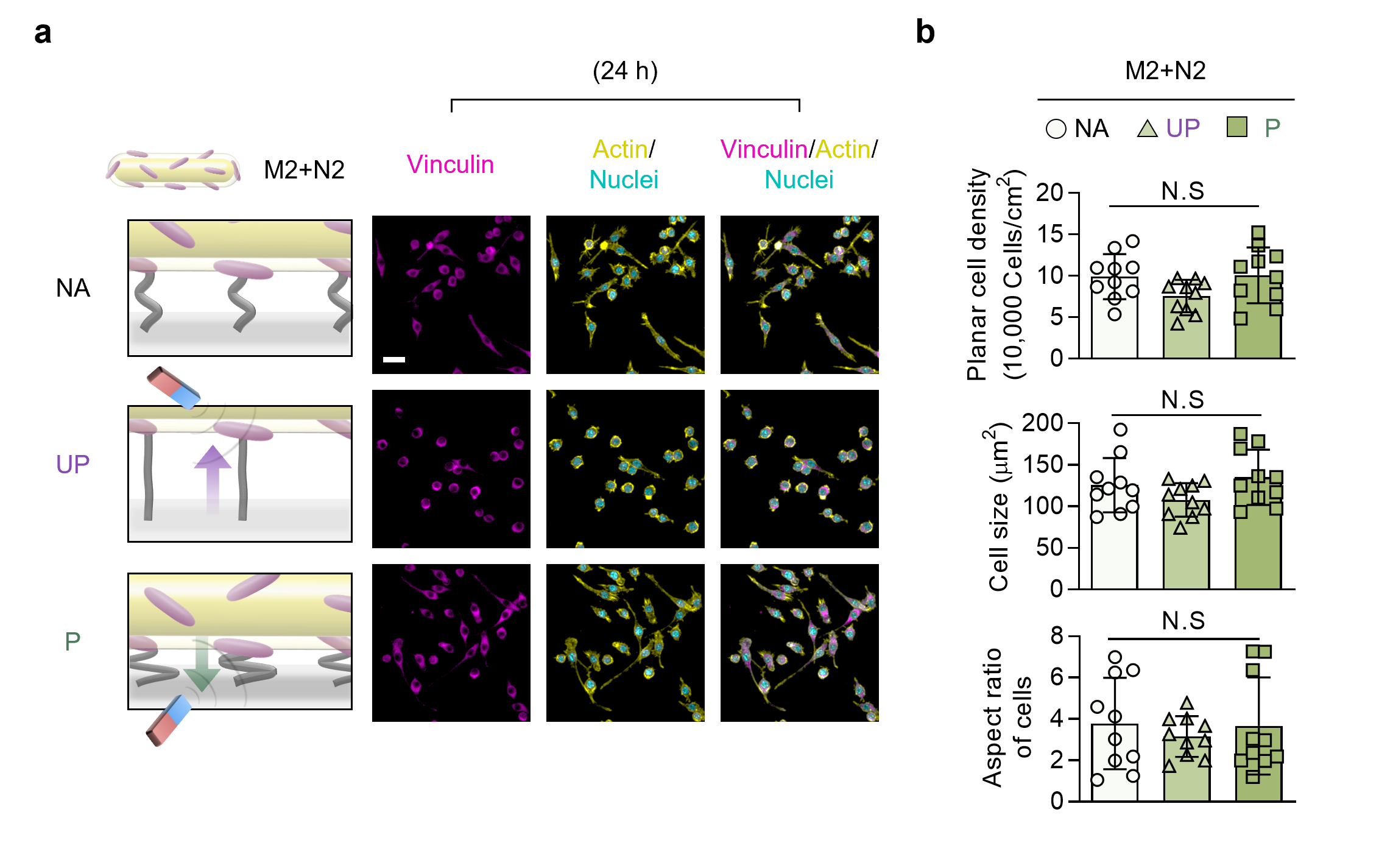


**Fig. S29.** Axial manipulation of dual (both nanoscale and microscale) anisotropy of ligands does not effectively modulate macrophage adhesion. (a) Fluorescently immuno-stained images of vinculin with F-actin/nuclei and their overlay of adherent macrophages after 24 h of culturing on the micro-anisotropic + nano-anisotropic (“M2+N2”) group in the “non-affected (NA)”, “unpressed (UP)”, and “pressed (P)” states in a basal growth medium (scale bar: 20 µm). (b) Following calculations of the planar cell density, cell size, and aspect ratio of adherent macrophages. The magnet was not placed in the “NA” state or placed either under or over the substrates to induce the “P” or “UP” state, respectively. Data are exhibited as the mean ± standard error (n=10). N.S. signifies that there is no statistically significant difference among the compared groups.


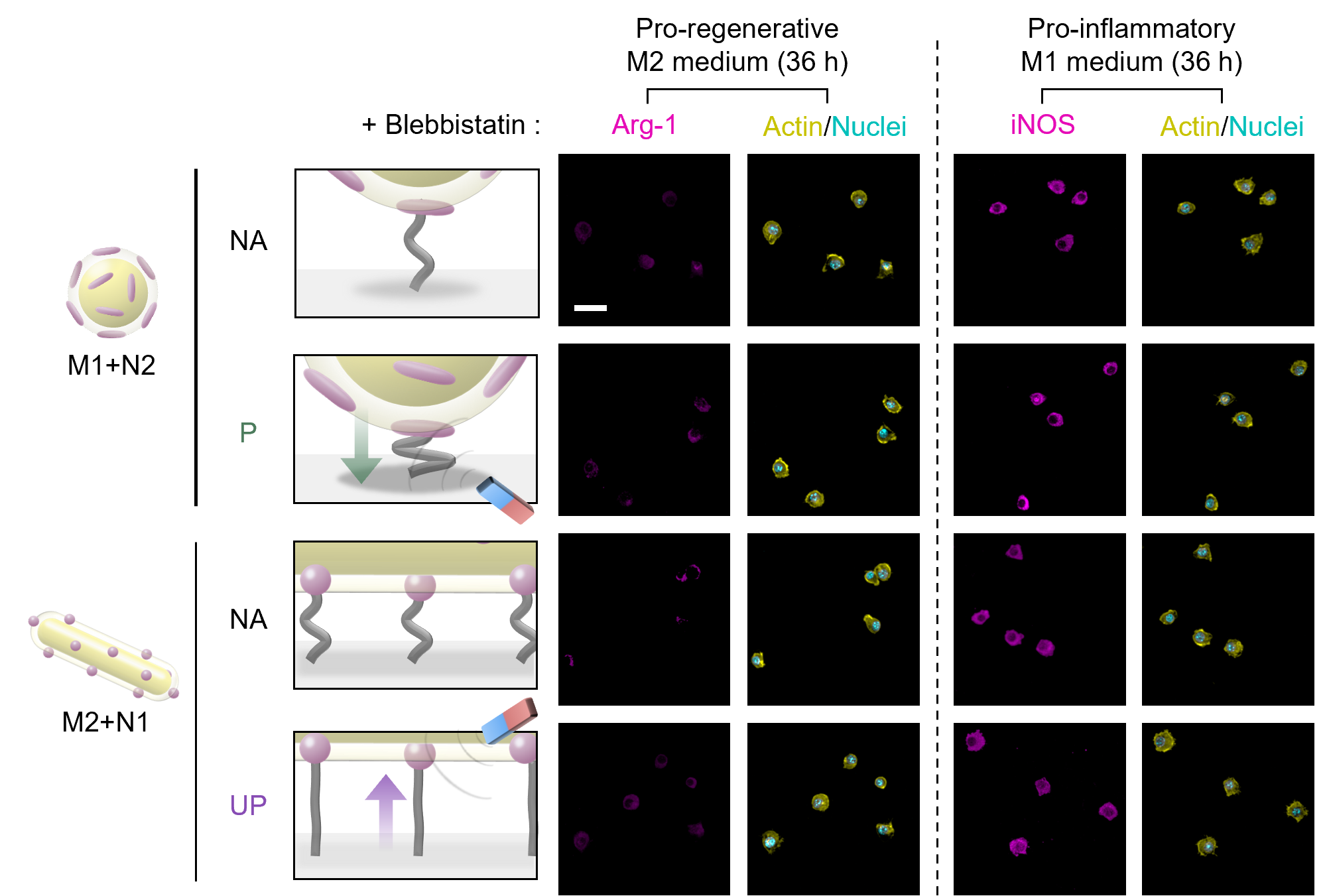


**Fig. S30.** Modulation of macrophage polarization via axial manipulation of hierarchical ligand nanostructures is mediated by myosin II-related molecular mechanisms. Fluorescently immuno-stained images of Arg-1 or iNOS with F-actin/nuclei of the adherent macrophages after 36 h of culturing on the micro-isotropic + nano-anisotropic (“M1+N2”) group in the “non-affected (NA)” or “pressed (P)” states, or on the micro-anisotropic + nano-isotropic (“M2+N1”) group in the “NA” or “unpressed (UP)” state in pro-regenerative M2 or pro-inflammatory M1 medium with the supplementation of the inhibitor specific for myosin II (blebbistatin) (scale bars: 20 µm). The magnet was not placed in the “NA” state or placed either under or over the substrates to induce the “P” or “UP” state, respectively.


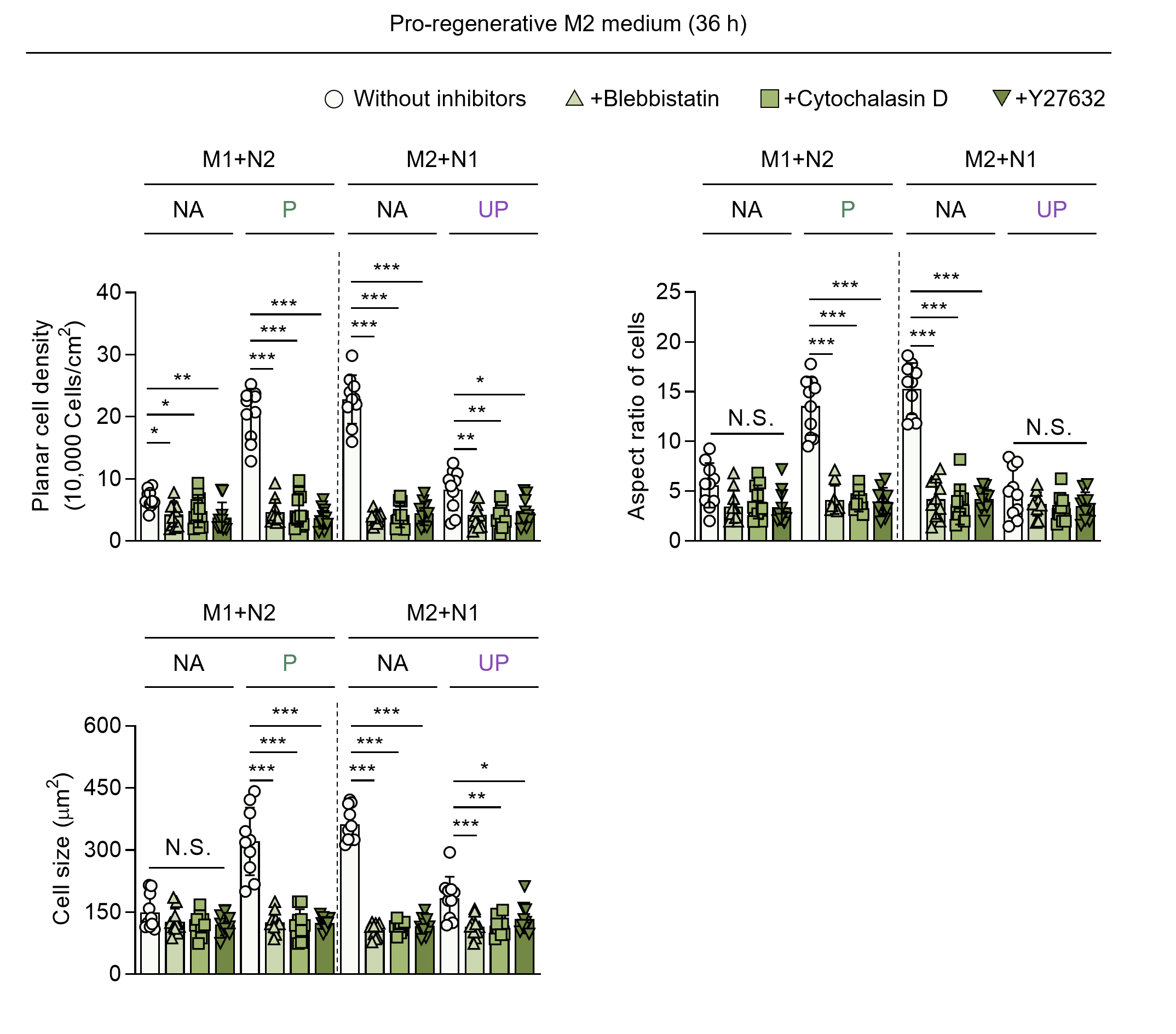


**Fig. S31.** Actin polymerization, myosin II, and ROCK are all involved in the switching of dynamic adhesion-mediated pro-regenerative M2 polarization of macrophages via axial manipulation of multi-scale ligand anisotropy. Calculations of the planar cell density, cell size, and aspect ratio of the adherent macrophages shown in the fluorescently immuno-stained images in Fig. 4a and Fig. S30. The macrophages were cultured for 36 h on the micro-isotropic + nano-anisotropic (“M1+N2”) group in the “non-affected (NA)” or “pressed (P)” states, or on the micro-anisotropic + nano-isotropic (“M2+N1”) group in the “NA” or “unpressed (UP)” states under pro-regenerative medium with or without the supplementation of specific inhibitors for myosin II, actin polymerization, or Rho-associated protein kinase (blebbistatin, cytochalasin D, or Y27632, respectively). The magnet was not placed in the “NA” state or placed either under or over the substrates to induce the “P” or “UP” state, respectively. Data are exhibited as the mean ± standard error (n=10). Asterisks assigned to the range of p values (*: p < 0.1; **: p < 0.01; ***: p < 0.001) represent statistically significant differences. N.S. signifies that there is no statistically significant difference among the compared groups.


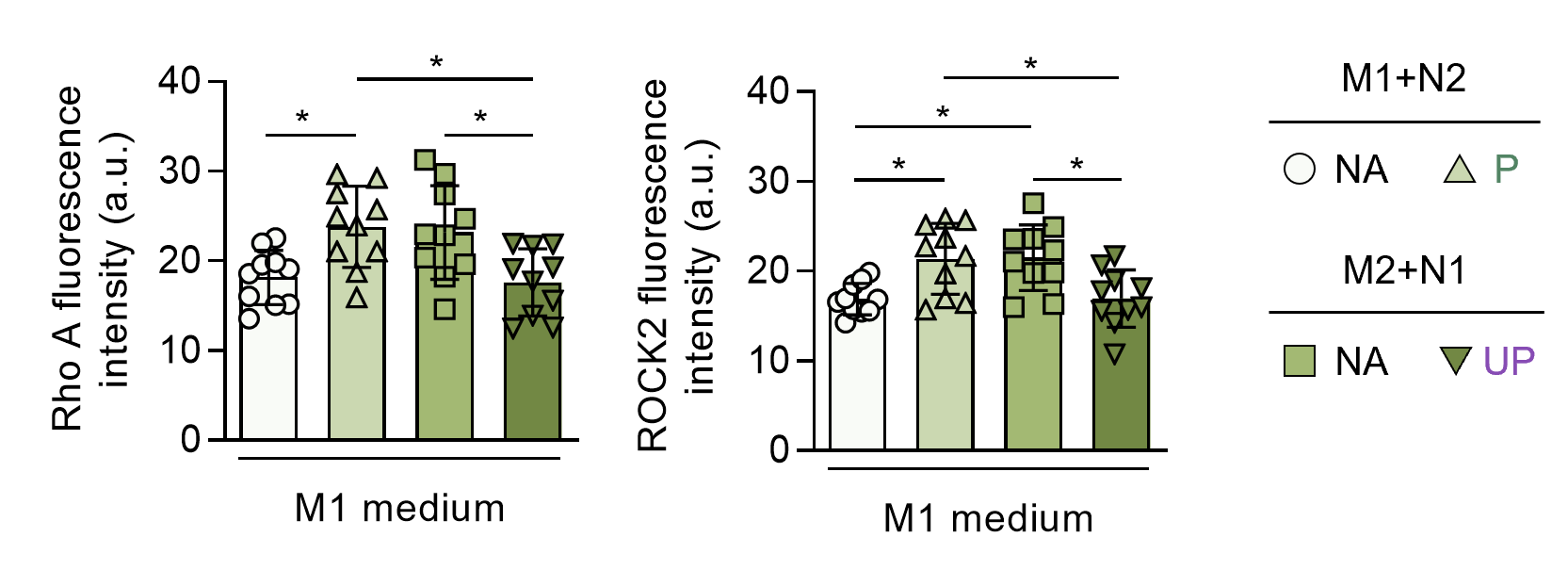


**Fig. S32.** Axial manipulation of hierarchical ligand nanostructures in pro-inflammatory M1 medium dynamically regulates Rho A and ROCK2 signaling in macrophages. Calculations of Rho A and ROCK2 fluorescence intensities of adherent macrophages shown in the fluorescently immuno-stained images in Fig. 4c. The macrophages were cultured for 36 h on the micro-isotropic + nano-anisotropic (“M1+N2”) group in the “non-affected (NA)” or “pressed (P)” states, or on the micro-anisotropic + nano-isotropic (“M2+N1”) group in the “NA” or “unpressed (UP)” states in pro-inflammatory medium. The magnet was not placed in the “NA” state or placed either under or over the substrates to induce the “P” or “UP” state, respectively. Data are exhibited as the mean ± standard error (n=10). Asterisks assigned to the range of p values (*: p < 0.1) represent statistically significant differences.


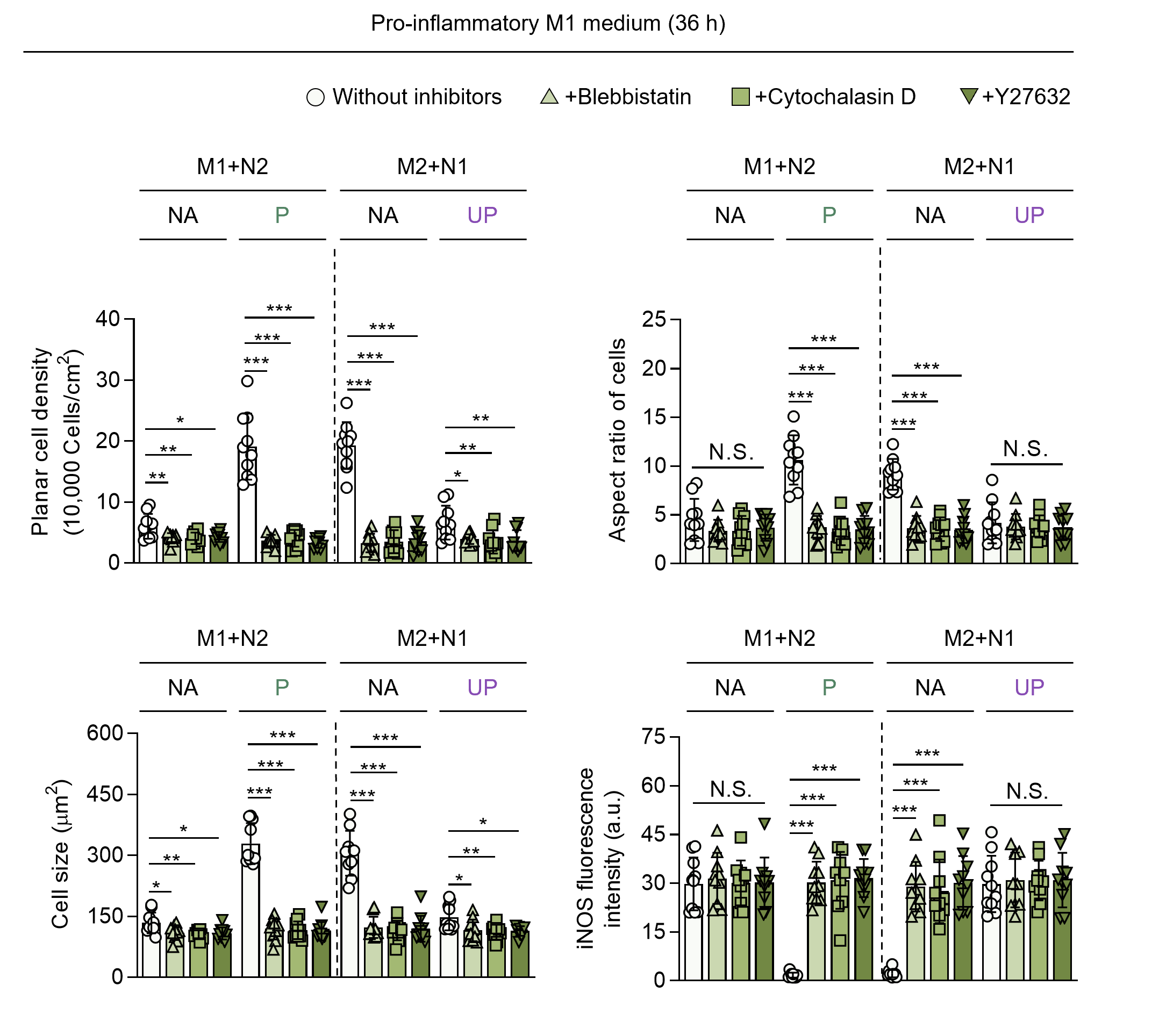


**Fig. S33.** Pro-inflammatory M1 polarization of macrophages under dynamic multi-scale ligand anisotropy involves the suppression of actin polymerization, myosin II, and ROCK. Calculations of the planar cell density, cell size, aspect ratio, and iNOS fluorescence intensity of adherent macrophages shown in the fluorescently immuno-stained images in Fig. 4c and Fig. S30. The macrophages were cultured for 36 h on the micro-isotropic+nano-anisotropic (“M1+N2”) group in the “non-affected (NA)” or “pressed (P)” state, or on the micro-anisotropic+nano-isotropic (“M2+N1”) group in the “NA” or “unpressed (UP)” state in pro-inflammatory M1 medium with or without the supplementation of specific inhibitors for myosin II (blebbistatin), actin polymerization (cytochalasin D), or Rho-associated protein kinase (Y27632). The magnet was not placed in the “NA” state or placed either under or over the substrates to induce the “P” or “UP” state, respectively. Data are exhibited as the mean ± standard error (n=10). Asterisks assigned to the range of p values (*: p < 0.1; **: p < 0.01; ***: p < 0.001) represent statistically significant differences. N.S. signifies that there is no statistically significant difference among the compared groups.


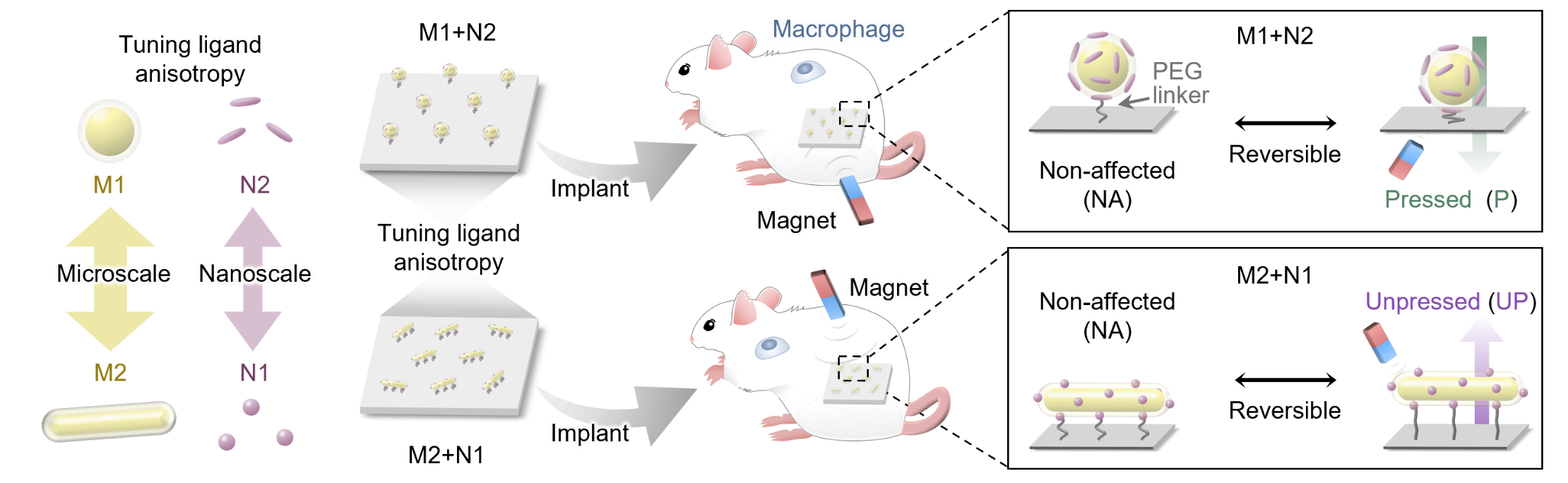


**Fig. S34.** Axial manipulation of multi-scale ligand anisotropy for dynamic host macrophage regulation *in vivo*. Schematic of the regulation of host macrophages in mice via the subcutaneous implantation of the micro-isotropic + nano-anisotropic (“M1+N2”) group under the following “non-affected (NA)” or “pressed (P)” magnetic manipulation, or of the micro-anisotropic + nano-isotropic (“M2+N1”) group under the following “NA” or “unpressed (UP)” magnetic manipulation.


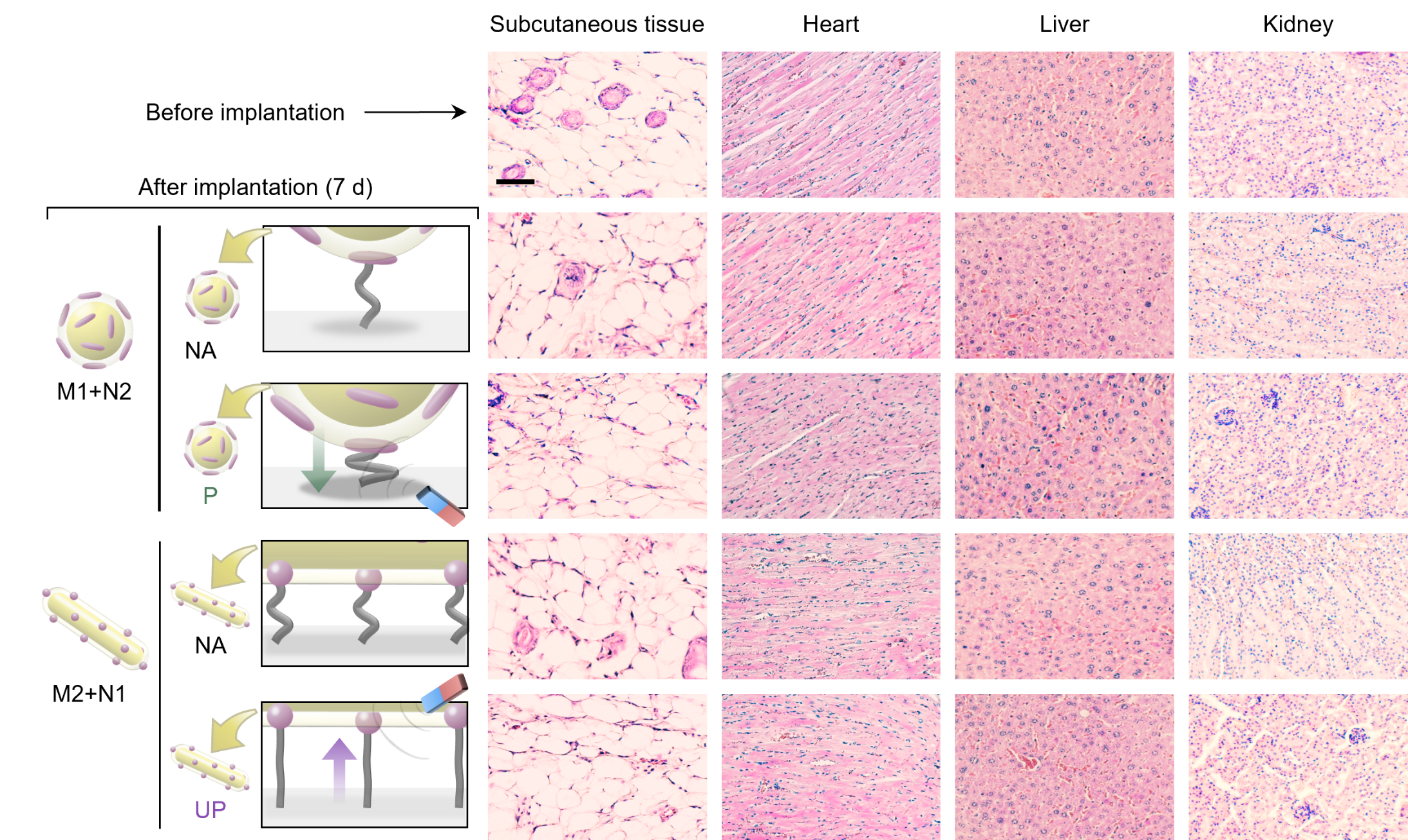


**Fig. S35.** Multi-scale ligand anisotropy-tailored hierarchical nanostructures and their magnetic manipulation are biocompatible. Hematoxylin and eosin (H&E)-stained images of the subcutaneous tissue, heart, liver, and kidney of mice at 0 and 7 d post-implantation (scale bar: 200 µm). The mice were implanted with the micro-isotropic + nano-anisotropic (“M1+N2”) group under the following “non-affected (NA)” or “pressed (P)” magnetic manipulation, or the micro-anisotropic + nano-isotropic (“M2+N1”) group under the following “NA” or “unpressed (UP)” magnetic manipulation. The magnet was not attached in the “NA” state or attached either to the abdomens or backs of the mice to induce the “P” or “UP” state, respectively.


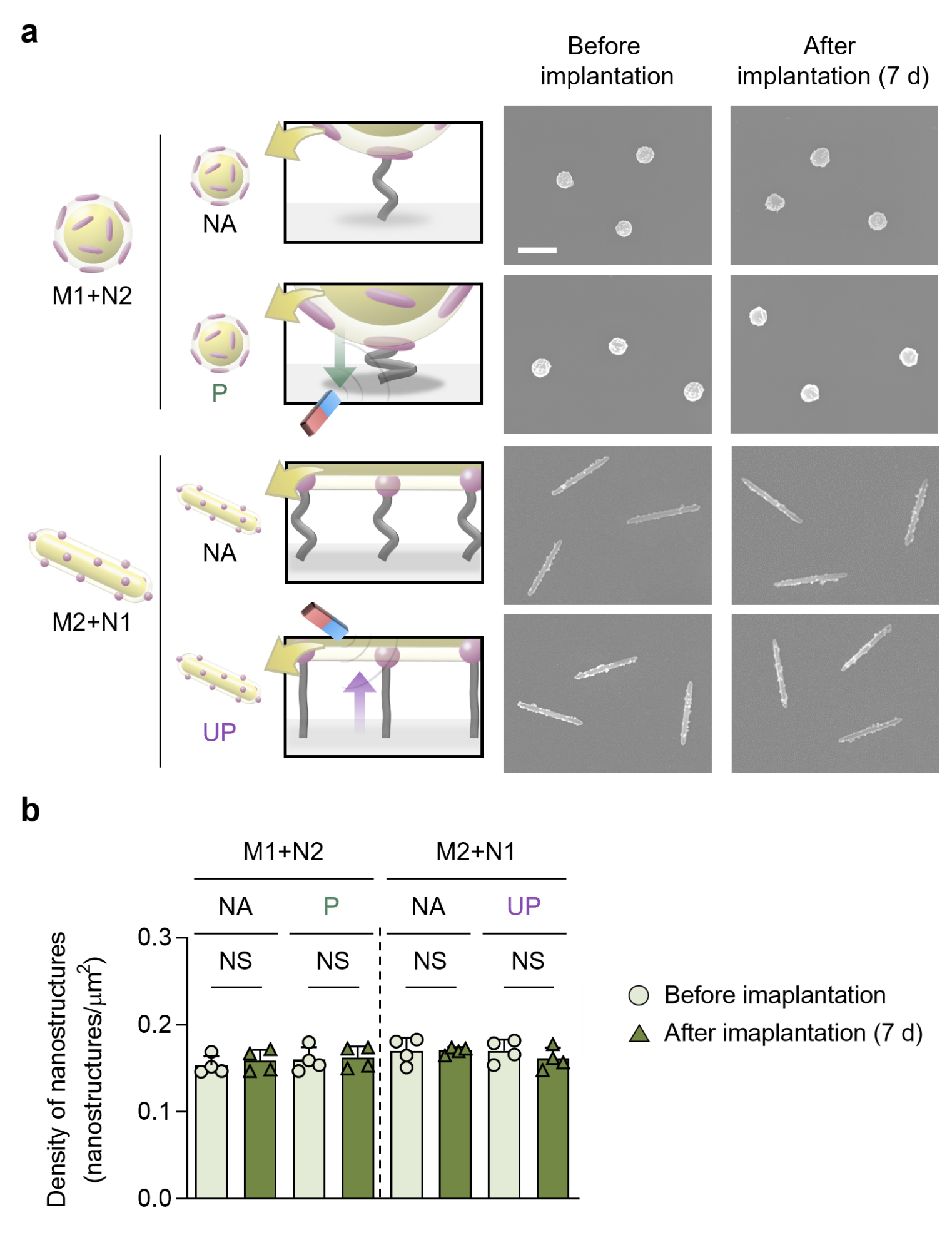


**Fig. S36.** Hierarchical ligand nanostructures are stably coupled on substrate surfaces that maintain the density of nanostructures during the implantation. (a) Scanning electron microscopy (SEM) images of the substrates with the micro-isotropic + nano-anisotropic (“M1+N2”) group under the following “non-affected (NA)” or “pressed (P)” magnetic manipulation, or the micro-anisotropic + nano-isotropic (“M2+N1”) group under the following “NA” or “unpressed (UP)” magnetic manipulation at 0 and 7 d post-implantation (scale bar: 1 µm). (b) Corresponding calculation of the density of each hierarchical nanostructure (Fe_3_O_4_+Au) per μm^2^. The magnet was not attached in the “NA” state or attached either to the abdomens or backs of the mice to induce the “P” or “UP” state, respectively. Data are exhibited as the mean ± standard error (n=4). N.S. signifies that there is no statistically significant difference among the compared groups.


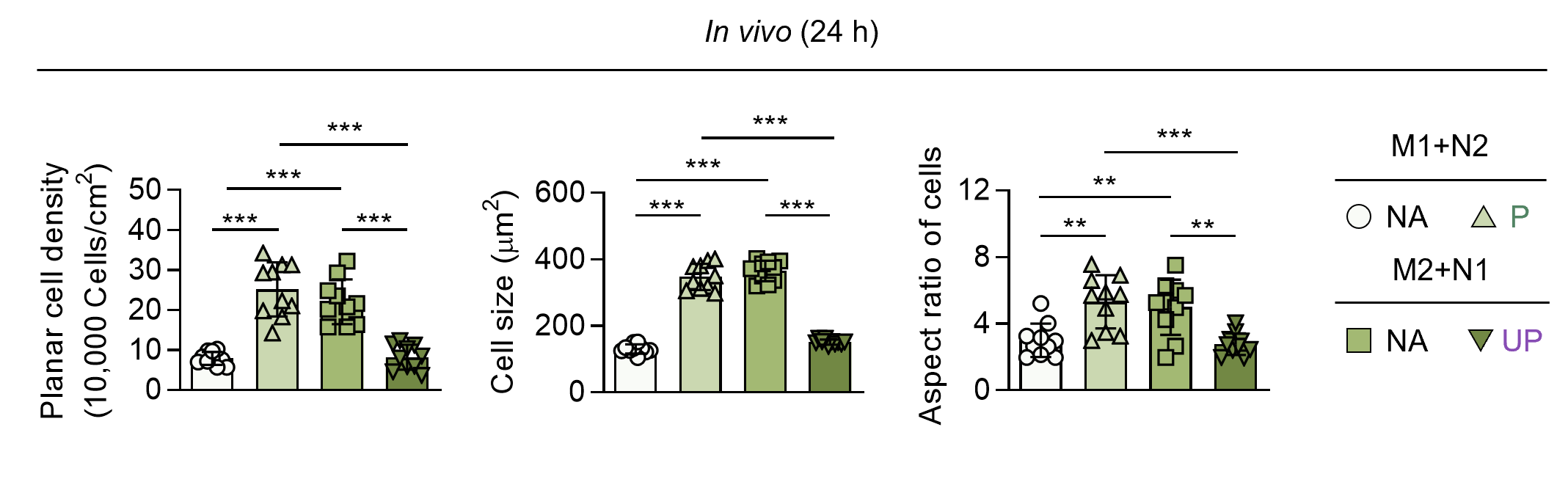


**Fig. S37.** Axial manipulation of the multi-scale ligand anisotropy-tailored hierarchical nanostructures regulates host macrophage adhesion *in vivo*. Calculations of the planar cell density, cell size, and aspect ratio of the adherent host macrophages shown in the fluorescently immuno-stained images in Fig. 5a. The substrates of the micro-isotropic+nano-anisotropic (“M1+N2”) group under the following “non-affected (NA)” or “pressed (P)” magnetic manipulation, or the micro-anisotropic+nano-isotropic (“M2+N1”) group under the following “NA” or “unpressed (UP)” magnetic manipulation were subcutaneously implanted in mice for 24 h. The magnet was not attached in the “NA” state or attached either to the abdomens or backs of the mice to induce the “P” or “UP” state, respectively. Data are exhibited as the mean ± standard error (n=10). Asterisks assigned to the range of p values (**: p < 0.01; ***: p < 0.001) represent statistically significant differences.


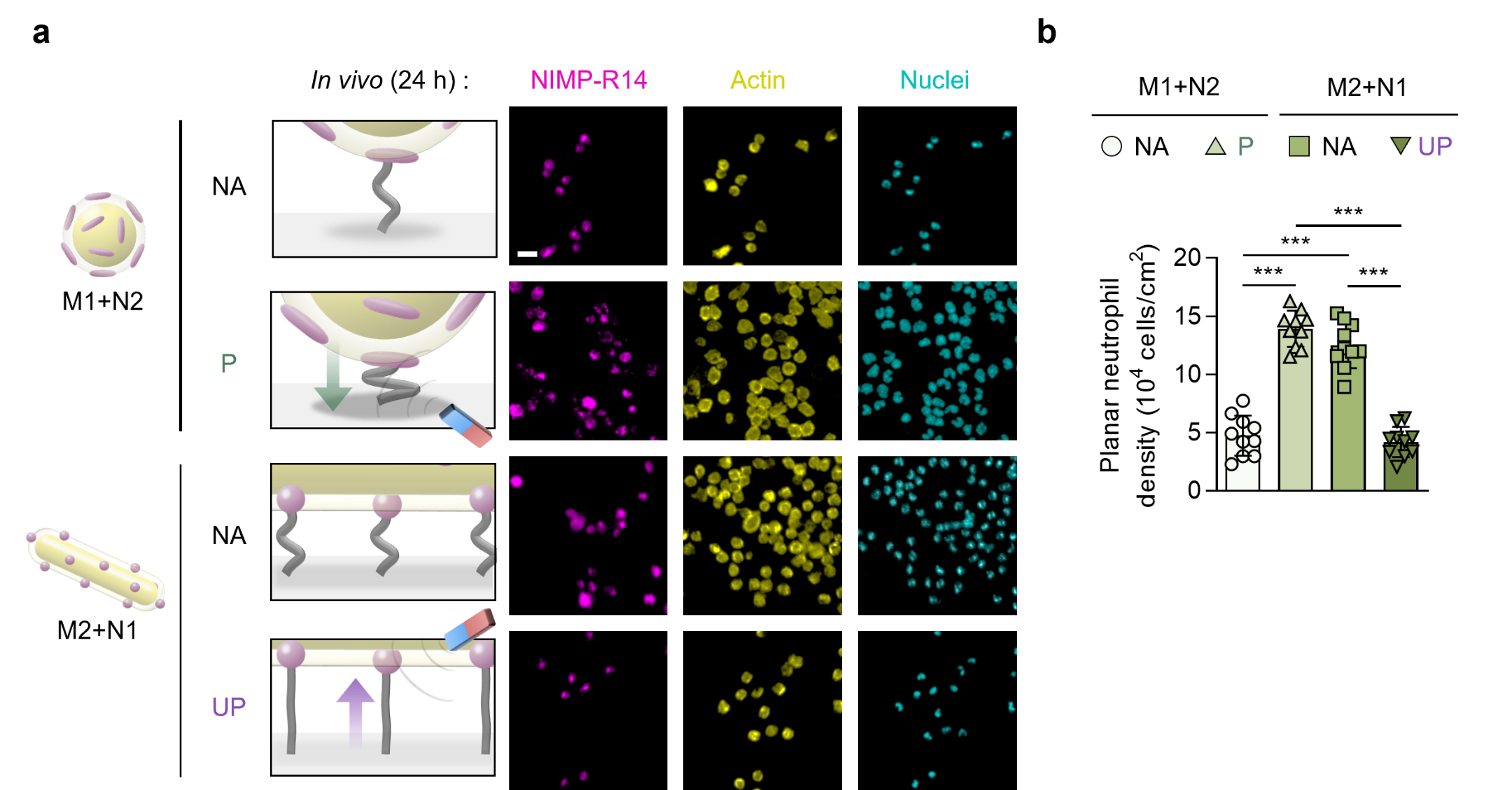


**Fig. S38.** Axial manipulation of the multi-scale ligand anisotropy-tailored hierarchical nanostructures influences the recruitment of host neutrophils *in vivo*. (a) Fluorescently immuno-stained images of NIMP-R14 with F-actin and nuclei of recruited host neutrophils on substrates at 24 h post-implantation of the micro-isotropic + nano-anisotropic (“M1+N2”) group under the following “non-affected (NA)” or “pressed (P)” magnetic manipulation, or the micro-anisotropic + nano-isotropic (“M2+N1”) group under the following “NA” or “unpressed (UP)” magnetic manipulation (scale bar: 20 µm). (b) Following calculation of the planar neutrophil density. The magnet was not attached in the “NA” state or attached either to the abdomens or backs of the mice to induce the “P” or “UP” state, respectively. Data are exhibited as the mean ± standard error (n=10). Asterisks assigned to the range of p values (***: p < 0.001) represent statistically significant differences.

**Table S1.** Morphological characterization of the anisotropy-tailored N-Au particles or M-Fe_3_O_4_ particles used in this research.

| **Particle** | **Particle size (nm)** | **Aspect ratio** |
| --- | --- | --- |
| N1 | Diameter: 44.5 ± 7.3 | 1.1 ± 0.1 |
| N2 | Length: 121.0 ± 3.7; Diameter: 22.6 ± 3.1 | 5.3 ± 0.6 |
| M1 | Diameter: 456.0 ± 61.4 | 1.1 ± 0.1 |
| M2 | Length: 1415.8 ± 28.0; Diameter: 140.4 ± 9.0 | 10.1 ± 1.1 |

**Table S2.** Characterization of multi-scale ligand anisotropy-tailored hierarchical nanostructures for the number of N-Au per M-Fe_3_O_4_ and RGD ligands per N-Au.

| **Group** | **Number of N-Au per M-Fe_3_O_4_** | **Number of RGD per N-Au (10^4^ / N-Au)** |
| --- | --- | --- |
| **M1+N1** | 29.6 ± 2.6 | 28.4 ± 2.8 |
| **M1+N2** | 29.5 ± 1.3 | 28.9 ± 1.3 |
| **M2+N1** | 29.3 ± 1.9 | 28.4 ± 1.3 |
| **M2+N2** | 28.8 ± 2.9 | 29.0 ± 3.4 |
